# Investigating the in vitro steatotic mixture effects of similarly and dissimilarly acting test compounds using an adverse outcome pathway-based approach

**DOI:** 10.1007/s00204-021-03182-1

**Published:** 2021-11-15

**Authors:** Jimmy Alarcan, Georges de Sousa, Efrosini S. Katsanou, Anastasia Spyropoulou, Petros Batakis, Kyriaki Machera, Roger Rahmani, Alfonso Lampen, Albert Braeuning, Dajana Lichtenstein

**Affiliations:** 1 Department Food Safety, German Federal Institute for Risk Assessment, Max-Dohrn-Str. 8-10, 10589 Berlin, Germany; 2 Institut Sophia Agrobiotech, Université Côte d’Azur-INRAE-CNRS, 06903 Sophia Antipolis, France; 3 Benaki Phytopathological Institute, Athens, Greece

**Keywords:** Steatosis, Mixtures, AOP-wise testing, Relative potency factors, Triglyceride accumulation, Hepatotoxicity

## Abstract

Within the EuroMix project, we have previously developed an adverse outcome pathway (AOP)-based in vitro assay toolbox to investigate the combined effects of liver steatosis-inducing compounds in human HepaRG hepatocarcinoma cells. In this study, we applied the toolbox to further investigate mixture effects of combinations, featuring either similarly acting or dissimilarly acting substances. The valproic acid structural analogs 2-propylheptanoic acid (PHP) and 2-propylhexanoic acid (PHX) were chosen for establishing mixtures of similarly acting substances, while a combination with the pesticidal active substance clothianidin (CTD) was chosen for establishing mixtures of dissimilarly acting compounds. We first determined relative potency factors (RPFs) for each compound based on triglyceride accumulation results. Thereafter, equipotent mixtures were tested for nuclear receptor activation in transfected HepG2 cells, while gene expression and triglyceride accumulation were investigated in HepaRG cells, following the proposed AOP for liver steatosis. Dose addition was observed for all combinations and endpoints tested, indicating the validity of the additivity assumption also in the case of the tested mixtures of dissimilarly acting substances. Gene expression results indicate that the existing steatosis AOP can still be refined with respect to the early key event (KE) of gene expression, in order to reflect the diversity of molecular mechanisms underlying the adverse outcome.

**Supplementary Information:**

The online version contains supplementary material available at 10.1007/s00204-021-03182-1.

## Introduction

Human exposure to countless chemical substances, occurring as complex mixtures, has become a major concern for regulatory agencies (Escher et al. 2017; Rappaport and Smith 2010). In response to this issue, different tiered strategies for the risk assessment of combined exposure to multiple chemicals have been proposed and implemented (Rotter et al. 2018). The identification of the modes of action (MoAs) of the different components inside a mixture is an important task to perform, as such information permits to distinguish between similarly and dissimilarly acting compounds. Compounds sharing the same MoA are believed to follow the principle of dose addition when being in mixtures, and, therefore, mixture effects may be predicted by the dose addition concept (Backhaus and Faust 2012; Kortenkamp et al. 2009). However, in the case of compounds with a different MoA, it is unclear whether the assumption of dose addition is still valid (Borgert et al. 2012; EFSA et al. 2013; Kortenkamp et al. 2009). Thus, the dose addition assumption in the case of mixtures involving substances with dissimilar MoA remains to be investigated on a larger scale.

**Fig. 1:**
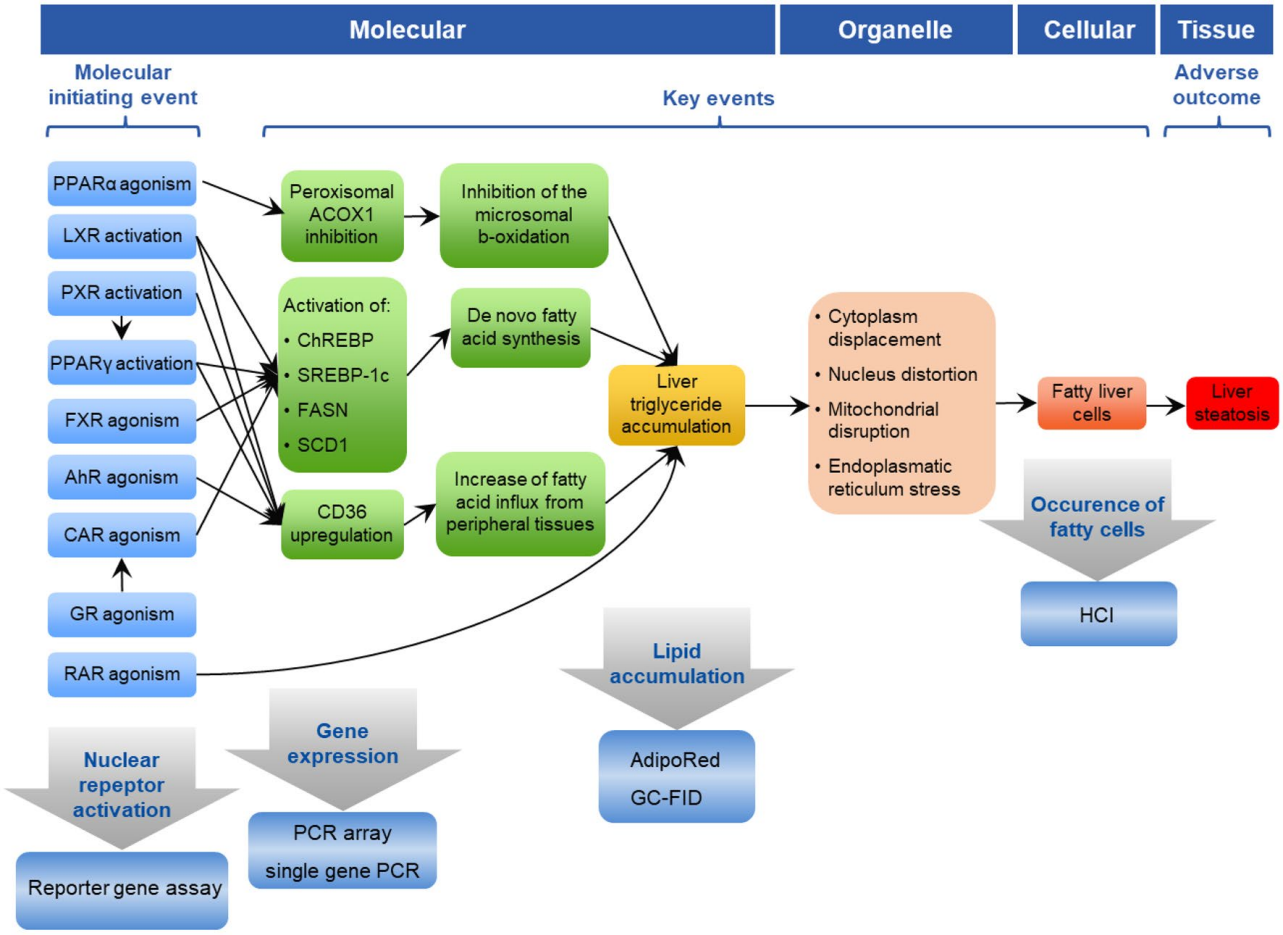
Liver steatosis AOP (modified after Luckert et al. (2018)). Biological events analyzed in the present study are indicated by arrows

In a previous study, we have established an in vitro bioassay toolbox to evaluate different endpoints along the adverse outcome pathway (AOP) for chemically induced liver steatosis (Luckert et al. 2018). In short, the activation of nuclear receptors (NRs) is described as the molecular initiating events (MIEs), leading to induction of further key events (KEs) including specific gene transcription and expression of proteins that subsequently induce the accumulation of liver triglycerides. This accumulation triggers different toxic events at the organelle level which leads to fatty liver cells and ultimately to the biological adverse outcome (AO) steatosis (Fig. 1). Using the toolbox, we have previously investigated mixture effects of different combinations of pesticides, using imazalil, thiacloprid and clothianidin (CTD) as test compounds. Triglyceride accumulation was observed for all pesticides, alone and in mixtures (Lichtenstein et al. 2020). The three binary and one ternary mixtures that were tested all showed dose addition for the different investigated endpoints. However, due to overlap in the NRs agonism/antagonism patterns of the three substances, it was not possible to draw conclusions on the testing of mixtures with compounds showing a strictly dissimilar MoA. Therefore, in the present study, we designed new mixtures with a strong emphasis on the aspect of similarly/dissimilarly acting compounds. CTD was included again as it showed a very distinct pattern in the activation of NRs, as compared to other steatosis-inducing compounds, targeting only PPARα (antagonism) (Lichtenstein et al. 2020). Thus, we screened for compounds with a known steatotic potential, which interact with NRs excluding PPARα antagonism.

**Fig. 2:**
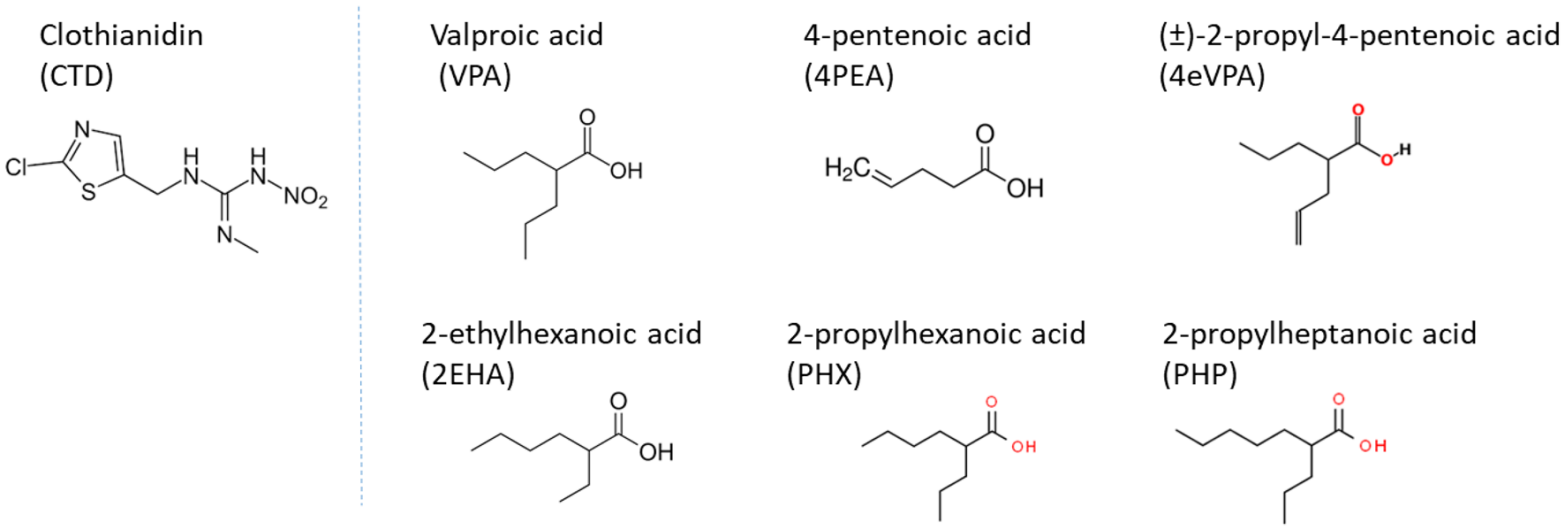
Chemical structures of CTD, VPA and its analogs

Valproic acid (VPA) is one of the most commonly used drugs to treat epilepsy, but can lead to undesired hepatotoxicity such as steatosis (Chang et al. 2016). Fatty acid metabolism impairment following in vitro treatment with VPA has been also reported (Grünig et al. 2020). Moreover, VPA was shown to activate the pregnane X receptor (PXR) and constitutive androstane receptor (CAR) in luciferase reporter assays (Cerveny et al. 2007). Both receptors are crucially involved in liver steatosis and possible MIE for the steatosis AOP (Mellor et al. 2016). Besides, several structural analogs of VPA are available, making it possible to screen for a panel of molecules that presumably induce comparable toxicity. In regard to these findings, CTD, VPA and its analogs (Fig. 2) were considered promising candidates for the implementation of mixtures consisting of similarly and dissimilarly

acting compounds.

Equipotent mixtures were designed based on the compound-specific relative potency factors (RPF) that were determined with lipid accumulation data in HepaRG cells, using a benchmark dose (BMD) approach. HepaRG cells represent a relevant model to study hepatic steatosis in vitro, as they show lipid accumulation in response to treatment with known steatosis-inducing chemicals, and furthermore they functionally express key steatosis-related nuclear receptors (Antherieu et al. 2012; Luckert et al. 2018; Tanner et al. 2018; Tolosa et al. 2016). NRs activation, target gene regulation and triglyceride accumulation were further analyzed for the mixtures (see Fig. 1). The findings of this study extend our knowledge on the behavior of chemical mixtures and provide new experimental data on mixtures of similarly and dissimilarly acting compounds.

## Materials and methods

### Chemicals

VPA (CAS no. 1069-66-5), 4PEA (CAS no. 591-80-0), 4eVPA (CAS no. 1575-72-0), 2EHA (CAS no. 149-57-5), PHP (CAS no. 31080-39-4) and CTD (CAS no. 210880-92-5) were obtained from Sigma Aldrich (St. Louis, USA). PHX (CAS no. 3274–28-0) was purchased from Toronto Research Chemicals (Toronto, Canada). Cyproconazole (CAS no. 94361-06-5) was purchased from Syngenta (Basel, Switzerland) as technical grade. All other chemicals were obtained from Sigma (Taufkirchen, Germany) or Merck (Darmstadt, Germany) in the highest available purity.

### Cell culture

Human HepaRG hepatocarcinoma cells were cultivated as previously described in Lichtenstein et al. (2020). Briefly, cells were purchased from Biopredic International (Saint Grégoire, France) and cultivated in William’s Medium E with 2 mM glutamine (PAN-Biotech, Aidenbach, Germany), 10% (v/v) bovine serum (FBS; FBS Good Forte EU approved, PAN-Biotech, Aidenbach, Germany), 100 U/ml penicillin and 100 µg/ml streptomycin (Capricorn Scientific, Ebsdorfergrund, Germany) and 5 × 10^–5^ M hydrocortisone hemisuccinate (Sigma-Aldrich, St. Louis, USA) at 37 °C in a humidified atmosphere with 5% CO_2_. Cells (passages 15 to 20) were seeded at a density of ~ 25,000 cells/cm^2^ and grown for 14 days, followed by 14 days in medium additionally containing 1.7% DMSO. Differentiated HepaRG cells were pre-adapted to treatment medium (culture medium containing only 2% FBS and 0.5% DMSO) for 48 h prior to exposure in treatment medium for 24 h or 72 h, with a final DMSO concentration of 0.5%.

Human HepG2 hepatocellular carcinoma cells were purchased from the European Collection of Cell Cultures (ECACC #85,011,430, Salisbury, UK) and cultured in Dulbecco’s modified Eagle’s medium (DMEM, PAN-Biotech, Aidenbach, Germany) supplemented with 10% (v/v) fetal calf serum (PAN-Biotech, Aidenbach, Germany), 100 U/ml penicillin and 100 µg/ml streptomycin (Capricorn Scientific, Ebsdorfergrund, Germany) at 37 °C in a humidified atmosphere with 5% CO_2_. Cells were passaged at ~ 80–90% confluence and seeded at a density of 60,000 cells/cm^2^. Only cells within passages 15–25 were used.

### Cell viability analysis

Cytotoxic effects of test compounds were analyzed in HepG2 and HepaRG cells using the WST-1 assay (Sigma-Aldrich, St. Louis, USA) as described by Luckert et al. (2018). At least two independent biological replicates with minimum three technical replicates per condition were run.

### AOP-based approach

In this study we evaluated different components of the liver steatosis AOP. As shown in Fig. 1, NR activation was investigated as part of MIE while gene expression and lipid accumulation were studied as part of molecular and cellular KEs. Considering this AOP-based approach, we applied different time treatments (i.e. 24 h for NR activation and gene expression, 72 h for lipid accumulation) to reflect the temporal relationships between biological events (e.g. upstream MIEs occur earlier than subsequent downstream KEs). This temporal relationship was also shown experimentally as for instance lipid accumulation was better observed after 72 h treatment cyproconazole than 24 h treatment in the study from Luckert et al. (2018).

### Reporter gene assays

The activation of 10 NRs (CAR, farnesoid X receptor (FXR), glucocorticoid receptor (GR), liver X receptor (LXR) α, peroxisome proliferator-activated receptor (PPAR) α, PPARγ, PPARδ, PXR, retinoic acid receptor (RAR) α, retinoid X receptor (RXR) α) and aryl hydrocarbon receptor (AhR) by test compounds was investigated using reporter gene assays. Due to low transfection efficacy in HepaRG cells, reporter gene analysis was performed using HepG2 cells. A detailed overview of the plasmids and transfection methodology can be found in the paper by Luckert et al. (2018). HepG2 cells were seeded in 96-well plates and transiently transfected after 24 h using TransIT-LT1 (Mirus Bio, Madison, USA) according to the manufacturer’s instructions. An overview of the specific conditions for each reporter gene assay (plasmid, plasmid amount, positive controls) can be found in supplementary Table S1. Cells were exposed to different concentrations of the test compounds for 24 h in culture medium containing 0.5% DMSO. Cell lysis and luminescence measurements were performed on an Infinite M200 Pro (Tecan group, Männedorf, Switzerland) luminometer. Three independent biological replicates were run, each in three technical replicates per condition.

### Analysis of mRNA expression levels

HepaRG cells were differentiated in 12-well plates and treated with different concentrations of the test compounds or solvent control (0.5% DMSO) for 24 h. Cells were washed twice with ice-cold PBS and lysed with 350 µl RLT buffer (RNeasy Mini Kit, Qiagen, Hilden Germany). Total RNA was extracted according to the manufacturer protocol. For first-strand cDNA synthesis, 1 μg of total RNA was reverse-transcribed into cDNA in a total volume of 20 μl, using the PrimeScript RT reagent Kit Perfect Real Time for RT-PCR (Takara Bio, Europe) with oligo-dT primers and random hexamers for the reaction according to the manufacturer’s instructions. Specific primers were designed using the Primer-BLAST tool (NIH) for 63 genes linked to liver steatosis, nuclear receptor activation and hepatotoxicity. Primers for three reference genes were also designed (*B2M, GAPDH* and *ACTB)*. EC_50_ values, derived from AdipoRed experiments, were used for a first screening of gene deregulation following single compound treatment. Genes exhibiting deregulation at the EC_50_ concentration (screening process) as well as the five genes of the steatosis AOP (*ACOX1, FASN, MLXIPL, SCD1, SREBF1*) were finally assessed at all chosen single compound concentrations. Regarding mixture testing, genes showing pronounced responses to treatment in at least two compounds in the screening process, as well as all 5 AOP genes, were selected to be assessed at all mixture concentrations. The oligonucleotide sequences of primers are shown in supplementary Table S2. To prevent amplification of sequences from genomic DNA contamination, primers and/or amplicons were designed to cross exon/exon boundaries if possible. All genes were amplified by real-time PCR in the Step One Plus detection system with StepOnePlus Software v2.3 (Thermo Fisher Scientific, Waltham, USA) using SYBR green as the detection dye. Each amplification reaction was carried out in a total volume of 20 μl containing 10 μl SYBR Select Master Mix (Thermo Fisher Scientific), 0.3 μM of each primer and 0.002 μg cDNA. The reactions were cycled 40 times using the following parameters: 95 °C for 3 s and 60 °C for 30 s during which the fluorescence data were collected. Melting curves were generated to verify the identity of the products. A non-template control was run with every set of primers and no indication of PCR contamination was observed. Lack of PCR products from the non-reverse transcribed RNA control indicated that contamination by genomic DNA did not serve as amplification template. Expression levels of the target genes were normalized to the reference gene *B2M* (beta-2-microglobulin) which was stably expressed throughout treatments. RNA from three independent biological replicates was used. Each cDNA was analyzed at least in duplicate by real-time PCR. Relative gene expression was calculated using the ΔΔCT method (Livak and Schmittgen 2001). The statistical calculation was based on 2^−ΔCt^ values.

### Liver triglyceride accumulation

Liver triglyceride accumulation was measured using three different methodologies. The Adipored assay and Nile Red staining analysis by high-content cell imaging (HCI) use the same dye, i.e. Nile red, but differ in their procedure. The Adipored assay measures the total fluorescence intensity inside a well, while HCI quantifies the lipid droplets (size, intensity, number), thus allowing a phenotypic vision at the single-cell level. In complement, triglyceride was analyzed using gas chromatography with flame-ionization detection (GC-FID) method.

### AdipoRed assay

HepaRG cells were treated for 72 h with different concentrations of the test compounds, solvent (0.5% DMSO), or a positive control for steatosis (cyproconazole; 200 µM). Then, the cell monolayer was rinsed with 200 µl phosphate-buffered saline (PBS) and nuclei were stained with 5 µg/ml Hoechst 33,342 (Thermo Fischer Scientific, Waltham, USA). Afterwards, 5 µl/well AdipoRed solution (ready to use; Lonza, Basel, Switzerland) was added and cells were incubated for 10 min at 37 °C. Fluorescence was measured at Ex 485 nm/Em 572 nm for AdipoRed and Ex 350 nm/Em 461 nm for Hoechst 33,342 staining using an Infinite M200 Pro plate reader (Tecan group, Männedorf, Switzerland). Relative triglyceride levels were referred to solvent control. Four independent biological replicates with minimum three technical replicates per condition were run.

### Triglyceride extraction and analysis by gas chromatography with flame-ionization detection (GC-FID)

Triglyceride analysis by GC-FID was performed as previously described (Lichtenstein et al. 2020). Briefly, HepaRG cells were differentiated in 12-well plates and treated with different concentrations of the test compounds or solvent control (0.5% DMSO) for 72 h. Then, the monolayer was rinsed with 1 ml Dulbecco’s phosphate-buffered saline (DPBS)/well followed by harvesting in 300 µl RLT-lysis buffer (Qiagen, Venlo, The Netherlands). The lysates were collected and stored at − 80 °C until triglyceride extraction. After thawing, the organic phases were collected using a mixture of isooctane and ethylacetate (75:25, 5 ml) with tritridecanoin (Nu-Chek Prep Inc., Elysian, USA). The organic phases were dried under N_2_ gas, redissolved in 100 µl isooctane and transferred to a GC vial for analysis. Analysis of the samples was executed on a Trace GC Ultra GC-FID system (Thermo Fisher Scientific, Waltham, USA). Quantification of the triglycerides was achieved by determining the area under the curve (AUC). Calculation of the relative triglyceride level was achieved by dividing the AUC of the test compounds by the AUC of the solvent control. Two independent biological replicates with three technical replicates per condition were run.

### Nile Red staining and neutral lipid droplet analysis by high-content cell imaging (HCI)

HepaRG cells were treated for 72 h with different concentrations of the test compounds, solvent (0.5% DMSO), or a positive control (20 µM cyclosporine A). Afterwards, cells were fixed in 4% (w/v) paraformaldehyde in PBS for 30 min and washed three times with PBS and stained overnight (at 4 °C) with 100 µl of 0.9 µM Nile Red (Sigma-Aldrich, St. Louis, USA) solution in PBS (Amacher and Martin 1997; McMillian et al. 2001). Nuclei were stained with DAPI (0.3 µg/ml in PBS) during 2 hours before reading (incubation at ambient temperature in the dark). The multi-well plates were scanned (9 images) with an Arrayscan XTI using a 20 × NA 0.4 objective (Plan NeoFluar, Zeiss, Oberkochen, Germany). The Photometrics X1 CCD camera was set with a binning 2 (14 bits dynamic range, 4 × 106 pixels with a size of 4.54 µm). Identification of neutral lipid spot was done by tracking Nile Red green emission with an XF100-485-20 filter set. Identification of the nuclei was done by tracking DAPI with an XF100-386-23 filter set and used to focus the instrument. The Spot Detector V3 Bioapplication analysis algorithm (software V.6.5) was used to identify nuclei upon fluorescent size and intensity. The nuclear mask was dilated in order to define the cytoplasmic region. Two parameters were measured at the cell level as follows: nuclei (defined as area as well as total and average intensity for each cell) and neutral lipid spots (defined as spot number, spot intensity and spot area as well as total fluorescence of spot intensity within each cell).

To quantitatively assess the data obtained after the image analysis, a workflow was built in Statistica v13.2 (Tibco, Palo Alto, USA). First, each independent plate was standardized in order to eliminate inter-experiment variation. In order to fix the values in the same order of magnitude (robust Z-score) the whole data set for neutral lipid spot total intensity within cells was submitted to a median MAD standardization. Finally, the three independent experiments were grouped and data were then normalized to the median of solvent controls (value defined as 1).

### Analysis of mixture effects and compound potencies

Concentration–response modeling and RPF analysis were performed as previously described (Lichtenstein et al. 2020). Data were loaded into PROAST (https://www.rivm.nl/en/proast, RIVM, Bilthoven, The Netherlands). Concentration–response data were statistically analyzed by fitting with an exponential four-parameter model (1):

1 $$y=a\left[c-\left(c-1\right)\mathrm{exp}\left(-b{x}^{d}\right)\right]$$

where *y* denotes the response, *x* denotes the concentration, a reflects the response at concentration zero, b relates to the potency of the tested chemicals, *c* reflects the maximum response and *d* reflects the steepness of the curve. Based on the obtained fits, RPFs were calculated for a benchmark response of 50% (BMR_50_). Mixture compositions were thereafter determined based on the estimated RPFs to design equipotent mixtures of the test compounds.

For mixture effect analysis, the concentration–response data for the mixture and the single compounds were compared using the same approach. First, the single compounds were analyzed for calculation of the RPF. The curve fit results were expressed visually as described above. If the data points of the mixture fit with the curve derived from the single compounds, dose addition can be assumed. In cases of synergism or antagonism, the concentration–response data of the mixture will not fit with the response of the single substances and shift either to the left or to the right. Additionally, the ratio of overlap was calculated to provide a quantitative evaluation. The ratio of overlap is used to characterize the degree of dose addition or the degree of deviation from dose addition and describes numerically what can be seen graphically in the PROAST plots. Confidence intervals of the estimated RPFs were calculated for the single compounds and also for the corresponding mixtures. If both intervals overlap, then the response curves are very close together and dose addition can be assumed. On the contrary, if intervals do not overlap then the respective response curves are very far away from each other and dose addition cannot be assumed (visually apparent on the plot). Thus, the lower confidence limit of the benchmark dose (BMDL) of the higher calculated interval was divided by the upper confidence limit of the benchmark dose (BMDU) of the lower calculated interval. A ratio of overlap above 1 indicates a deviation from dose addition (i.e. no overlap of the confidence intervals), while a ratio below 1 indicates no deviation from dose addition (i.e. overlap of the confidence intervals).

### Statistical analysis

All statistical analysis were performed with GraphPad Prism v.8 (GraphPad Software, La Jolla, USA). Statistical analysis for cell viability, nuclear receptor and triglyceride accumulation endpoints was performed by doing one-way ANOVA followed by Dunnett’s test (*p < 0.05; **p < 0.01; ***p < 0.001). Statistical analysis for gene regulation endpoint was performed by doing the non-parametric Kruskal–Wallis test followed by Dunn’s test (*p < 0.05; **p < 0.01; ***p < 0.001).

## Results

### Screening of VPA and its analogs

#### Concentration-range finding and liver triglyceride accumulation

Based on cell viability assay results, maximal concentrations of 6 mM 4PEA, 4eVPA, 2EHA, PHX, PHP and 4 mM VPA were chosen for subsequent experiments in HepaRG cells in order to exclude unspecific cellular responses due to pronounced cytotoxicity (> 25%) (Supplementary Fig. S1). Similarly, maximal concentrations of 4 mM VPA, 4eVPA, 2EHA, PHX, PHP and 5 mM 4PEA were chosen for subsequent experiments in HepG2 cells in order to exclude unspecific cellular responses due to pronouced cytotoxicity (> 25%) (Supplementary Fig. S2). In a next step, triglyceride accumulation in HepaRG cells was measured via AdipoRed staining (Fig. S3). All compounds induced lipid accumulation but with different potencies: 4PEA > PHP/PHX > 2EHA > VPA/4eVPA. CTD cytotoxicity and triglyceride accumulation was also measured and was not toxic up to 1000 µM. CTD induced lipid accumulation in a concentration-dependent manner (Figs. S1–S3).

#### Nuclear receptor activation screening and selection of the test compounds

Activation of AhR, CAR, FXR, GR, LXRα, PPARα, PPARγ, PPARδ, PXR, RARα and RXRα was monitored using luciferase-based reporter assays in human HepG2 cells. VPA and its analogs activated AhR, CAR, FXR, GR, PPARα, PPARy and RXRα with different potencies. Of note, PHP and PHX strongly activated PPARα (80-fold activation and 50-fold activation, respectively) and PPARy (180-fold activation and 120-fold activation, respectively). On the contrary, LXRα was whether unaffected (VPA and 4PEA) or inhibited (4eVPA: 2.5-fold reduction, 2EHA: twofold reduction, PHP: fivefold reduction, PHX: 3.3-fold reduction) (Table 1). PPARδ was activated solely by PHP (twofold activation) and PHX (twofold activation). CTD only affected PPARα, antagonizing its activity (3.3-fold reduction). Results on concentration-dependent induction of reporter activities can be found in supplementary data section (Figs. S4–S15).

**Table 1:** Nuclear receptor activation pattern of VPA, 4PEA, 4eVPA, 2EHA, PHX, PHP and CTD

Assay	VPA	4PEA	4eVPA	2EHA	PHP	PHX	CTD
AhR	↑	↑	↑	↑	↑	↑	–
CAR	↑	↑	↑	↑	↑	↑	–
FXR	↑	↑	↑↑	↑↑	↑↑	↑↑	–
GR	↑↑	↑	↑	↑	↑↑	↑	–
LXRα	–	–	↓	↓	↓	↓	–
PPARα	↑↑	↑	↑↑	↑↑↑	↑↑↑	↑↑↑	↓
PPARγ	↑↑	↑↑	↑↑	↑↑	↑↑↑	↑↑↑	–
PPARδ	–	–	–	–	↑	↑	–
PXR	↑	–	–	↑	↑	↑	–
RARα	↑	↓	↑	↓	↑	–	–
RXRα	↑	↑	↑	↑	↑↑	↑	–

Symbols: ↑↑↑, fold activation > 50; ↑↑, 10 < fold activation < 50; ↑, fold activation < 10; ↓, inhibition; –, no effect

#### Design of equipotent mixtures

According to the steatosis AOP, activation of NRs (e.g. PPARs) leads to biological events that subsequently induce the accumulation of liver triglycerides. Our screening assays showed that 4PEA, PHP and PHX induced the strongest lipid accumulation, but 4PEA activated PPARα and PPARγ only to a weak or moderate extent. On the contrary, PHP and PHX strongly activated PPARα and PPARy and showed very high overlap in their NR activation pattern. Therefore, PHP and PHX were defined as compounds acting in a similar MoA in contrast to CTD that was defined as a compound acting in a dissimilar MoA as compared to PHP and PHX. Based on lipid accumulation data in HepaRG cells (AdipoRed), RPFs were calculated with the dose–response modeling software PROAST by comparing the whole curves of each compound (Fig. S16). PHX was the least potent inducer of triglyceride accumulation and was, therefore, assigned a RPF value of 1. CTD was 4.5 times more potent than PHX, while PHP was 1.3 times more potent. CTD was 3.6 times more potent than PHP. These RPFs were used to design equipotent mixtures for subsequent gene expression and lipid accumulation experiments. Table 2 recapitulates all the treatment conditions. For reporter gene assay experiments, equipotent mixtures were designed using the same RPFs but with adapted concentration range (Table S3).

**Table 2:** Equipotent binary and ternary mixtures of PHP, PHX, and CTD

Label	PHX + PHP			PHX + CTD		
	PHXeq [µM]	PHX [µM]	PHP [µM]	PHXeq [µM]	PHX [µM]	CTD [µM]
		RPF 1	RPF 1.3		RPF 1	RPF 4.5
Mix 6	8000	4000	3077	8000	4000	889
Mix 5	6600	3300	2538	6600	3300	733
Mix 4	5200	2600	2000	5200	2600	578
Mix 3	3800	1900	1462	3800	1900	422
Mix 2	2400	1200	923	2400	1200	267
Mix 1	1000	500	385	1000	500	111

Label	PHP + CTD			PHX + PHP + CTD			
	PHPeq [µM]	PHP [µM]	CTD [µM]	PHXeq [µM]	PHX [µM]	PHP [µM]	CTD [µM]
		RPF 1	RPF 3.6		RPF 1	RPF 1.3	RPF 4.5
Mix 6	7000	3500	972	10,000	3333	2564	741
Mix 5	5800	2900	806	8200	2733	2103	607
Mix 4	4600	2300	639	6400	2133	1641	474
Mix 3	3400	1700	472	4600	1533	1179	341
Mix 2	2200	1100	306	2800	933	718	207
Mix 1	1000	500	139	1000	333	256	74

The final concentration of, e.g. 1000 µM PHX equivalents (PHXeq) in a mixture is composed of 1000 µM/2 = 500 µM PHX and 1000 µM/2/RPF1.3 = 385 µM PHP

Compound concentrations which cover the intermediate part of the single compound concentration–response curve were selected for designing binary and ternary mixtures

The cytotoxicity of the mixtures was investigated in order to exclude unspecific cellular responses due to cytotoxicity. Only the highest concentration level for PHX + PHP and for the ternary mixture was toxic to HepaRG cells (reduction of cell viability to 56 and 47%, respectively) (Fig. S17).

### AOP-wise testing

#### Nuclear receptor activation

**Fig. 3:**
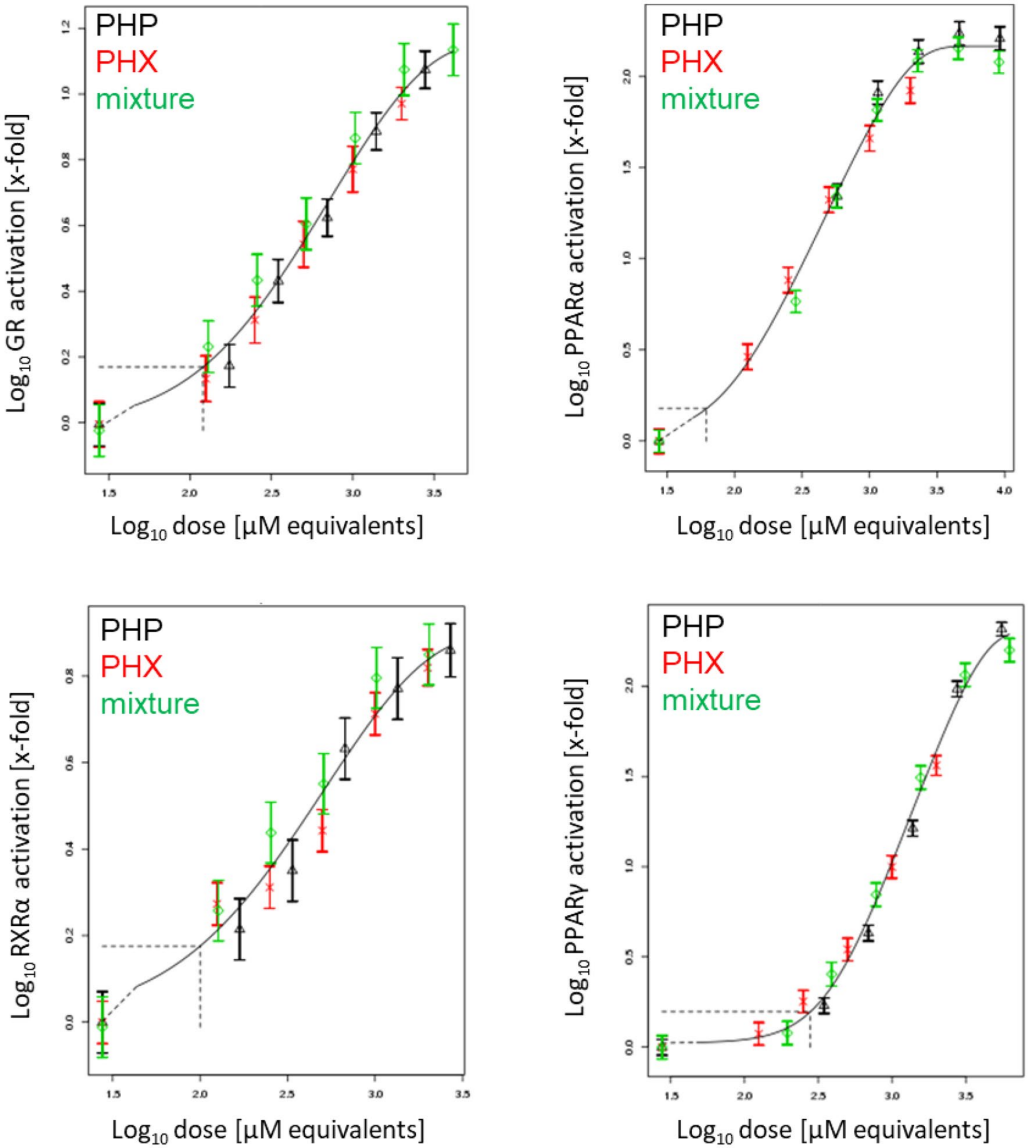
Concentration–response modeling of NR transactivation for binary mixtures of PHX and PHP, based on the data shown in Figs. S18–21. Transfected HepG2 cells were exposed to different concentrations of PHX, PHP, and PHX + PHP. After 24 h cell lysates were assayed for firefly and *Renilla* luciferase activity. Concentration–response modeling was performed using PROAST software and data are presented as means ± SD. The curves represent the four-parameter exponential model; see Eq. 1. The concentration–response data of the mixture (green diamonds) indicate no deviation from the overall concentration–response fit. Thus, dose addition can be assumed (colour figure online)

All mixtures were tested in the reporter gene assays for NRs, which showed the strongest activation in the screening assay, i.e. PPARα and PPARγ. Additionally, GR and RXRα were tested. Please note that only data from nuclear receptors showing unidirectional activation by at least two compounds could be used for subsequent modeling, due to limitations of the chosen BMD-based RPF approach. Therefore, only the mixtures of PHX and PHP were analyzed in that way. Raw data for the combinations including CTD can be found in Figs. S18–S21. Overall, mixtures with CTD (i.e. PHP + CTD, PHX + CTD and PHP + PHX + CTD) showed a concentration-dependent activation of GR, RXRα, PPARα, and PPARγ. Regarding PHX + PHP, data also showed a concentration-dependent activation for all tested receptors. PROAST modeling of the concentration–response data was in agreement with the assumption of dose addition (Fig. 3).

#### PCR-based gene expression analysis

Based on the transcriptional changes proposed in the liver steatosis AOP, and based on the NR activation pattern of PHX, PHP, and CTD, we investigated the expression of *ACOX1, FASN*, *MLXIPL*, *SCD* and *SREBF1*. Additionally, we included other genes related to xenobiotic metabolism, hepatotoxicity and NR activation. A screening of the expression for 56 candidate genes at the EC_50_ dose was initially performed (Supplementary Fig. S22). Genes showing deregulation at the EC_50_ for at least two compounds were then tested at all single compound concentrations. Only genes showing deregulation for at least two individual compounds were further tested for mixture effects.

**Fig. 4:**
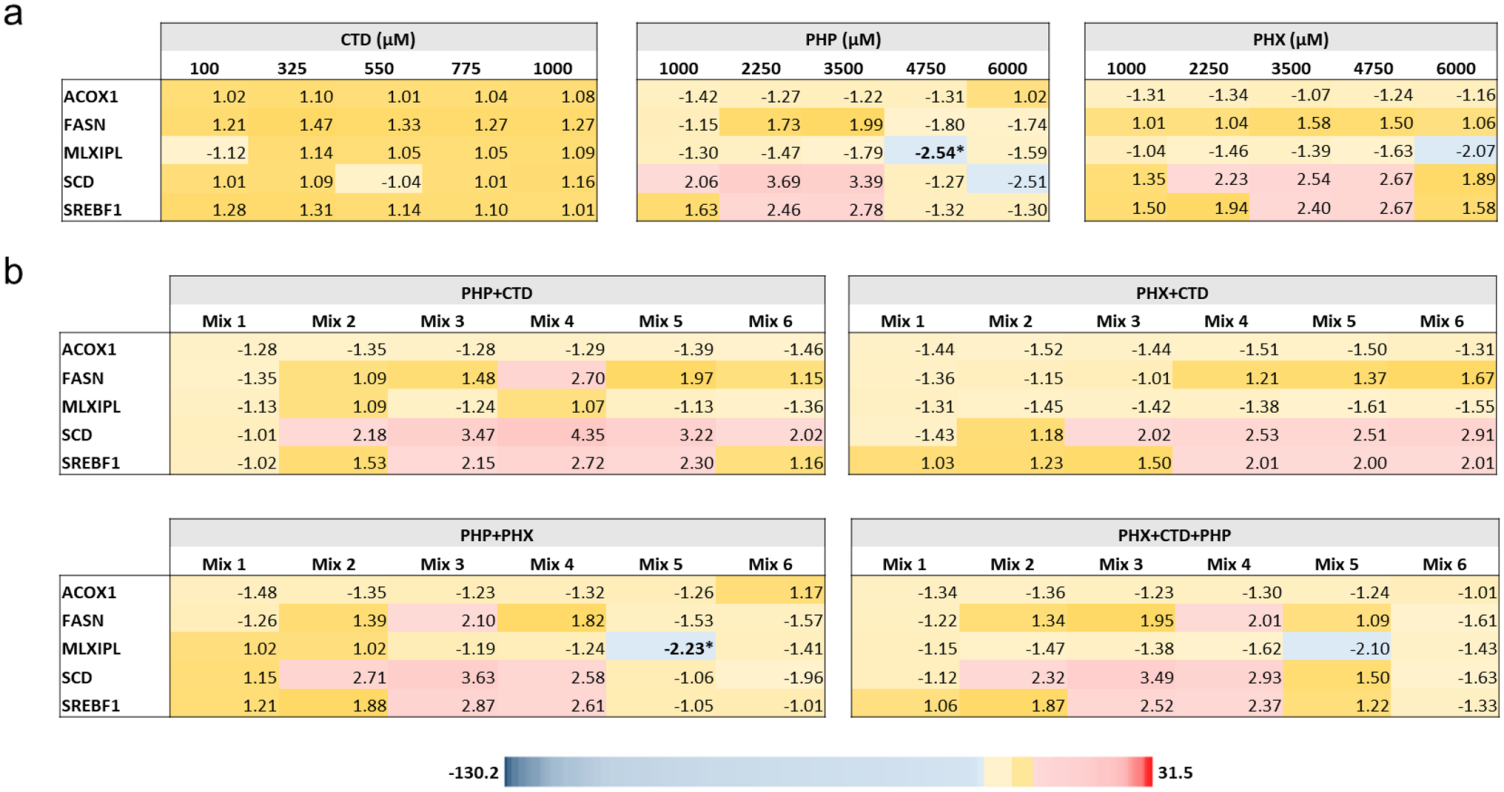
Gene expression analysis linked to liver steatosis, using data obtained with the single compounds (**a**) and their mixtures (**b**). Based on the steatosis AOP, 5 genes were selected for PCR analysis in cells treated for 24 h with different concentrations of PHP, PHX or CTD, or with their binary and ternary mixtures (Table 2). The heat map presents mean fold changes from three independent experiments. Fold changes ≥ 2 and ≤ -2 are highlighted in red and blue, respectively. Statistical significance of differences in expression was based on 2^−ΔCt^ values and was assessed by the nonparametric Kruskal–Wallis test followed by Dunn’s test (*p < 0.05; **p < 0.01; ***p < 0.001 in comparison to control values) (colour figure online)

CTD barely affected the expression of the tested AOP genes. PHP as well as PHX down-regulated the expression of *MLXIPL* only in the middle high and high concentrations, respectively. Moreover, PHX up-regulated the expression of *SCD* and *SREBF1,* without reaching statistical significance. PHP showed a biphasic response with an up-regulation of *SCD* and *SREBF1* at low concentrations followed by a down-regulation at the highest concentrations (Fig. 4a), again without reaching statistical significance. The effects observed in the mixture testing were overall in good agreement with those observed following single compound treatment, i.e. PHP and PHX, which are the molecules exhibiting alterations in gene expression. For instance, the up-regulation of *SCD* and *SREBF1* by PHX was also observed after treatment with PHX + CTD (Fig. 4b). Furthermore, *MLXIPL* was significantly downregulated following treatment with PHP and PHX binary mixture and was only moderately down-regulated with CTD binary mixtures.

**Fig. 5:**
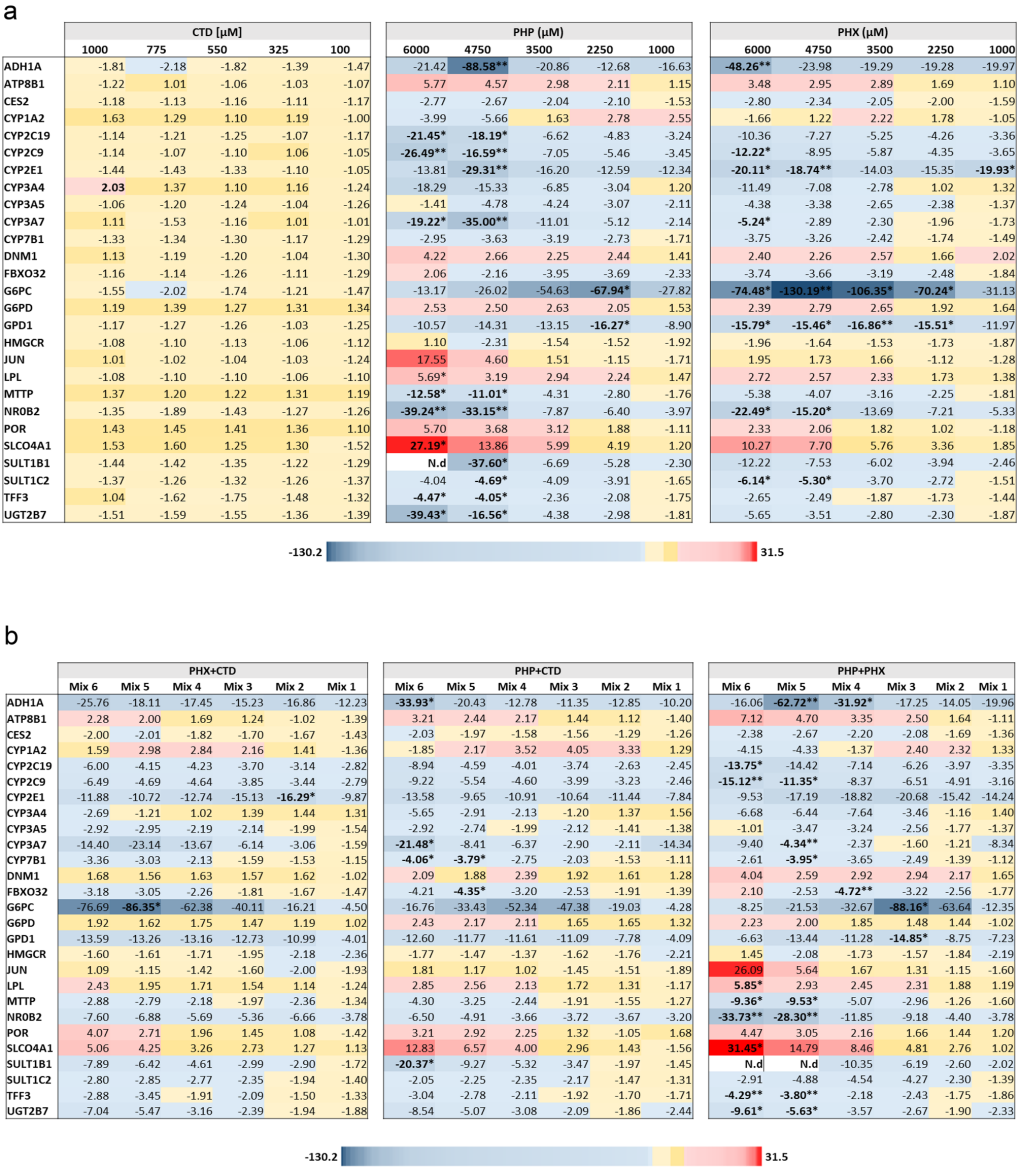

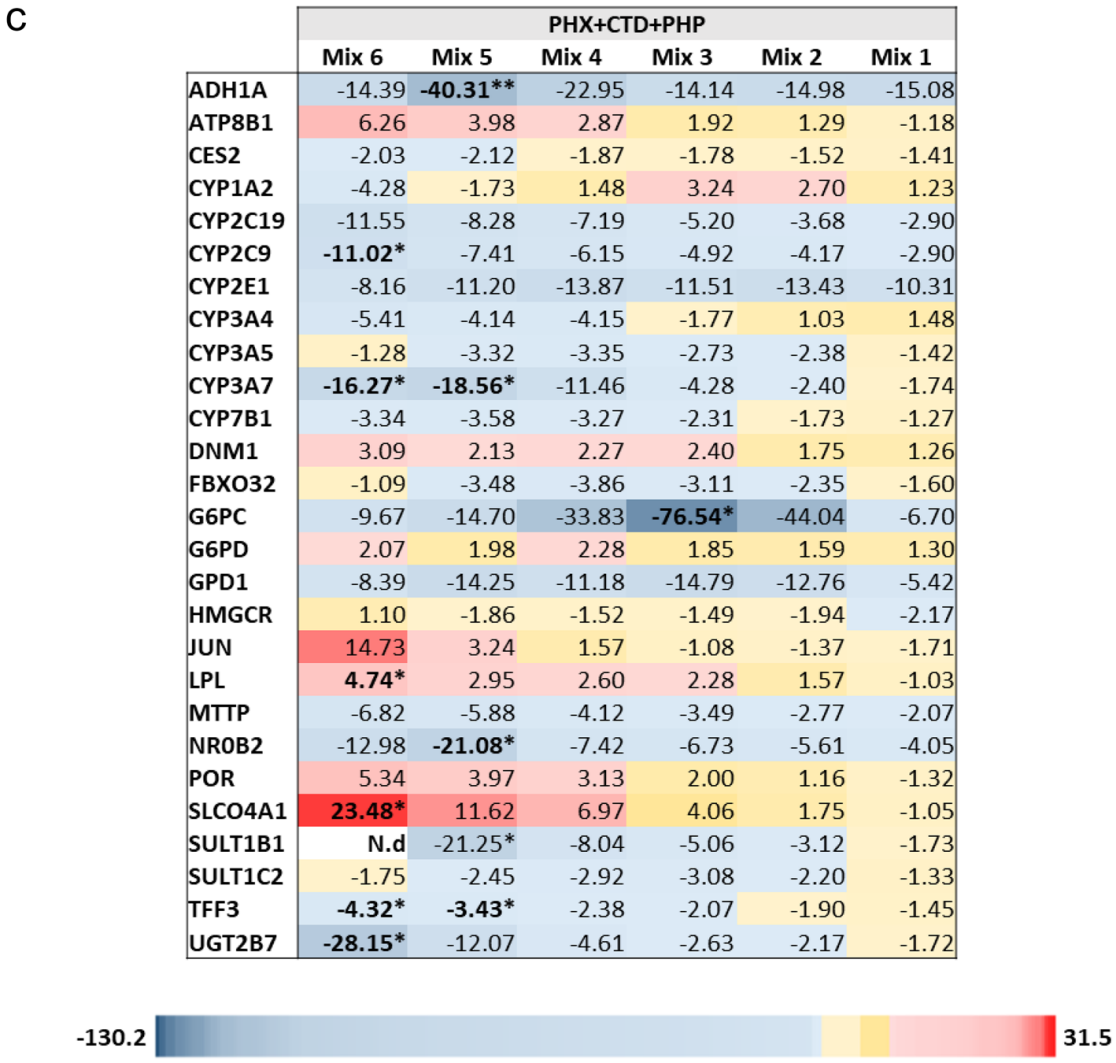
Gene expression analysis linked to xenobiotic metabolism, hepatotoxicity and NR activation, using data obtained with the single compounds PHP, PHX or CTD (**a**) and their mixtures (**b** and **c** for binary and ternary, respectively). Based on the screening (see supplement Fig. S22), 27 genes were selected for PCR analysis in cells treated for 24 h with different concentrations of PHP, PHX or CTD, or with their binary and ternary mixtures (Table 2). The heat map presents mean fold changes of three independent experiments. Fold changes ≥ 2 and ≤ − 2 are highlighted in red and blue, respectively. Statistical significance of differences in expression based on 2^-ΔCt^ values and was assessed by the nonparametric Kruskal–Wallis test followed by Dunn’s test (*p < 0.05; **p < 0.01; *** p < 0.001 in comparison to control values). *N.d* not determined (due to very low expression) (colour figure online)

Based on the initial screening (Supplementary Fig. S22), 27 genes were further selected for PCR analysis in cells treated with different concentrations of PHP, PHX or CTD, or with their binary and ternary mixtures. Again, CTD barely affected the expression of the tested genes. Only a marginal, non-significant up-regulation of *CYP3A4* was observed at the highest tested concentration (Fig. 5a). On the contrary, PHP and PHX deregulated almost all of the 27 additional genes, with both compounds acting in a similar way. Although not all deregulations in the 27 genes were statistically significant, it is worth noting that a global concentration-dependent down-regulation of genes encoding xenobiotic-metabolizing enzymes (XME) was observed, such as phase I CYP genes and phase II SULT and UGT genes. The transporters *SLCO4A1* and *ATP8B1* were highly up-regulated, in a concentration-dependent way (Fig. 5a), although the upregulation reached statistical significance only for *SLCO4A1* in PHP 6000 µM. For the binary mixtures PHX + CTD and PHP + CTD, the gene regulation patterns were similar to those observed with single substance treatment, i.e. PHX or PHP (Fig. 5b). With the ternary mixture, the gene regulation pattern was also similar to what had been observed for treatment with the single substances PHX or PHP (Fig. 5c).

**Fig. 6:**
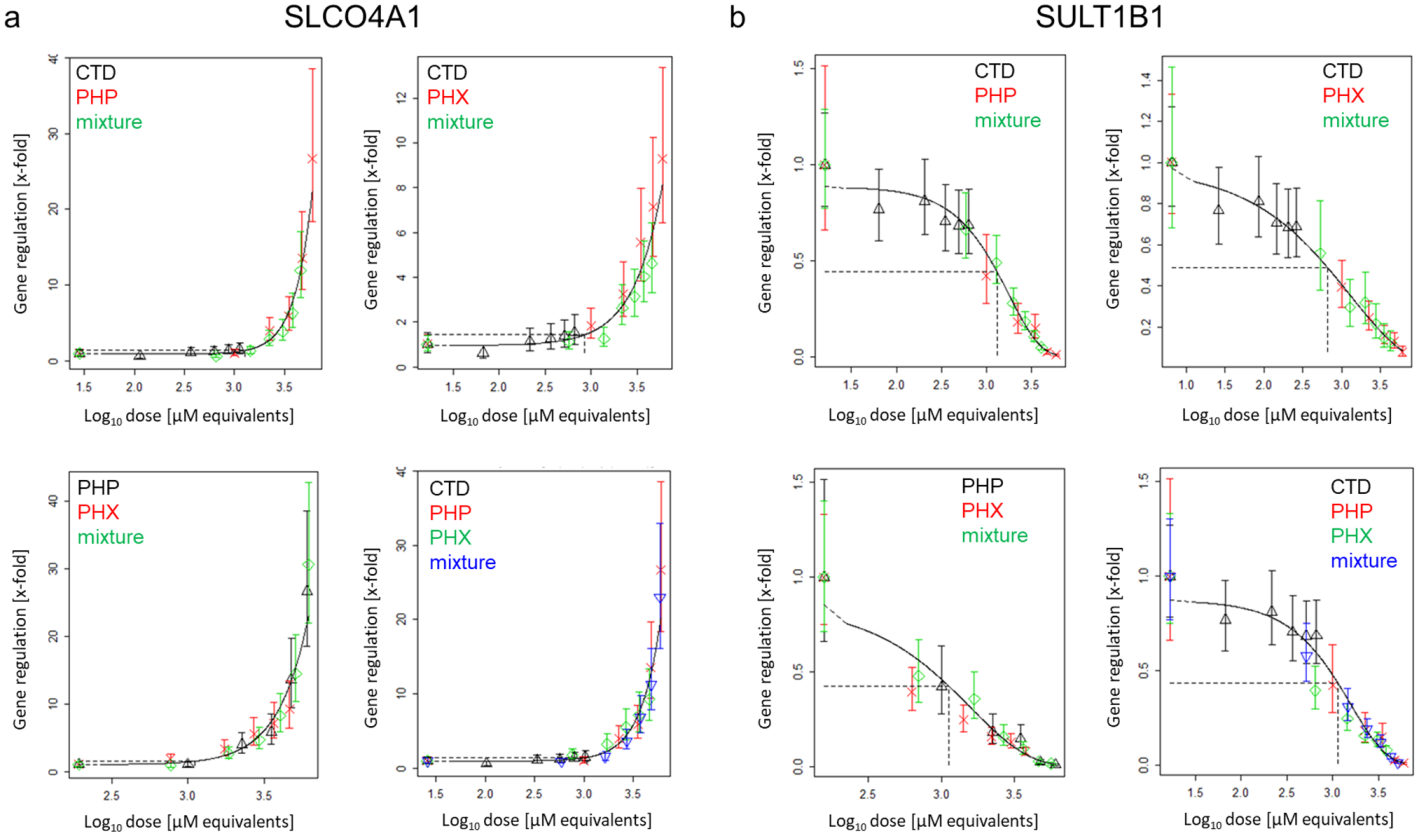
Concentration–response modeling of gene expression data of *SLCO4A1* (**a**) and *SULT1B1* (**b**) for binary and ternary mixtures of PHP, PHX and CTD, based on the data shown in Fig. 4. Differentiated HepaRG cells were exposed to different concentrations of PHP, PHX and CTD, their mixtures, or solvent control (0.5% DMSO) for 24 h. Concentration–response modeling was performed using PROAST software and data are presented as mean ± SEM. The curves represent the four-parameter exponential model; see Eq. 1. The concentration–response data of the mixture (green diamonds in binary mixtures; blue inverse pyramids in ternary mixtures) indicate no deviation from the overall concentration–response fit. Thus, dose addition can be assumed

Concentration–response modeling of representative tested genes is shown in Fig. 6. In general, the mixture data are scattered around the curve fit with no distinct deviation from the overall concentration–response fit, and thus dose addition can be assumed. PROAST modeling for the other genes can be found in supplementary Excel file.

#### Liver triglyceride accumulation

**Fig. 7:**
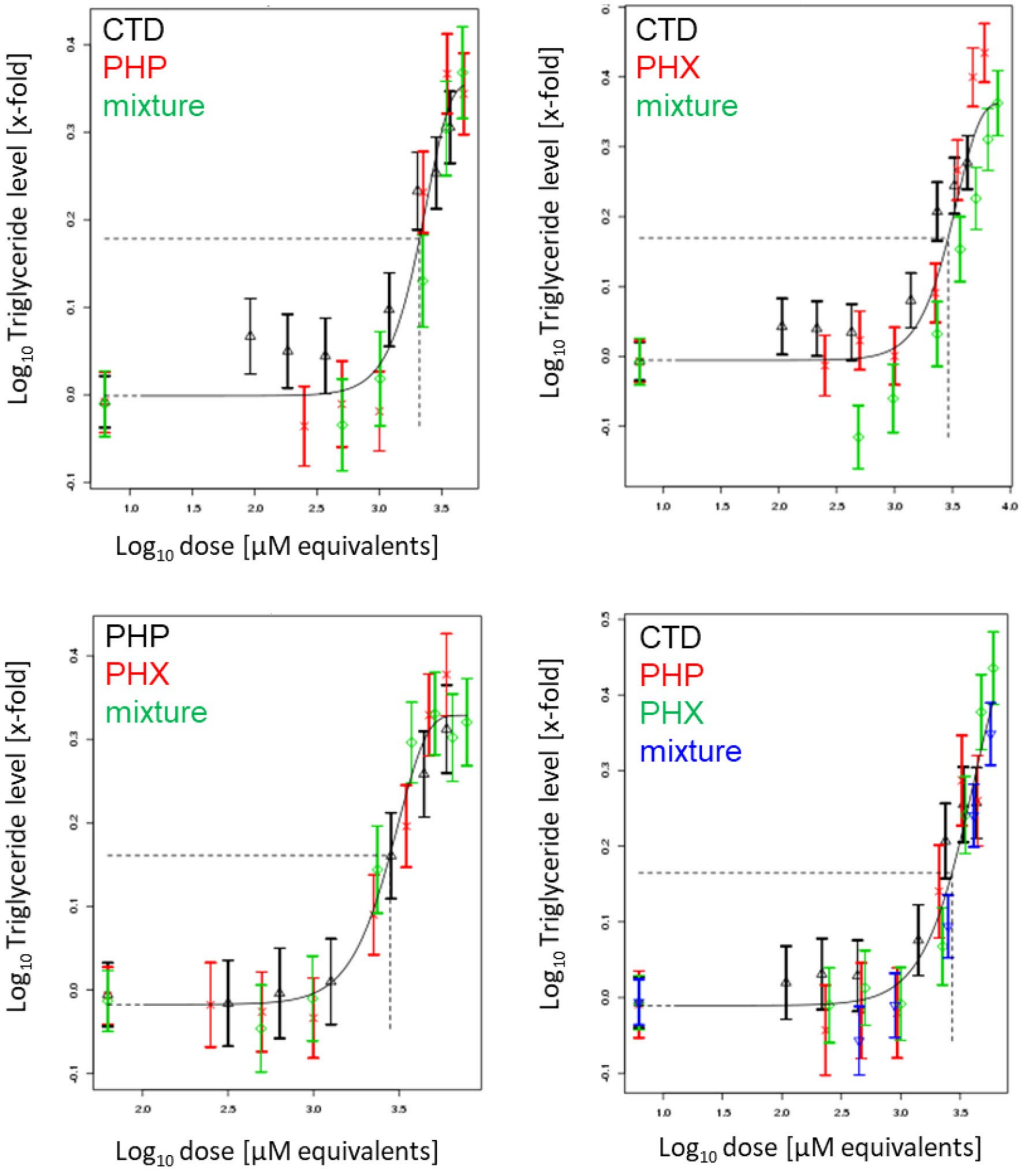
Concentration–response modeling of triglyceride accumulation, as determined by the AdipoRed assay, for binary and ternary mixtures of PHP, PHX and CTD, based on the data shown in Fig. S23. Differentiated HepaRG cells were exposed to different concentrations of PHP, PHX and CTD, their mixtures, or solvent control (0.5% DMSO) for 72 h. Concentration–response modeling was performed using PROAST software and data are presented as means ± SD. The curves represent the four-parameter exponential model; see Eq. 1. The concentration–response data of the mixture (green diamonds in binary mixtures; blue inverse pyramids in ternary mixtures) indicate no deviation from the overall concentration–response fit. Thus, dose addition can be assumed (colour figure online)

Liver triglyceride accumulation was measured using three different assays. In a first step, we performed the AdipoRed assay to measure intracellular lipids following 72 h of treatment with compounds alone or in mixtures. Concentration-dependent increases of intracellular lipids were observed for all test compounds, as well as their mixtures (Fig. S23). PROAST modeling revealed additive behavior for all mixtures (Fig. 7).

In a second step, triglyceride levels were measured using a GC-FID method. Similarly, all test compounds as well as their binary and ternary mixtures induced concentration-dependent increases of triglycerides (44–54 carbon atoms in their fatty acid chains) (Figs. S24 to S29). PROAST modeling showed additivity for all mixtures (see Fig. 8 for representative modeling of C52, and Figs. S30 to S34 for the other chain lengths).

**Fig. 8:**
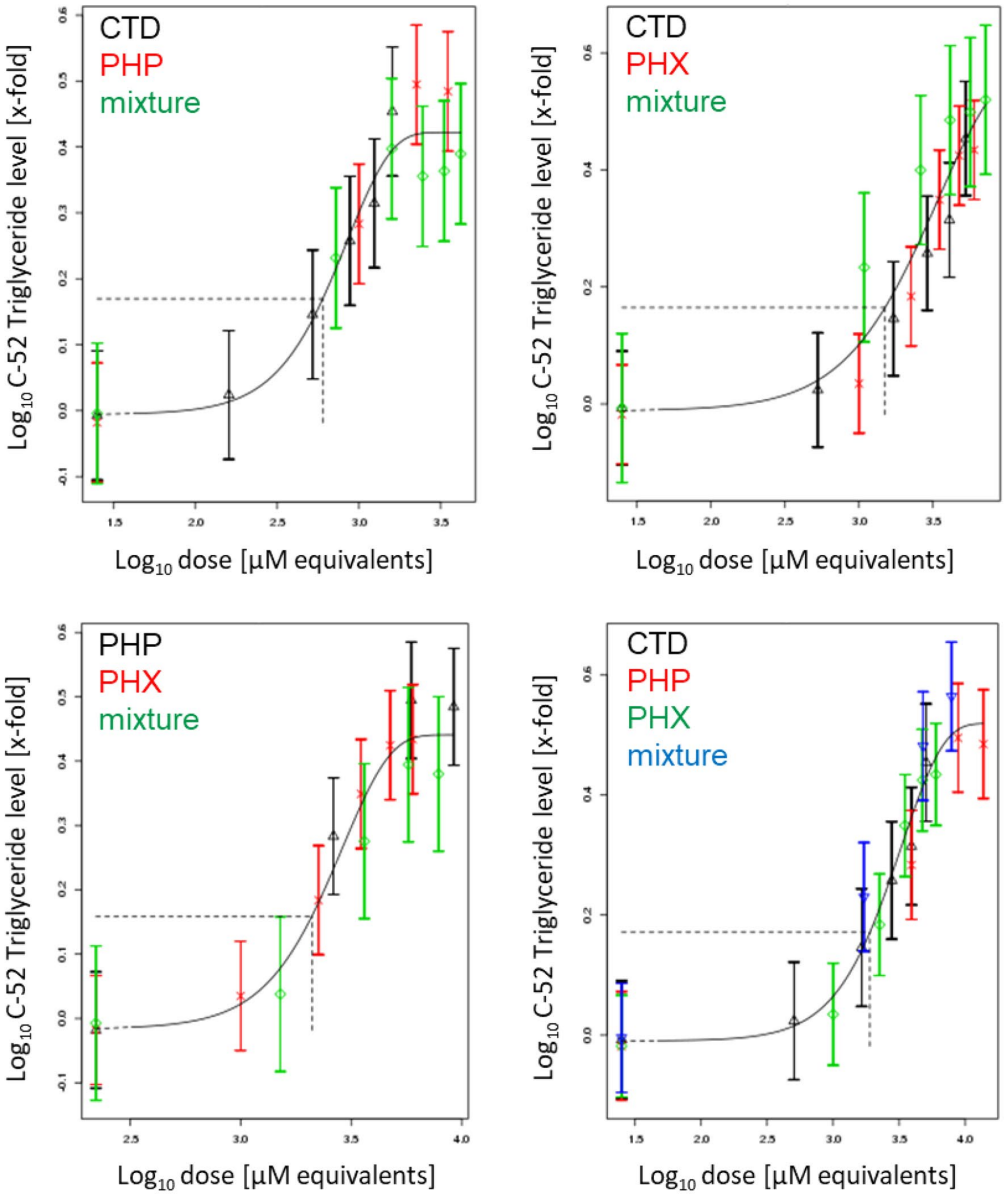
Concentration–response modeling of triglyceride accumulation (C52), as determined by GC-FID for binary and ternary mixtures of PHP, PHX and CTD, based on the data shown in Fig. S28. Differentiated HepaRG cells were exposed to different concentrations of PHP, PHX and CTD, their mixtures, or solvent control (0.5% DMSO) for 72 h. Concentration–response modeling was performed using PROAST software and data are presented as means ± SD. The curves represent the four-parameter exponential model; see Eq. 1. The concentration–response data of the mixture (green diamonds in binary mixtures; blue inverse pyramids in ternary mixtures) indicate no deviation from the overall concentration–response fit. Thus, dose addition can be assumed (colour figure online)

**Fig. 9:**
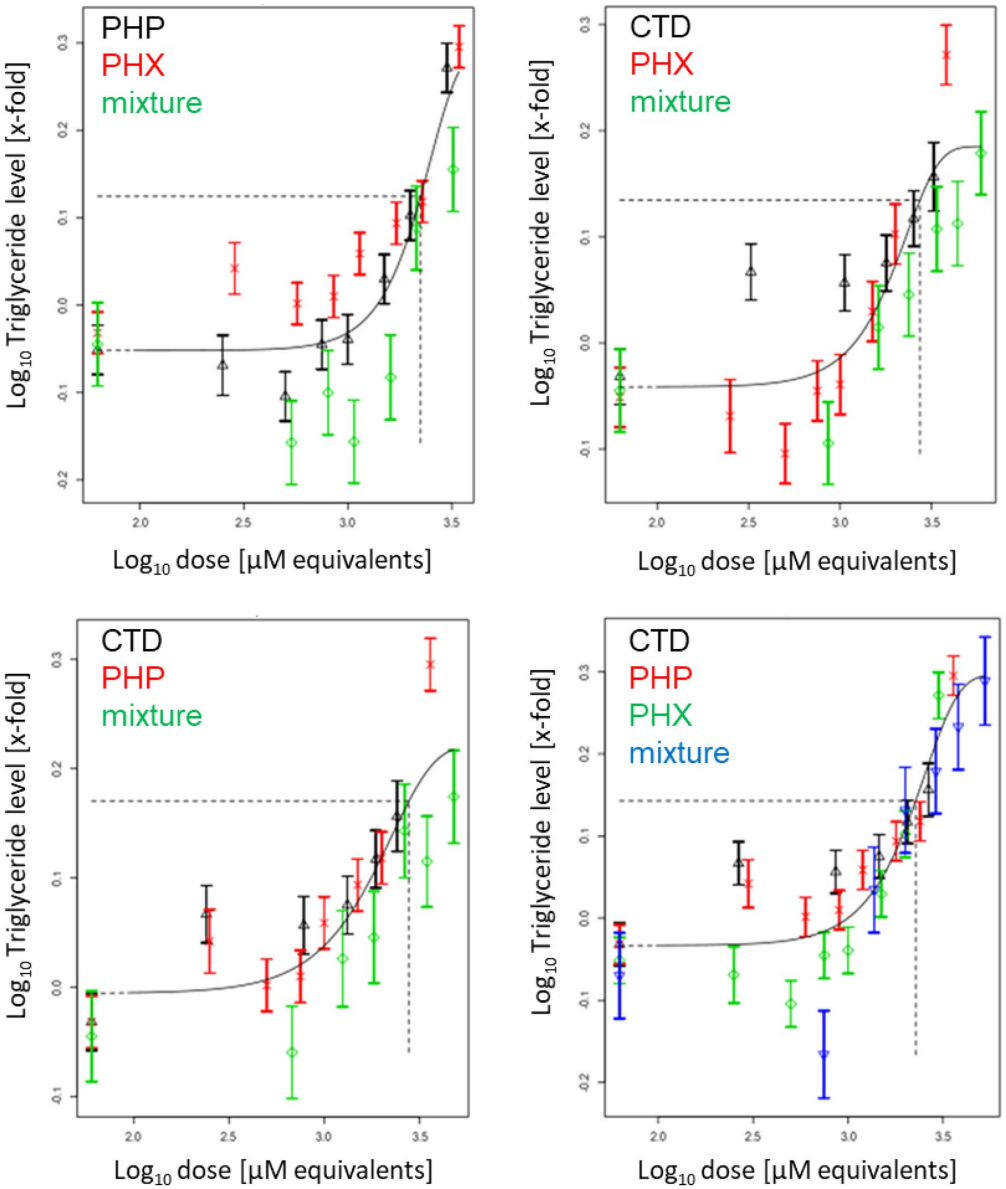
Concentration–response modeling of neutral lipid droplets at the single cell level for binary and ternary mixtures of PHP, PHX and CTD, based on the data shown in Fig. S35. Differentiated HepaRG cells were exposed to different concentrations of PHP, PHX and CTD, their mixtures, or solvent control (0.5% DMSO) for 72 h. Note that concentration levels were modified due to observed cytotoxicity with the concentrations indicated in Table 1 (inter-laboratory variability). Concentration–response modeling was performed using PROAST software and data are presented as means ± SD. The curves represent the four-parameter exponential model; see Eq. 1. The concentration–response data of the mixture (green diamonds in binary mixtures; blue inverse pyramids in ternary mixtures) indicate no deviation from the overall concentration–response fit. Thus, dose addition can be assumed (colour figure online)

Last, a HCI approach was employed to quantify triglycerides at the single-cell level. Consistently with the previous findings, the test compounds and their mixtures induced concentration-dependent increases of triglycerides (Fig. S35). PROAST modeling showed additivity irrespective of the mixture composition (Fig. 9).

#### Evaluation of mixture effects using the ratio of overlap approach

To quantitatively evaluate the mixture effects and to answer whether the assumption of dose addition is valid in the case of the investigated dissimilarly acting compounds, the ratios of overlap were calculated (when possible) for the three different endpoints used to determine triglyceride accumulation. A ratio of overlap above 1 indicates a deviation from dose addition, while a ratio below 1 indicates no deviation from dose addition, i.e. the confidence interval of the second RPF estimate (single compounds + mixture altogether) overlaps with the confidence interval of the single-compounds RPF estimate. As shown in Table 3, all ratios were below 1, indicating no deviation from dose addition. This is in line with the visual observation from Figs. 7, 8, 9.

**Table 3:** Ratios of overlap for equipotent binary and ternary mixtures of PHP, PHX and CTD

Response	Mixture	RPF CI based on the single compounds		RPF CI based on mixtures		Ratio of overlap
		Lowest	Highest	Lowest	Highest	
AdipoRed	PHP + PHX	1.03	1.37	1.06	1.48	0.77
	CTD + PHP	3.37	4.92	3.21	4.30	0.78
	CTD + PHX	3.82	4.61	3.77	4.82	0.82
	PHP + PHX + CTD	PHP: 0.81 CTD: 3.84	PHP: 1.07 CTD: 5.04	PHP: 0.82 CTD: 3.81	PHP: 1.08 CTD: 4.99	PHP: 0.77 CTD: 0.77
High content screening	PHP + PHX	1.05	1.18	1.06	1.23	0.90
	CTD + PHP	2.15	2.67	2.05	2.77	0.77
	CTD + PHX	2.87	3.44	2.83	3.76	0.82
	PHP + PHX + CTD	PHP: 1.05 CTD: 2.45	PHP: 1.18 CTD: 2.81	PHP: 1.10 CTD: 2.42	PHP: 1.33 CTD: 2.93	PHP: 0.93 CTD: 0.86
GC-FID (C44)	PHP + PHX	1.46	4.06	1.48	3.79	0.39
	CTD + PHP	0.76	3.62	1.05	2.75	0.28
	CTD + PHX	3.26	5.02	3.78	5.33	0.75
	PHP + PHX + CTD	PHP: 1.51 CTD: 2.81	PHP: 3.86 CTD: 6.83	PHP: 1.57 CTD: 2.66	PHP: 4.38 CTD: 6.92	PHP: 0.41 CTD: 0.39
GC-FID (C46)	CTD + PHX	4.17	11.10	4.82	7.09	0.59
GC-FID (C48)	CTD + PHX	6.68	14.30	6.05	9.62	0.69
GC-FID (C50)	CTD + PHX	6.25	11.60	6.18	10.10	0.62
GC-FID (C52)	PHP + PHX	2.41	3.86	2.06	3.35	0.72
	CTD + PHP	1.19	2.16	1.1	2.22	0.51
	CTD + PHX	4.05	6.14	3.86	7.08	0.63
	PHP + PHX + CTD	PHP: 2.37 CTD: 4.11	PHP: 3.99 CTD: 6.12	PHP: 2.91 CTD: 4.05	PHP: 5.90 CTD: 6.31	PHP: 0.73 CTD: 0.66
GC-FID (C54)	CTD + PHX	2.26	3.76	1.28	2.94	0.77

## Discussion

Chemical mixtures raise concern at the regulatory level as the components of a mixture may interact to end up with a toxicity higher (i.e. synergism) or lower (i.e. antagonism) than the simple sum of their toxicities taken alone. The difficulty is that such phenomena are extremely difficult to foresee, so that no model can predict ex nihilo if two compounds show higher or lower effect when present in a mixture. Thus, dose addition has been set as the default paradigm for mixtures and different mathematical models have been established to estimate additivity (Bopp et al. 2015; EFSA et al. 2019; EFSA et al. 2013; Kortenkamp et al. 2009). So far, most studies have been performed with mixtures of components having the same MoA. In that case, considering dose addition as the paradigm for mixture effects is intuitive and plausible, as two compounds with same MoA can be perceived as only one substance (with the other compound being theoretically regarded as a dilution of the other). Published data corroborate this statement as dose addition is the most reported mixture effect in the case of similarly acting compounds (Cedergreen 2014). However, for substances with dissimilar MoAs the hypothesis of dose addition as the paradigmatic effect is less intuitive and the amount of published data is too limited to confirm or infirm its validity. The primary goal of this study was to test whether dose addition applies for selected mixtures of dissimilarly acting compounds. For this purpose, a valuable conceptual framework was needed in order to assess mixture effects not only at one single endpoint but also rather on a sequential cascade of events with different endpoints spanning multiple layers of biological significance. The AOP was consequently chosen as a relevant strategy to test compounds with dissimilar MoAs but still sharing a common AO. Thus, we selected compounds with different nuclear receptor activation profiles according to the steatosis AOP and investigated their behavior in mixtures.

Our preliminary screening test showed that VPA and its analogs all induced triglyceride accumulation but with different potencies. In parallel, all compounds activated at least one of the NR predicted as MIE in the liver steatosis AOP. With the exception of 4PEA, there seems to be a correlation between the potencies in the triglyceride accumulation and NR activation, i.e. the compounds with strongest NR activation (e.g. PHP and PHX) are also the ones inducing among the strongest triglyceride accumulation. Interestingly, NR activation by test compounds occurred at lower concentrations than triglyceride accumulation, reflecting a concentration–response relationship between the elements of the AOP where upstream events occur at lower concentrations than subsequent downstream events. This aspect was previously reported by Luckert et al. (2018). Based on the NR activation and triglyceride accumulation data, PHX and PHP were selected for further mixture experiments, along with CTD as test compound for the constitution of dissimilarly acting mixtures.

In our study, PHX and PHP predominantly activated PPARα and PPARγ while CTD antagonized PPARα. Alterations at the mRNA level, as proposed in the AOP, however, were not always consistently observed. For instance, PHP and PHX activated PPARγ and were thus expected to up-regulate *MLXIPL*, *SREBF1* and *SCD* according to the AOP. *SREBF1* and *SCD* were indeed up-regulated but, on the contrary, a down-regulation of *MLXIPL* gene expression was observed. Regarding CTD, no down-regulation of *ACOX1* was observed despite the fact that CTD antagonized PPARα. Our results, therefore, suggest that the current AOP does not completely reflect the biological complexity of chemically induced liver steatosis. This has also been noted previously by Luckert et al. (2018) who reported discrepancies in gene regulation in the steatosis AOP while testing the fungicide cyproconazole. In addition, we reported a global down-regulation of CYP, SULT and UGT genes following treatment with PHX and PHP. This finding might look surprising considering the activation of PXR, CAR or AhR by PHX and PHP, as activation of these receptors would be expected to upregulate XME (Ramadoss et al. 2005; Wang et al. 2012). However, it is noteworthy that processes like inflammation can inhibit the activities of XME (Gu et al. 2006; Tanner et al. 2018). We observed an up-regulation of *JUN*, which codes for a component of the transcription factor AP-1. AP-1 was shown to mediate the release of inflammatory mediators such as IL-8 (Qiao et al. 2016; Wang et al. 2013). Additionally, some inter-connection with NFκB has been documented (Fujioka et al. 2004). Therefore, inflammation-related processes might be triggered in parallel to the triglyceride accumulation and counteract possible XME induction by the activated NRs.

The assessment of mixture effects in the case of dissimilarly acting component mixtures (i.e. CTD with PHX or PHP or both) was partially hampered by the fact that, according to its particular MoA, CTD barely induced effects regarding NR activation and AOP-related gene expression. In such situation, the RPF-based approach does not allow to perform quantitative evaluation of the data. Nonetheless, it has to be noted that the response profiles of the mixtures were always similar to the ones of PHX or PHP alone, considering the compound potencies and their effective concentrations in the mixtures. Therefore, it is likely that no remarkable deviation from additivity occurs for these endpoints. Quantitative assessment, however, was possible with the aforementioned dissimilarly acting mixtures at the level of triglyceride accumulation, showing the most direct relevance for the AO of steatosis. We reported a strong consistency within the results obtained with three different methodologies. In addition, the behavior of all investigated mixtures followed the assumption of dose addition irrespective of the MoA of the mixture components. This shows that dose addition can also apply to mixtures of compounds with dissimilar molecular MoA. In fact, the question of similarity/dissimilarity of the MoA at the molecular level might not be the key element in the behavior of components within a mixture. Other studies have shown that components with dissimilar MoA also followed dose addition when present in mixtures (Staal et al. 2018; Zoupa et al. 2020). Nonetheless, the very limited number of mixtures investigated so far does not allow to conclude that dose addition is a valid assumption for all mixtures with different MoA. Moreover, it is not always easy to define similarity or dissimilarity of the MoA in case of complex biological processes such as the steatosis AOP and partially overlapping molecular targets of many test compounds. It should also be noted that dose addition may apply at the common endpoint level (i.e. the AO), while it does not necessarily apply as well in all the intermediate KEs of an AOP network. Since the time of exposure is an important aspect in the toxicological behavior of chemicals (whether alone or in mixtures), assessment of scenarios of repeated-dose effects would reflect more precisely the human exposure. Different studies have reported the successful use of models like HepaRG cells or primary human hepatocyte spheroids for repeated-exposure induced steatosis (Antherieu et al. 2011; Bell et al. 2016; van Breda et al. 2018). Thus, future research involving advanced models and chronic exposure treatment will increase our understanding of toxicological outcomes of chemicals.

## Conclusion

In this study, we aimed to investigate whether the assumption of dose addition can also apply to mixtures composed of dissimilarly acting compounds. For this purpose, we employed the BMD-based RPF approach within an AOP-wise testing strategy. Dose additivity for binary and ternary mixtures of the three test compounds was observed for the KE of triglyceride accumulation. Therefore, our data show that compounds with dissimilar molecular MoA can still follow dose addition at apical endpoints when being present in mixtures.

## Supplementary Information

Below is the link to the electronic supplementary material.

Supplementary file1 (DOCX 2959 kb)

Supplementary file2 (XLSX 539 kb)

## Abbreviations

2EHA: 2-Ethylhexanoic acid
4eVPA: (±)-2-Propyl-4-pentenoic acid
4PEA: 4-Pentenoic acid
AhR: Aryl hydrocarbon receptor
AO: Adverse outcome
AOP: Adverse outcome pathway
AUC: Area under the curve
BMD: Benchmark dose
BMDL: Lower confidence limit of the benchmark dose
BMDU: Upper confidence limit of the benchmark dose
BMR: Benchmark response
CAR: Constitutive androstane receptor
CTD: Clothianidin
CYP: Cytochrome P450
DMEM: Dulbecco’s modified Eagle’s medium
DMSO: Dimethyl sulfoxide
DPBS: Dulbecco’s phosphate-buffered saline
FXR: Farnesoid X receptor
GC-FID: Gas chromatography with flame-ionization detection
GR: Glucocorticoid receptor
HCI: High-content cell imaging
KE: Key event
LXR: Liver X receptor
MIE: Molecular initiating event
MoA: Mode of action
NR: Nuclear receptor
PBS: Phosphate-buffered saline
PHP: 2-Propylheptanoic acid
PHX: 2-Propylhexanoic acid
PPAR: Peroxisome proliferator-activated receptor
PXR: Pregnane X receptor
RAR: Retinoic acid receptor
RPF: Relative potency factor
RPFL: Lower bound of the RPF confidence interval
RPFU: Upper bound of the RPF confidence interval
RXR: Retinoid X receptor
VPA: Valproic acid
XME: Xenobiotic-metabolizing enzymes
