# Supplementary material for: Investigating the in vitro steatotic mixture effects of similarly and dissimilarly acting test compounds using an adverse outcome pathway-based approach
Source: Arch Toxicol. 2021 Nov 15;96(1):211–29. doi: 10.1007/s00204-021-03182-1 (PMC8748329; doi:10.1007/s00204-021-03182-1)
Supplement: Supplementary file 1 — Supplementary file1 (DOCX 2959 kb) [file 204_2021_3182_MOESM1_ESM.docx]

**Supporting Information**

**Investigating the *in vitro* steatotic mixture effects of similarly and dissimilarly-acting test compounds using an adverse outcome pathway-based approach**

**Jimmy Alarcan^1^, Georges de Sousa^2^, Efrosini S. Katsanou^3^, Anastasia Spyropoulou^3^, Petros Batakis^3^, Kyriaki Machera^3^, Roger Rahmani^2^, Alfonso Lampen^1^, Albert Braeuning^1,#^, Dajana Lichtenstein^1^**

^1^German Federal Institute for Risk Assessment, Dept. Food Safety, Berlin, Germany

^2^Institut Sophia Agrobiotech, Université Côte d'Azur-INRAE-CNRS, F-06903 Sophia Antipolis, France

^3^Benaki Phytopathological Institute, Athens, Greece

^#^Corresponding author: Albert Braeuning, German Federal Institute for Risk Assessment, Dept. Food Safety, Max-Dohrn-Str. 8-10, 10589 Berlin, Germany, Tel. +49-(0)30-18412-25100, Fax +49-(0)30-18412-99099, E-mail: Albert.Braeuning@bfr.bund.de

**Table S1** Plasmids, cell lines and positive controls used for the different reporter gene assays*^a^*

| **Reporter gene assay** | **Plasmid***^b^* | **Plasmid amount** | **Cell line** | **Positive control** |
| --- | --- | --- | --- | --- |
| AhR | p3xDREC | 80 ng/well | HepG2 | 5 µM 3-Methylcholanthrene |
| CAR | pGAL4/DBD-hCAR/LBD(+3aa) | 40 ng/well | HepG2 | 10 µM CITCO |
|  | pGAL4-(UAS)5-TK-Luc | 40 ng/well |  |  |
| FXR | pGAL4-(UAS)5-TK-LUC | 40 ng/well | HepG2 | 10 µM GW 4064 |
|  | pGAL4-FXR-LBD | 40 ng/well |  |  |
| GR | pGAL4-(UAS)5-TK-LUC | 40 ng/well | HepG2 | 10 µM Dexamethasone |
|  | pGAL4-GR-LBD | 40 ng/well |  |  |
| LXRα | pGAL4-(UAS)5-TK-LUC | 40 ng/well | HepG2 | 10 µM GW 3965 |
|  | pGAL4-LXRα-LBD | 40 ng/well |  |  |
| PPARα | pGAL4-(UAS)5-TK-LUC | 40 ng/well | HepG2 | 1 µM GW 7647 |
|  | pGAL4-hPPARα-LBD | 40 ng/well |  |  |
| PPARγ | pGAL4-(UAS)5-TK-LUC | 40 ng/well | HepG2 | 10 µM Troglitazon |
|  | pGAL4-hPPARγ-LBD | 40 ng/well |  |  |
| PPARδ | pGAL4-(UAS)5-TK-LUC | 40 ng/well | HepG2 | 1 µM GW 501516 |
|  | pGAL4-hPPARδ-LBD | 40 ng/well |  |  |
| PXR | pGAL4-(UAS)5-TK-LUC | 40 ng/well | HepG2 | 10 µM SR12813 |
|  | pGAL4-PXR-LBD | 40 ng/well |  |  |
| RARα | pGAL4-(UAS)5-TK-LUC | 40 ng/well | HepG2 | 100 nM AM580 |
|  | pCMX-GAL4-hRARα | 40 ng/well |  |  |
| RXRα | pGAL4-(UAS)5-TK-LUC | 40 ng/well | HepG2 | 100 nM CD 2608 |
|  | pCMX-GAL4-hRXRα | 40 ng/well |  |  |

*^a^*Transfected cells were exposed to respective positive controls and test compounds for 24 h.

*^b^*For normalization purposes cells were always additionally transfected with 1 ng/well of *Renilla* luciferase expression plasmid pcDNA3-Rluc.

**Table S2** Oligonucleotide sequences of gene primers used in real-time PCR and amplicon sizes

| **Genes Name** | **Gene accession no.** | **Sequences** | **Amplicon size (bp)** |
| --- | --- | --- | --- |
| *ACACA* | >NM_198834.3  >NM_198839.3  >NM_198836.3 >NM_198837.2  >NM_198838.2 | Fw 5’-->3’: GCAAATCCGCAGCTTGGTC Rv 5’-->3’: CCGTATGACTTCTGCTCGCT | 103 |
| *ACOX1* | >NM_004035.7  >NM_007292.6  >NM_001185039.2 | Fw 5’-->3’: TGACACTTGGCTCTGTGCTT Rv 5’-->3’: TCGTGGACCTCTGCTTTGTT | 100 |
| *ACTB* | >NM_001101.5 | Fw 5’-->3’: TTCCTTCCTGGGCATGGAGT Rv 5’-->3’: CAATGCCAGGGTACATGGTG | 137 |
| *ADH1A* | >NM_000667.4 | Fw 5’-->3’: GACCTGCTTCACTCTGGGAAAA Rv 5’-->3’: AAGACTGCCACAAGGGAAAACA | 81 |
| *ADK* | >NM_001123.4  >NM_006721.4  >NM_001202449.2  >NM_001202450.2 >NM_001369124.1 | Fw 5’-->3’: AGGCAGCGAATCGTGATCTT Rv 5’-->3’: CCAGTTGAGACAGAAAACCTCC | 160 |
| *ALDH1A1* | >NM_000689.5 | Fw 5’-->3’: AGCCATAACAATCTCCTCTGCT Rv 5’-->3’: CCGTACTCTCCCAGTTCTCTTC | 129 |
| *ALDH2* | >NM_000690.4  >NM_001204889.2 | Fw 5’-->3’: TGGGAGAGCCAACAATTCCAC Rv 5’-->3’: ACATCATAGCAGTTGACCCACA | 123 |
| *ATP8B1* | >NM_001374385.1  >NM_005603.6  >NM_001374386.1 | Fw 5’-->3’: GCTCGACCAGGATGTGAGTG Rv 5’-->3’: TCGATGTTAGGACCCCATGC | 128 |
| *B2M* | >NM_004048.4 | Fw 5’-->3’: TGTCTTTCAGCAAGGACTGGT Rv 5’-->3’: TGCTTACATGTCTCGATCCCAC | 143 |
| *CES2* | >NM_003869.6  >NM_198061.3  >NM_001365406.1  >NM_001365405.1  >NM_001365407.1  >NM_001365408.1 | Fw 5’-->3’: CCTGTCCCTAGCATTGTTGGT Rv 5’-->3’: ATGTAGGAGGCAACATCAGCA | 154 |
| *COMT* | >NM_000754.3  >NM_001135161.1  >NM_001135162.1  >NM_007310.2  >NM_001362828.1 | Fw 5’-->3’: TCTCAACTGCCATTCCCCTG Rv 5’-->3’: TAGCGTTAGCGTCCGTCAAG | 77 |
| *CYP1A2* | >NM_000761.5 | Fw 5’-->3’: CACAGCACAACAAGGGACAC Rv 5’-->3’: ACAGCTCTGGGTCATGGTT | 100 |
| *CYP1B1* | >NM_000104.4 | Fw 5’-->3’: CGCGGCTGGATTTGGAGA Rv 5’-->3’: TCCAATTCTGCCTGCACTCG | 145 |
| *CYP2B6* | >NM_000767.5 | Fw 5’-->3’: GCCATACACAGAGGCAGTCA Rv 5’-->3’: TCTGTGTCCTTGGGGATGATG | 123 |
| *CYP2C19* | >NM_000769.4 | Fw 5’-->3’: ACTTGGAGCTGGGACAGAGA Rv 5’-->3’: GCTCCGGTTTCTGCCAATGA | 127 |
| *CYP2C9* | >NM_000771.4 | Fw 5’-->3’: CATGCAAGACAGGAGCCACA Rv 5’-->3’: TTGTGCCCTTGGGAATGAGA | 143 |
| *CYP2D6* | >NM_000106.6  >NM_001025161.3 | Fw 5’-->3’: CCTCTTCTTCACCTCCCTGC Rv 5’-->3’: GGATGGGCTCACCAGGAAAG | 103 |
| *CYP2E1* | >NM_000773.4 | Fw 5’-->3’: TCATCCCCAAGGGCACAGTC Rv 5’-->3’: TCATTCAGGAAGTGTTCTGGCTT | 106 |
| *CYP3A4* | >NM_017460.6  >NM_001202855.3 | Fw 5’-->3’: AGTGTGGGGCTTTTATGATGGT Rv 5’-->3’: CCTCCGGTTTGTGAAGACAGAA | 109 |
| *CYP3A5* | >NM_000777.5  >NM_001291829.2  >NM_001291830.2 | Fw 5’-->3’: CCCAAAGGGTCAATGGTGGT Rv 5’-->3’: TACTGAACCTTTCAGGGCGGAA | 97 |
| *CYP3A7* | >NM_000765.5 | Fw 5’-->3’: AGAAACACAGATCCCCCTGA Rv 5’-->3’: CAGGCTCCACTTACGGTCTC | 105 |
| *CYP7B1* | >NM_004820.5  >NM_001324112.2 | Fw 5’-->3’: TTCCCATCCACCTCACCAGA Rv 5’-->3’: CCTTTCGCACACAGTAGTCC | 159 |
| *DNM1* | >NM_004408.4  >NM_001005336.3  >NM_001288737.2  >NM_001288738.2  >NM_001288739.2  >NM_001374269.1 | Fw 5’-->3’: CGCACTAAGGAGCAGGTCA Rv 5’-->3’: TGAAGTCCTCATGGTTGGTGT | 73 |
| *ENO1* | >NM_001428.5  >NM_001201483.3  >NM_001353346.2 | Fw 5’-->3’: CAACCCAAAGAGGATCGCCA Rv 5’-->3’: CTTGCACGCCTGAAGAGACT | 106 |
| *FAS* | >NM_000043.6 >NM_152871.4  >NM_152872.4  >NM_001320619.2 | Fw 5’-->3’: TGTCCTCCAGGTGAAAGGAAAG Rv 5’-->3’: GGGCTTTGTCTGTGTACTCC | 100 |
| *FASN* | >NM_004104.5 | Fw 5’-->3’: GTCTTGAACTCCTTGGCGGA Rv 5’-->3’: AGGAAGATAGCCATGCCGAG | 128 |
| *FBXO32* | >NM_058229.3  >NM_148177.2  >NM_001242463.1 | Fw 5’-->3’: TGGCAAAGCTGAATGCGAAC Rv 5’-->3’: TTCCAGCCACACCAAAAGGT | 92 |
| *GAPDH* | >NM_002046.7  >NM_001256799.3 >NM_001289745.3  >NM_001289746.2 >NM_001357943.2 | Fw 5’-->3’: CGACCACTTTGTCAAGCTCA Rv 5’-->3’: GGGTCTTACTCCTTGGAGGC | 102 |
| *G6PC* | >NM_000151.4  >NM_001270397.2 | Fw 5’-->3’: TCCTGTCAGGCATTGCTGTTG Rv 5’-->3’: TACACCCAGTCCCTTGAGCA | 143 |
| *G6PD* | >NM_001360016.2  >NM_000402.4  >NM_001042351.3 | Fw 5’-->3’: AGAACGTGAAGCTCCCTGAC Rv 5’-->3’: TGAAAATACGCCAGGCCTCA | 111 |
| *GPD1* | >NM_005276.4  >NM_001257199.2 | Fw 5’-->3’: GGAGATGATAGCCTTCGCCA Rv 5’-->3’: GCTCAATGGACTTTCCTGTACGC | 158 |
| *HAAO* | >NM_012205.3 | Fw 5’-->3’: GAATGTGGACGTGTGGCTGT Rv 5’-->3’: CCAGGCATACGAGGTCCCA | 121 |
| *HADHB* | >NM_000183.2  >NM_001281512.1  >NM_001281513.1 | Fw 5’-->3’: AGGCAGGATTGACCATGAATGA Rv 5’-->3’: TGCCAAAATCTGACCCGAGA | 71 |
| *HMGCR* | >NM_000859.2  >NM_001130996.1  >NM_001364187.1 | Fw 5’-->3’: TGTTTGCAGATGCTAGGTGTTC Rv 5’-->3’: CACACAATTCGGGCAAGCTG | 80 |
| *IL6* | >NM_000600.5  >NM_001318095.2  >NM_001371096.1 | Fw 5’-->3’: TAGTGAGGAACAAGCCAGAGC Rv 5’-->3’: TGGGTCAGGGGTGGTTATTG | 103 |
| *INSIG1* | >NM_005542.6 >NM_198337.4  >NM_001346592.2 >NM_001346593.2  >NM_001346594.2 | Fw 5’-->3’: ATTCGTTCTTGGCTCCCTTGT Rv 5’-->3’: TCAGGAACACCCATAGCTAACTG | 86 |
| *JUN* | >NM_002228.4 | Fw 5’-->3’: GACAAGTTGCGACGGAGAGA Rv 5’-->3’: CAAGTCCTTCCCACTCGTGC | 116 |
| *KHK* | >NM_000221.3  >NM_006488.3 | Fw 5’-->3’: GAGGAAAGGGGCTGTGCTT Rv 5’-->3’: AAAGCATCCGAGTGGAGCAA | 90 |
| *LMNA* | >NM_170707.3  >NM_170708.3 | Fw 5’-->3’: GGTCACCCGCTCCTACCTC Rv 5’-->3’: TGGCAGGTCCCAGATTACATGA | 87 |
| *LPL* | >NM_000237.3 | Fw 5’-->3’: CAGGCTGAAACTGGGCGAAT Rv 5’-->3’: TGAAACACCCCAAACACTGG | 111 |
| *MAPK8* | >NM_001323302.2  >NM_139049.4  >NM_139046.4  >NM_001278547.2  >NM_001278548.1 >NM_001323320.2  >NM_001323321.2  >NM_001323322.2 >NM_001323323.2  >NM_001323324.2  >NM_001323325.2  >NM_001323326.2  >NM_001323327.2  >NM_001323328.2  >NM_001323329.2  >NM_001323330.2  >NM_001323331.2 | Fw 5’-->3’: CAGAAGCTCCACCACCAAAGA Rv 5’-->3’:TGCCCCCGTATAACTCCATTC | 136 |
| *MLXIPL* | >NM_032951.3  >NM_032952.3  >NM_032953.3  >NM_032954.3 | Fw 5’-->3’: AGCGTTTTGACCAGATGCGA Rv 5’-->3’: TGCTGAACACCCAGAACTTCC | 84 |
| *MSMO1* | >NM_006745.4  >NM_001017369.2 | Fw 5’-->3’: TCATCATGAGTTTCAGGCTCCA Rv 5’-->3’: AATGGTCACCCATGCCCAAA | 142 |
| *MTTP* | >NM_000253.4  >NM_001300785.2  >NM_001386140.1 | Fw 5’-->3’: TCCCCGTTCGGCATCTACTT Rv 5’-->3’: TAACCACCTGGCTACCGTGA | 125 |
| *NQO1* | >NM_000903.3  >NM_001025433.2  >NM_001025434.2  >NM_001286137.2 | Fw 5’-->3’: CTCACCGAGAGCCTAGTTCC Rv 5’-->3’: TCAGTGCTCTTCTGCCGAC | 104 |
| *NR0B2* | >NM_021969.3 | Fw 5’-->3’: TGGCTTCAATGCTGTCTGGA Rv 5’-->3’: CTGGCACATCGGGGTTGAA | 100 |
| *PCCA* | >NM_000282.3  >NM_001127692.2  >NM_001178004.1  >NM_001352605.1  >NM_001352606.1  >NM_001352607.1  >NM_001352608.1  >NM_001352610.1  >NM_001352611.1  >NM_001352612.1 | Fw 5’-->3’: AACTGGCACGGTGAAATCTGT Rv 5’-->3’: ATCCTTCATTCCAGCTCCACG | 84 |
| *PDK4* | >NM_002612.4 | Fw 5’-->3’: TCAGACAGAGGAGGTGGTGTT Rv 5’-->3’: AAACCAGCCAAAGGAGCATTCC | 116 |
| *PNPLA3* | >NM_025225.3 | Fw 5’-->3’: ACGATGTCCTGTGGTTGCAG Rv 5’-->3’: TCACTGGCATTTGGGACCTG | 93 |
| *POR* | >NM_000941.2 | Fw 5’-->3’: CTACGTCTGTGGGGATGCAC Rv 5’-->3’: TTGATGTAGTCCACCGCCTG | 111 |
| *PPARA* | >NM_001001928.3  >NM_005036.5  >NM_001362872.1  >NM_001362873.1 | Fw 5’-->3’: CTGTGGAGATCGTCCTGGC Rv 5’-->3’: CAGGTGGAGTCTGAGCACAT | 85 |
| *PPRGC1A* | >NM_001330751.2  >NM_013261.5  >NM_001330752.2  >NM_001330753.2  >NM_001354825.2  >NM_001354826.2 | Fw 5’-->3’: TCTGCGGGATGATGGAGACA Rv 5’-->3’: TTGCTTGCGTCCACAAAAGT | 139 |
| *RGCC* | >NM_014059.3 | Fw 5’-->3’: CACTGTCACTCCTCAGAAAGCTAA Rv 5’-->3’: TTAGCAGGTCCTCGGAACTTT | 156 |
| *SCD1* | >NM_005063.5 | Fw 5’-->3’: TTTCACTTGGAGCTGTGGGTGAG Rv 5’-->3’: AAGTTGATGTGCCAGCGGT | 94 |
| *SLCO4A1* | >NM_016354.4 | Fw 5’-->3’: GGCAACTCTACGATGTGTCCG Rv 5’-->3’: TTGTCGATCACCCAGCCGAA | 120 |
| *SREBF1* | >NM_001005291.3  >NM_004176.5  >NM_001321096.3  >NM_001388385.1  >NM_001388386.1  >NM_001388388.1  >NM_001388389.1  >NM_001388390.1  >NM_001388391.1  >NM_001388392.1  >NM_001388394.1 | Fw 5’-->3’: GATGCTGGCTCACCTTTCCA Rv 5’-->3’: GCTTTGCCTTGCTGTCCTCA | 121 |
| *STBD1* | >NM_003943.4 | Fw 5’-->3’: GAGAAAGACGCCCCTCTTGG Rv 5’-->3’: TGAAGATGCTCTGGTTTGGTG | 116 |
| *SULT1B1* | >NM_014465.4 | Fw 5’-->3’: TATGCGTAAAGGGACGGCTG Rv 5’-->3’: TGTGCGGAATTGAAGTGCAG | 115 |
| *SULT1C2* | >NM_001056.4  >NM_176825.3 | Fw 5’-->3’: GAAGGTGGATGAAACAGTGCT Rv 5’-->3’: CCCCCACAGTTCCTTTTCTCA | 149 |
| *SYT1* | >NM_005639.2  >NM_001135805.1  >NM_001135806.1  >NM_001291901.1 | Fw 5’-->3’: ACTGTTGTCATTCTGGAGGCA Rv 5’-->3’: TCACATAAGGATCGGATAAGCCAC | 70 |
| *TFF3* | >NM_003226.4 | Fw 5’-->3’: CCAAGGAGTGCAACAACCG Rv 5’-->3’: AGGTGCATTCTGCTTCCTG | 93 |
| *TUBB2B* | >NM_178012.4 | Fw 5’-->3’: CAGGCACGATGGATTCGGTTA Rv 5’-->3’: CACTCTGGCCAAACACGAAAT | 78 |
| *UGT2B7* | >NM_001074.4  >NM_001330719.2  >NM_001349568.2 | Fw 5’-->3’: ACTCCTGGAATTTTCAGTTTCCATA Rv 5’-->3’: TCCATTTCCTTAGGCAGGGGT | 100 |
| *VCP* | >NM_007126.4  >NM_001354927.1  >NM_001354928.1 | Fw 5’-->3’: CATCAGCCATGGAGGTAGAAGA Rv 5’-->3’: GGGTCTGGGCAAACATCTCAT | 135 |

**Table S3** Equipotent binary and ternary mixtures of PHP, PHX and CTD tested in reporter gene assays

|  | PHX + PHP | | | | | PHX + CTD | | | | | |
| --- | --- | --- | --- | --- | --- | --- | --- | --- | --- | --- | --- |
| label | PHXeq [µM] | | PHX [µM] | | PHP [µM] | PHXeq [µM] | | PHX [µM] | | CTD [µM] | |
|  |  | | RPF 1 | | RPF 1.3 |  | | RPF 1 | | RPF 4.5 | |
| Mix 6 | 4000 | | 2000 | | 1538 | 4000 | | 2000 | | 444 | |
| Mix 5 | 2000 | | 1000 | | 769 | 2000 | | 1000 | | 222 | |
| Mix 4 | 1000 | | 500 | | 385 | 1000 | | 500 | | 111 | |
| Mix 3 | 500 | | 250 | | 192 | 500 | | 250 | | 56 | |
| Mix 2 | 250 | | 125 | | 96 | 250 | | 125 | | 28 | |
| Mix 1 | 125 | | 62.5 | | 48 | 125 | | 62.5 | | 14 | |
|  | PHP + CTD | | | | | PHX + PHP + CTD | | | | | |
| label | PHPeq [µM] | PHP [µM] | | CTD [µM] | | PHXeq [µM] | PHX [µM] | | PHP [µM] | | CTD [µM] |
|  |  | RPF 1 | | RPF 3.6 | |  | RPF 1 | | RPF 1.3 | | RPF 4.5 |
| Mix 6 | 4000 | 2000 | | 556 | | 6000 | 2000 | | 1538 | | 444 |
| Mix 5 | 2000 | 1000 | | 278 | | 3000 | 1000 | | 769 | | 222 |
| Mix 4 | 1000 | 500 | | 139 | | 1500 | 500 | | 385 | | 111 |
| Mix 3 | 500 | 250 | | 69 | | 750 | 250 | | 192 | | 56 |
| Mix 2 | 250 | 125 | | 35 | | 375 | 125 | | 96 | | 28 |
| Mix 1 | 125 | 62.5 | | 17 | | 187.5 | 62.5 | | 48 | | 14 |

The final concentration of e.g. 1000 µM PHX equivalents (PHXeq) in a mixture is composed of 1000 µM/2 = 500 µM PHX and 1000 µM/2/RPF1.3 = 385 µM PHP.

**
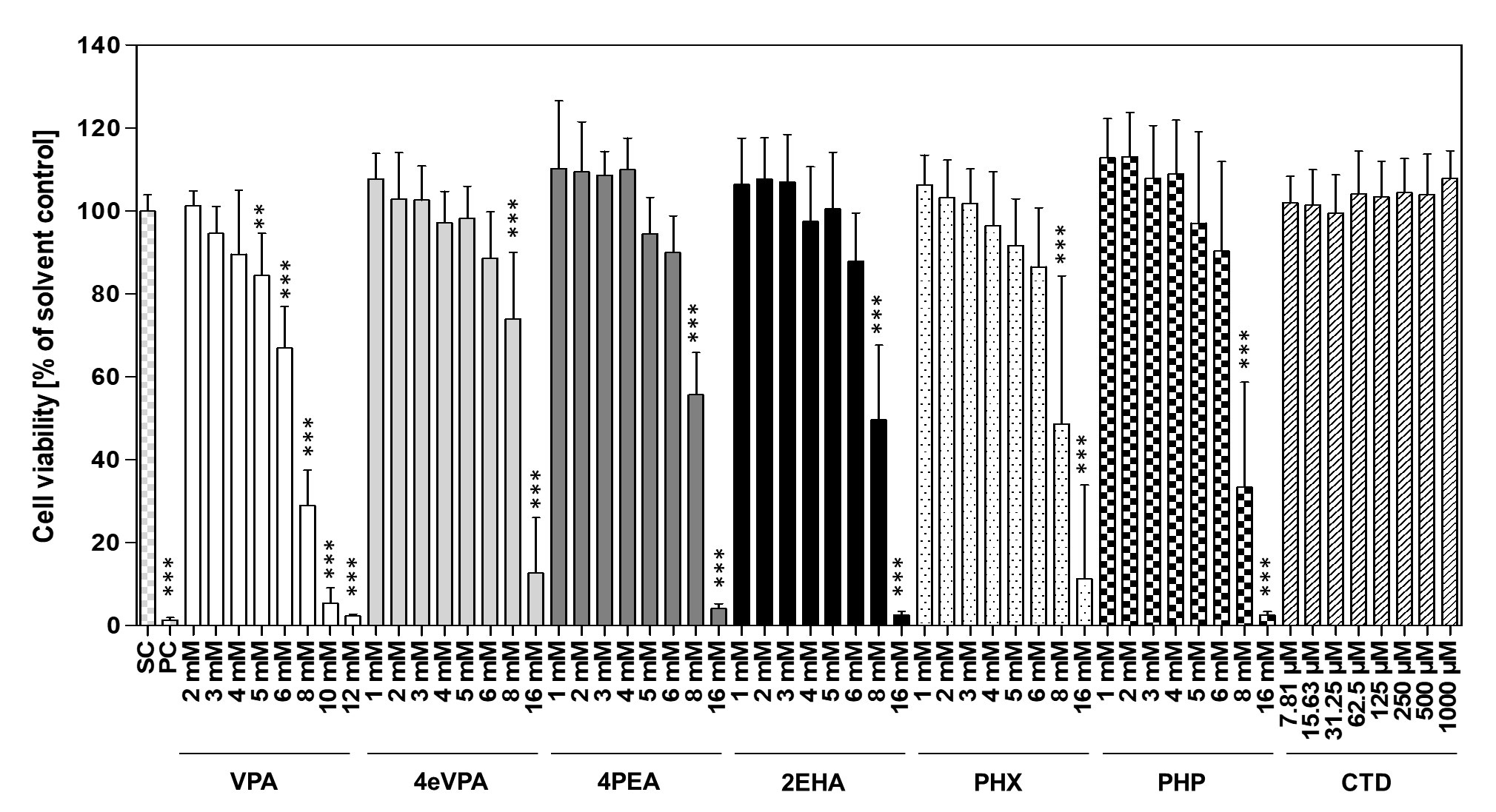
**

**Fig. S1** Cell viability of HepaRG cells exposed to VPA, 4eVPA, 4PEA, 2EHA, PHX, PHP, and CTD for 72 h. Differentiated HepaRG cells were exposed to different concentrations of test compounds or solvent control (0.5% DMSO) for 72 h. Cytotoxicity of test compounds was assessed using WST-1 assay. Data expressed in percent of solvent control (SC; 0.5% DMSO) and as means of three independent experiments performed with three replicates each ± SD (**p < 0.01, ***p < 0.001 one-way ANOVA against SC)


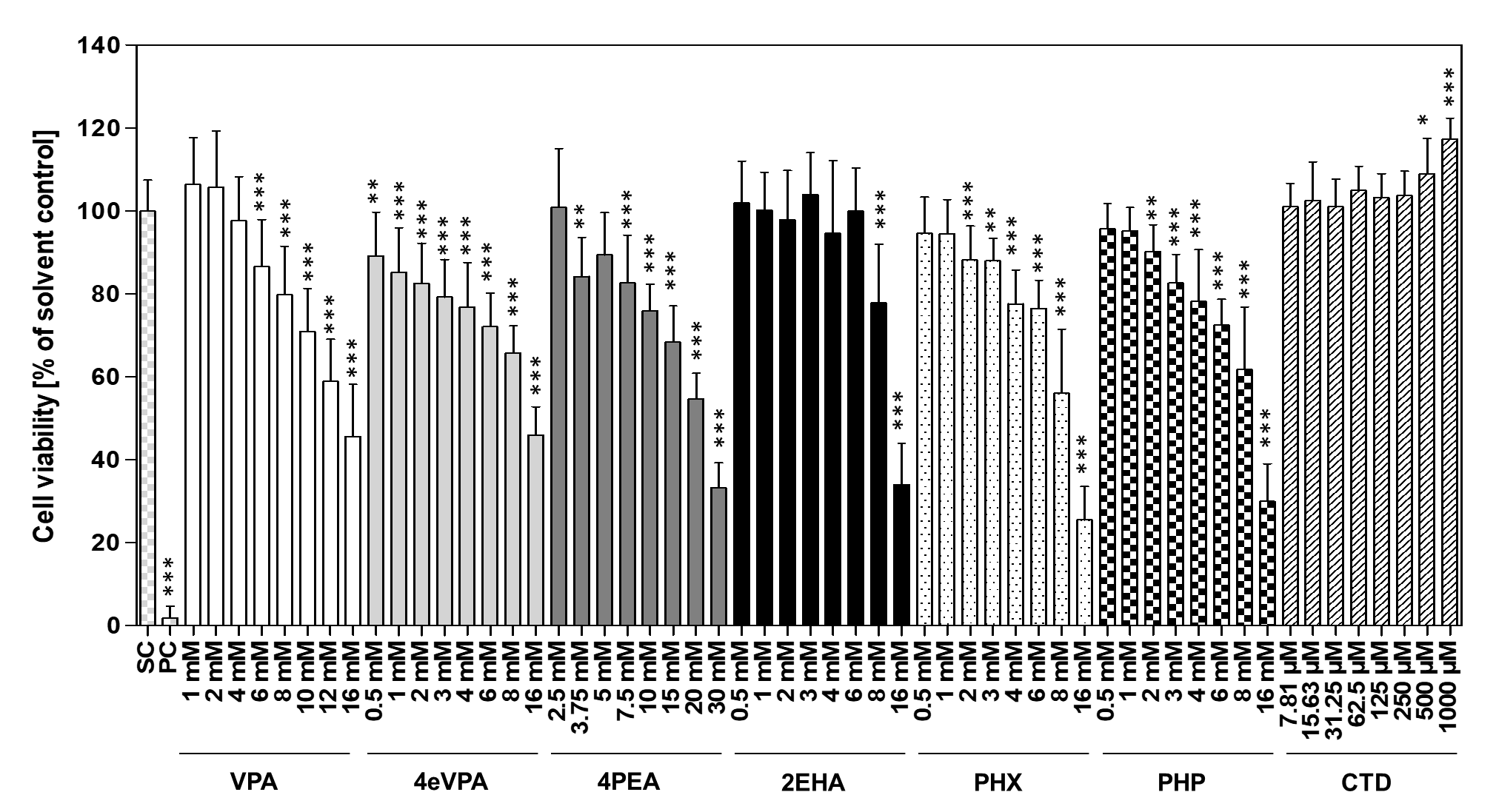


**Fig. S2** Cell viability of HepG2 cells exposed to VPA, 4eVPA, 4PEA, 2EHA, PHX, PHP, and CTD for 24 h. HepG2 cells were exposed to different concentrations of test compounds or solvent control (0.5% DMSO) for 24 h. Cytotoxicity of test compounds was assessed using WST-1 assay. Data expressed in percent of solvent control (SC; 0.5% DMSO) and as means of three independent experiments performed with three replicates each ± SD (*p < 0.05, **p < 0.01, ***p < 0.001 one-way ANOVA against SC)


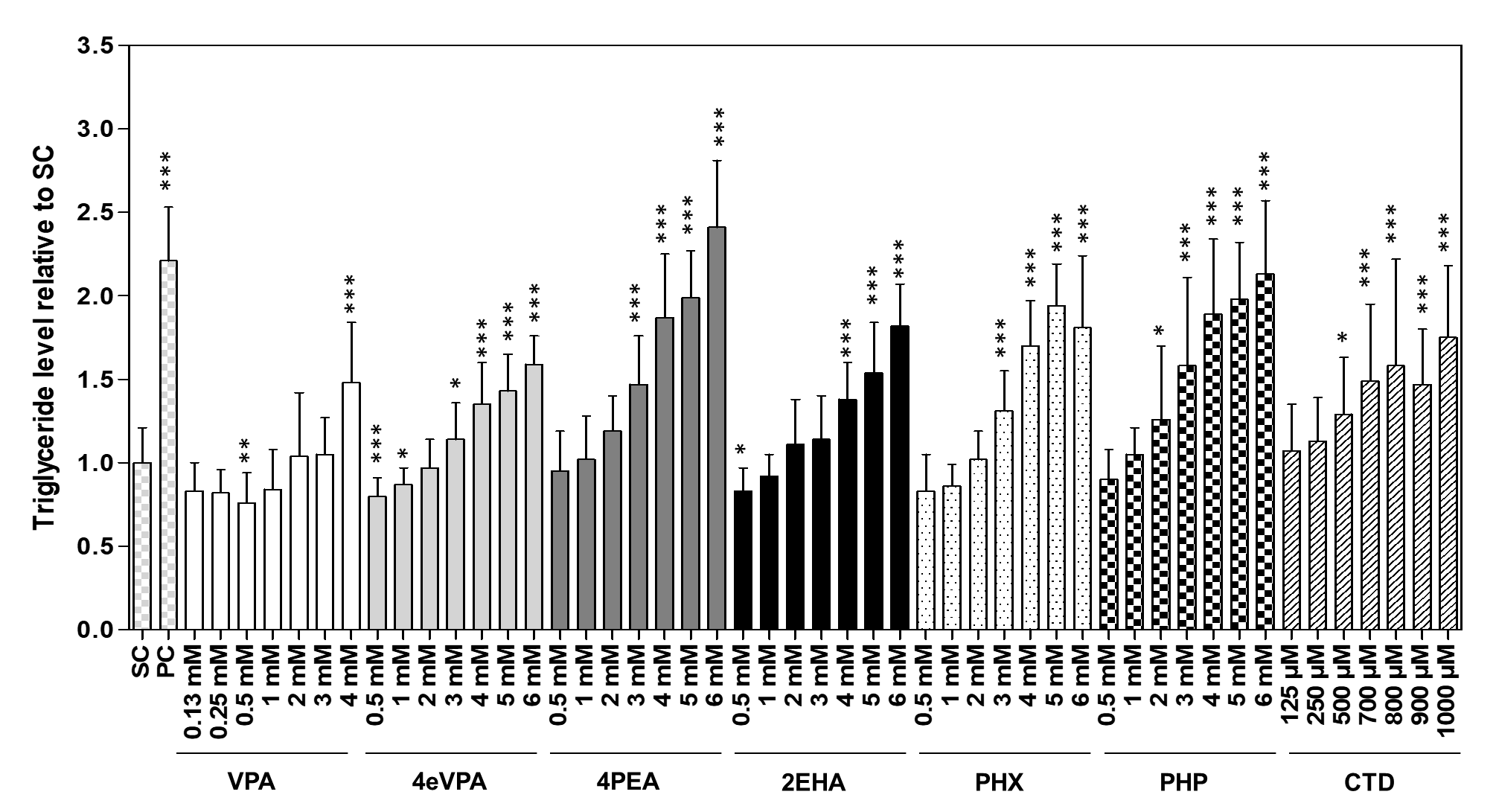


**Fig. S3** Triglyceride accumulation assayed via AdipoRed staining of HepaRG cells exposed to VPA, 4PEA, 4eVPA, 2EHA, PHX, PHP, and CTD for 72 h. Differentiated HepaRG cells were exposed to the different concentrations of test compounds, solvent control (SC; 0.5% DMSO) or positive control (PC; 200 µM cyproconazole) and triglyceride accumulation was analyzed using AdipoRed assay. The triglyceride content was referred to the solvent control. Data are presented as means of three independent experiments performed with three replicates each ± SD (*p < 0.05, **p < 0.01, ***p < 0.001 one-way ANOVA against SC)


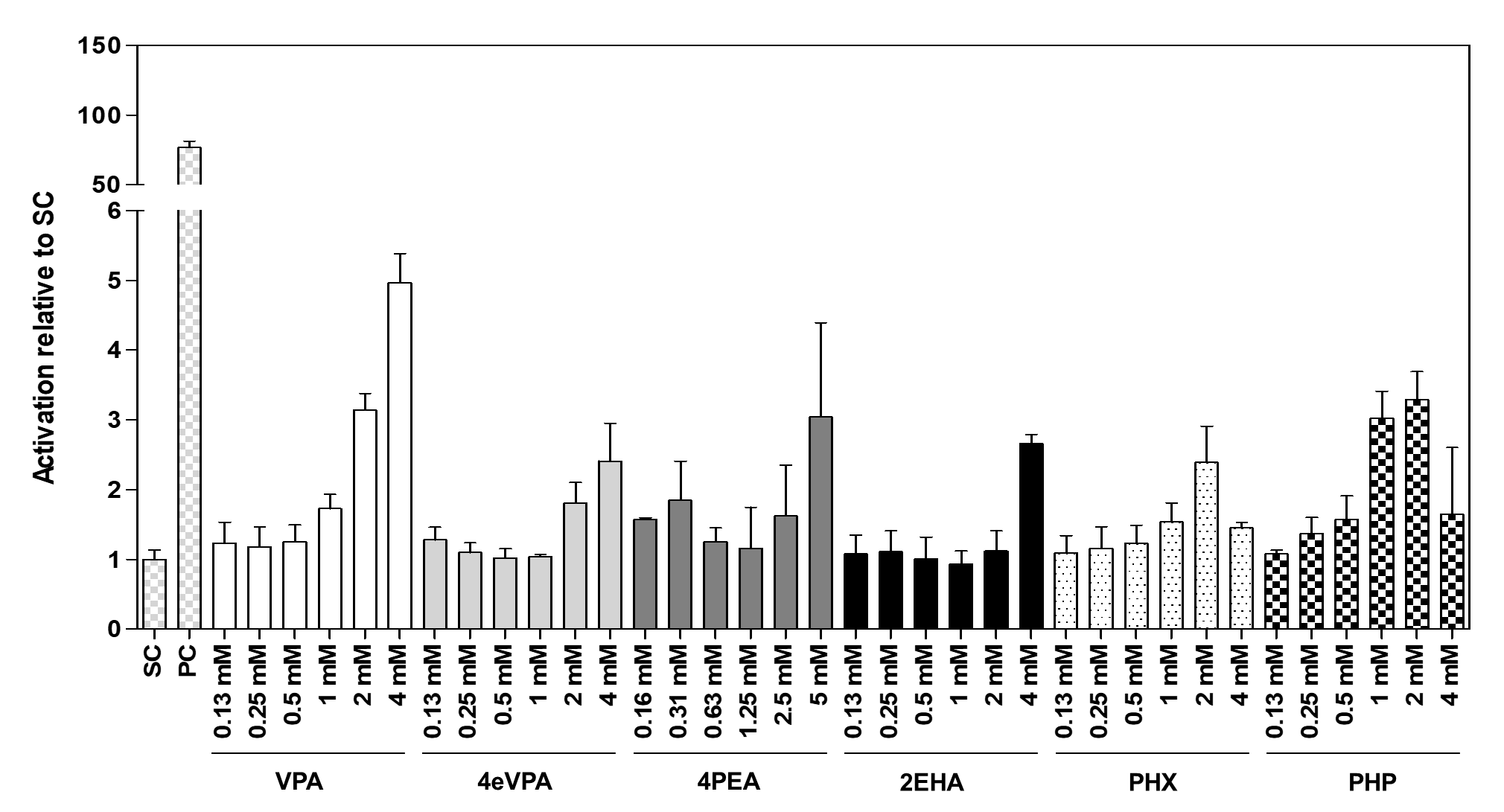


**Fig. S4** AhR activation by VPA and its analogues. HepG2 cells were co-transfected with the appropriated LBD-expressing plasmid, a GAL4-(UAS)5-TK-LUC reporter plasmid as well as with Renilla luciferase plasmid coding pcDNA3-Rluc for normalization and exposed to indicated concentrations of test compounds, solvent control (SC; 0.5% DMSO) or to the positive control (referred to Table S1). After 24 h cell lysate were assayed for firefly and Renilla luciferase activity. Firefly luciferase activity was normalized against Renilla luciferase activity and fold induction relative to solvent control was calculated. Data are presented as means ± SD (only one independent experiment was done, therefore no statistical analysis was performed)


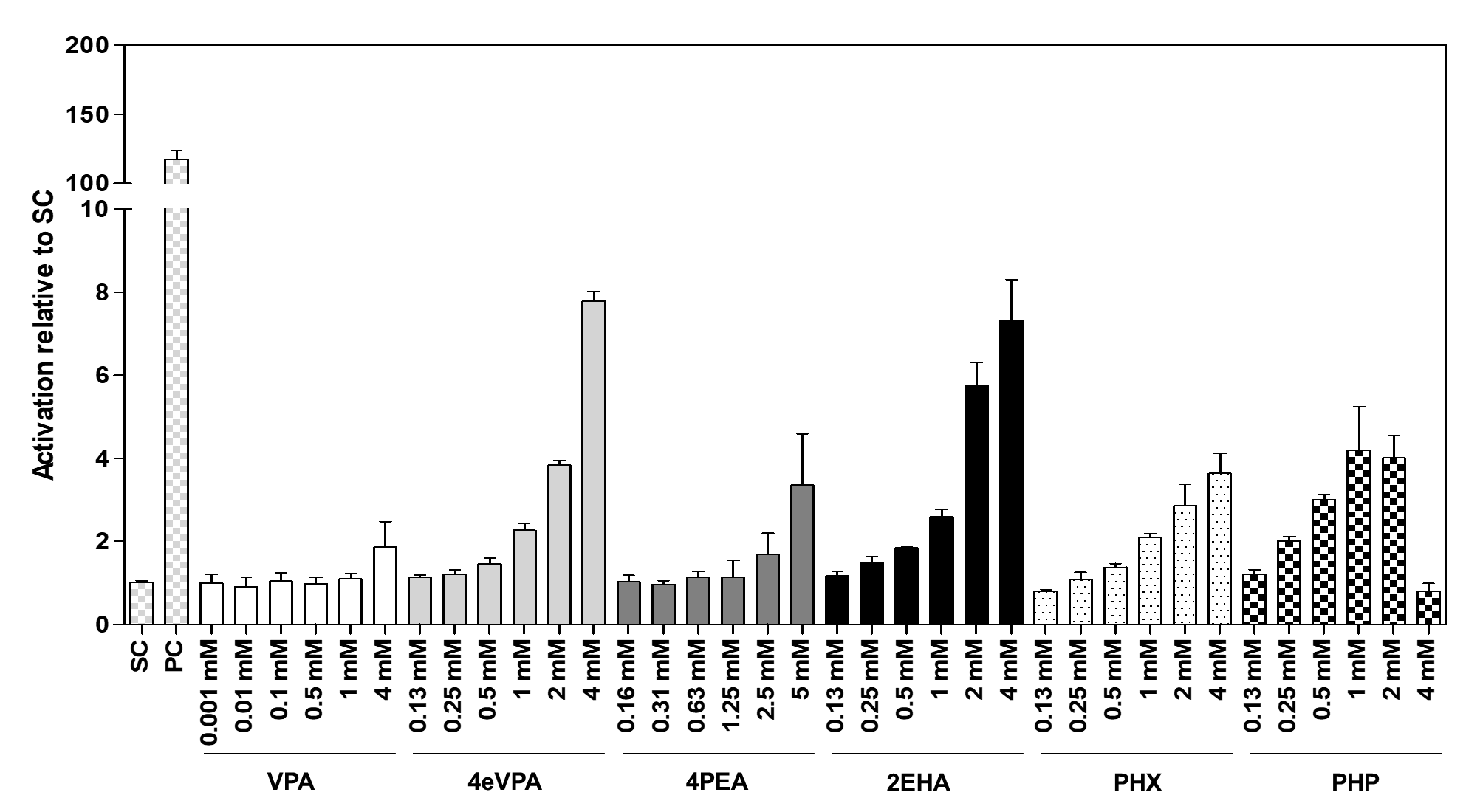


**Fig. S5** CAR activation by VPA and its analogues. HepG2 cells were co-transfected with the appropriated LBD-expressing plasmid, a GAL4-(UAS)5-TK-LUC reporter plasmid as well as with Renilla luciferase plasmid coding pcDNA3-Rluc for normalization and exposed to indicated concentrations of test compounds, solvent control (SC; 0.5% DMSO) or to the positive control (referred to Table S1). After 24 h cell lysate were assayed for firefly and Renilla luciferase activity. Firefly luciferase activity was normalized against Renilla luciferase activity and fold induction relative to solvent control was calculated. Data are presented as means ± SD (only one independent experiment was done, therefore no statistical analysis was performed)


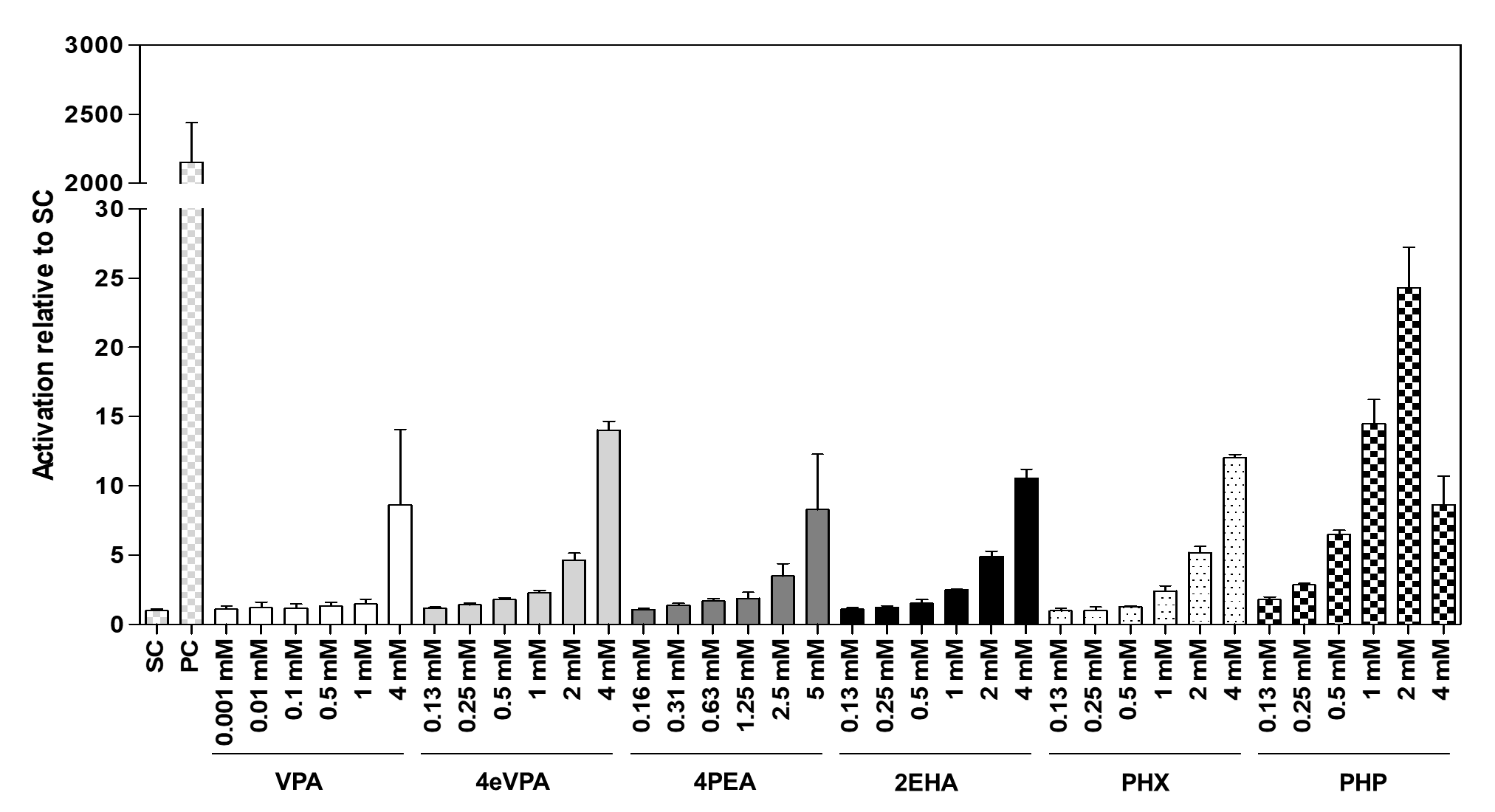


**Fig. S6** FXR activation by VPA and its analogues. HepG2 cells were co-transfected with the appropriated LBD-expressing plasmid, a GAL4-(UAS)5-TK-LUC reporter plasmid as well as with Renilla luciferase plasmid coding pcDNA3-Rluc for normalization and exposed to indicated concentrations of test compounds, solvent control (SC; 0.5% DMSO) or to the positive control (referred to Table S1). After 24 h cell lysate were assayed for firefly and Renilla luciferase activity. Firefly luciferase activity was normalized against Renilla luciferase activity and fold induction relative to solvent control was calculated. Data are presented as means ± SD (only one independent experiment was done, therefore no statistical analysis was performed)

**
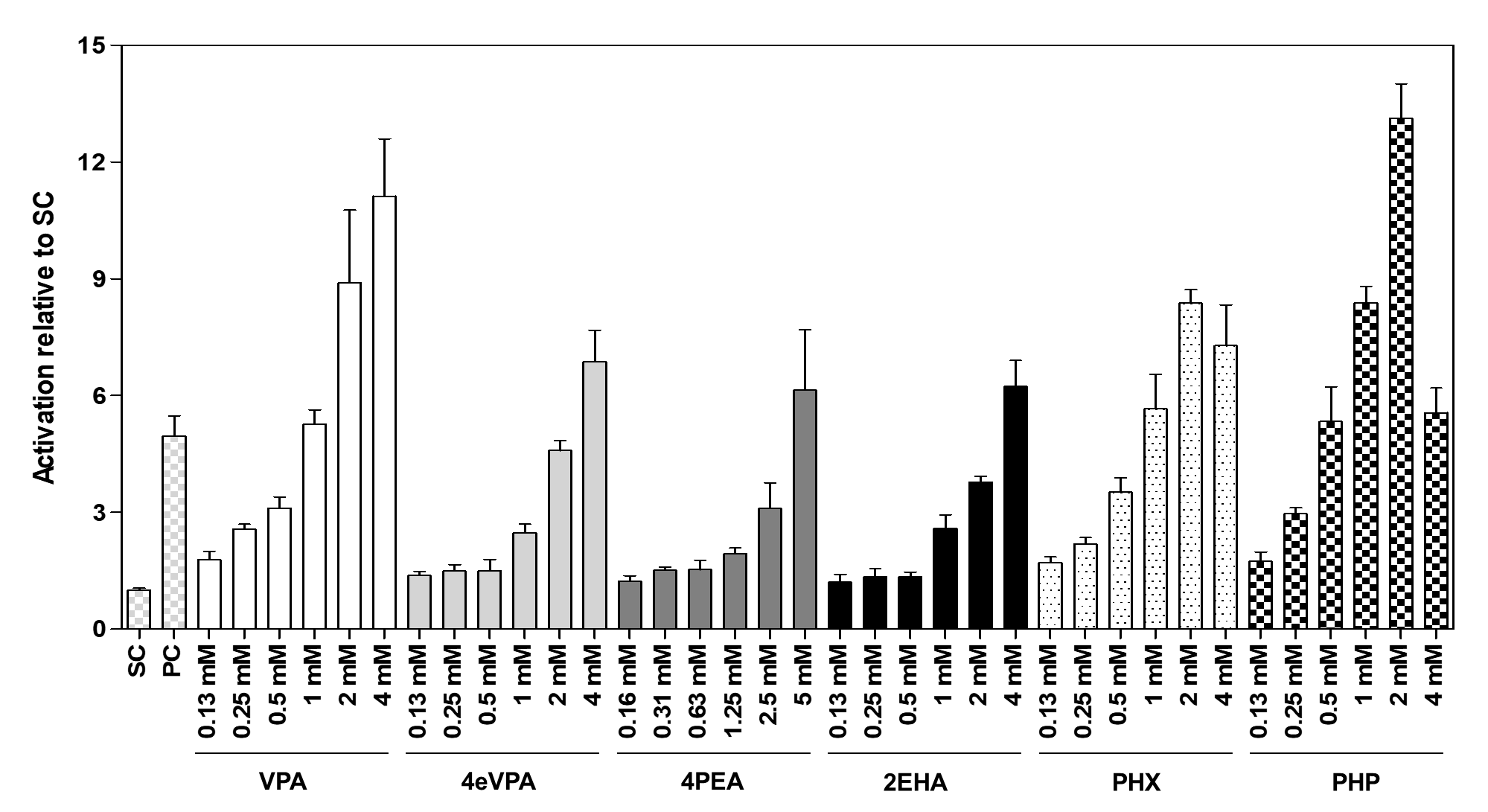
Fig. S7** GR activation by VPA and its analogues. HepG2 cells were co-transfected with the appropriated LBD-expressing plasmid, a GAL4-(UAS)5-TK-LUC reporter plasmid as well as with Renilla luciferase plasmid coding pcDNA3-Rluc for normalization and exposed to indicated concentrations of test compounds, solvent control (SC; 0.5% DMSO) or to the positive control (referred to Table S1). After 24 h cell lysate were assayed for firefly and Renilla luciferase activity. Firefly luciferase activity was normalized against Renilla luciferase activity and fold induction relative to solvent control was calculated. Data are presented as means ± SD (only one independent experiment was done, therefore no statistical analysis was performed)

**
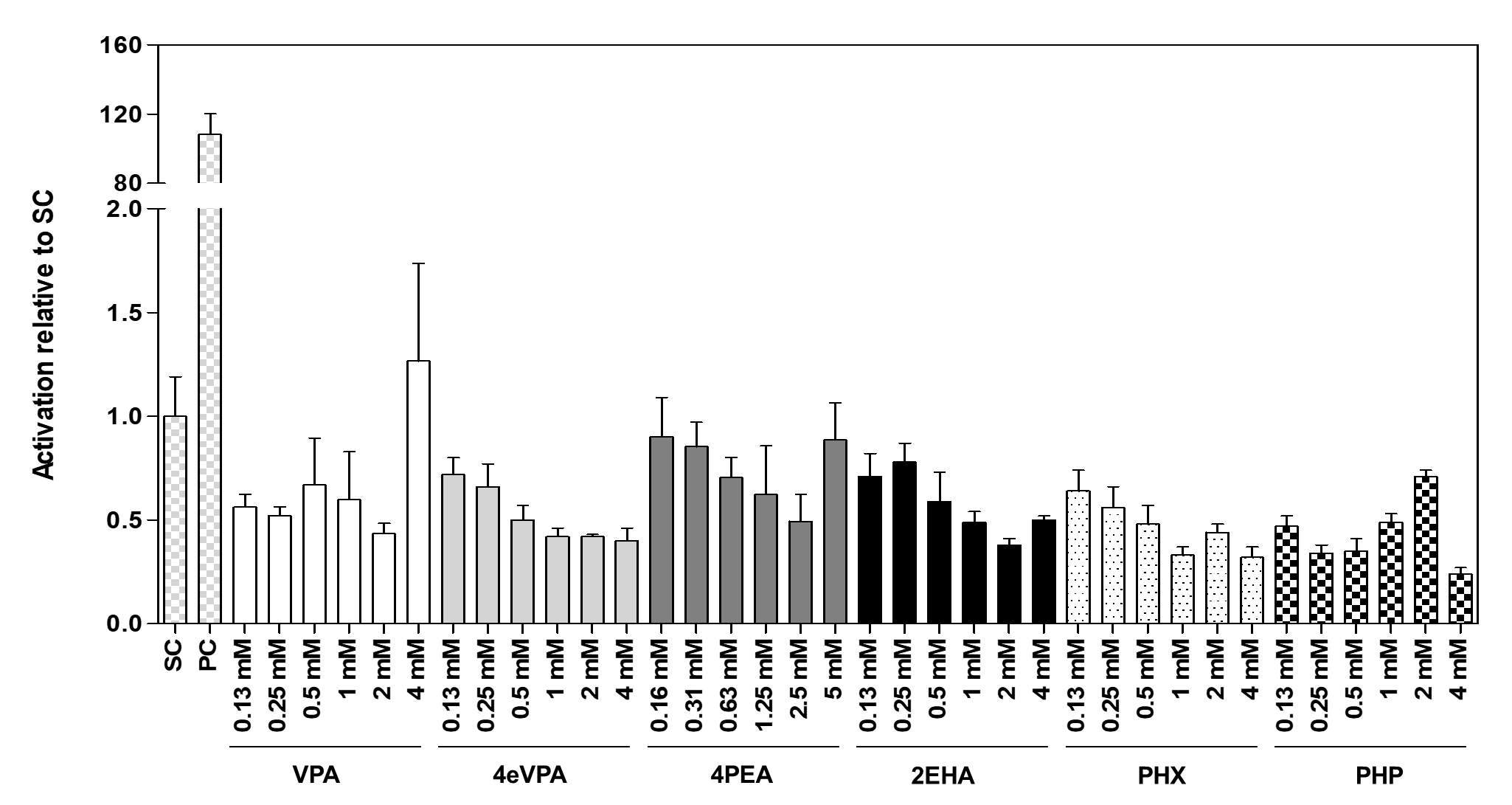
Fig. S8** LXR activation by VPA and its analogues. HepG2 cells were co-transfected with the appropriated LBD-expressing plasmid, a GAL4-(UAS)5-TK-LUC reporter plasmid as well as with Renilla luciferase plasmid coding pcDNA3-Rluc for normalization and exposed to indicated concentrations of test compounds, solvent control (SC; 0.5% DMSO) or to the positive control (referred to Table S1). After 24 h cell lysate were assayed for firefly and Renilla luciferase activity. Firefly luciferase activity was normalized against Renilla luciferase activity and fold induction relative to solvent control was calculated. Data are presented as means ± SD (only one independent experiment was done, therefore no statistical analysis was performed)

**
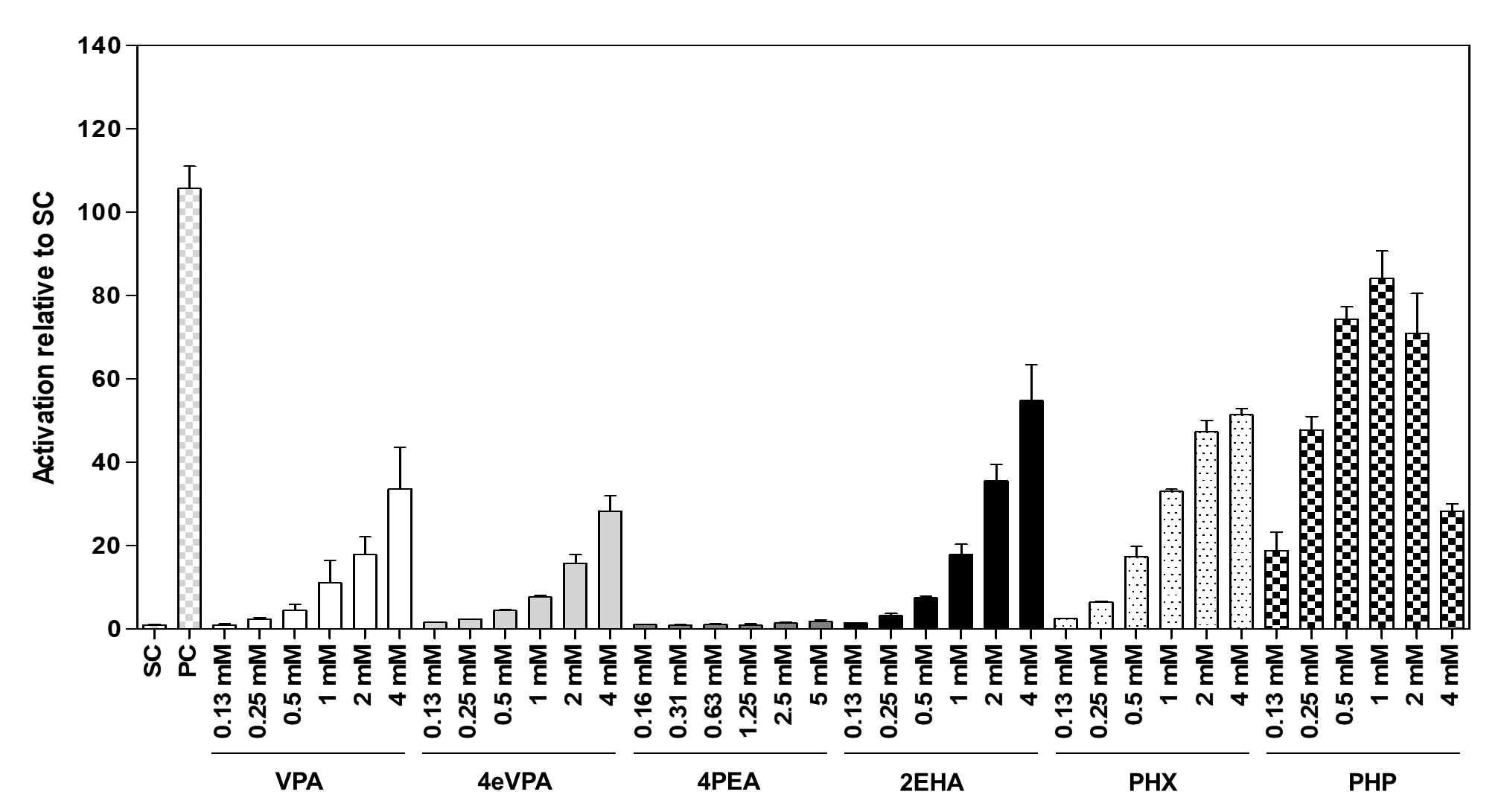
Fig. S9** PPARα activation by VPA and its analogues. HepG2 cells were co-transfected with the appropriated LBD-expressing plasmid, a GAL4-(UAS)5-TK-LUC reporter plasmid as well as with Renilla luciferase plasmid coding pcDNA3-Rluc for normalization and exposed to indicated concentrations of test compounds, solvent control (SC; 0.5% DMSO) or to the positive control (referred to Table S1). After 24 h cell lysate were assayed for firefly and Renilla luciferase activity. Firefly luciferase activity was normalized against Renilla luciferase activity and fold induction relative to solvent control was calculated. Data are presented as means ± SD (only one independent experiment was done, therefore no statistical analysis was performed)

**
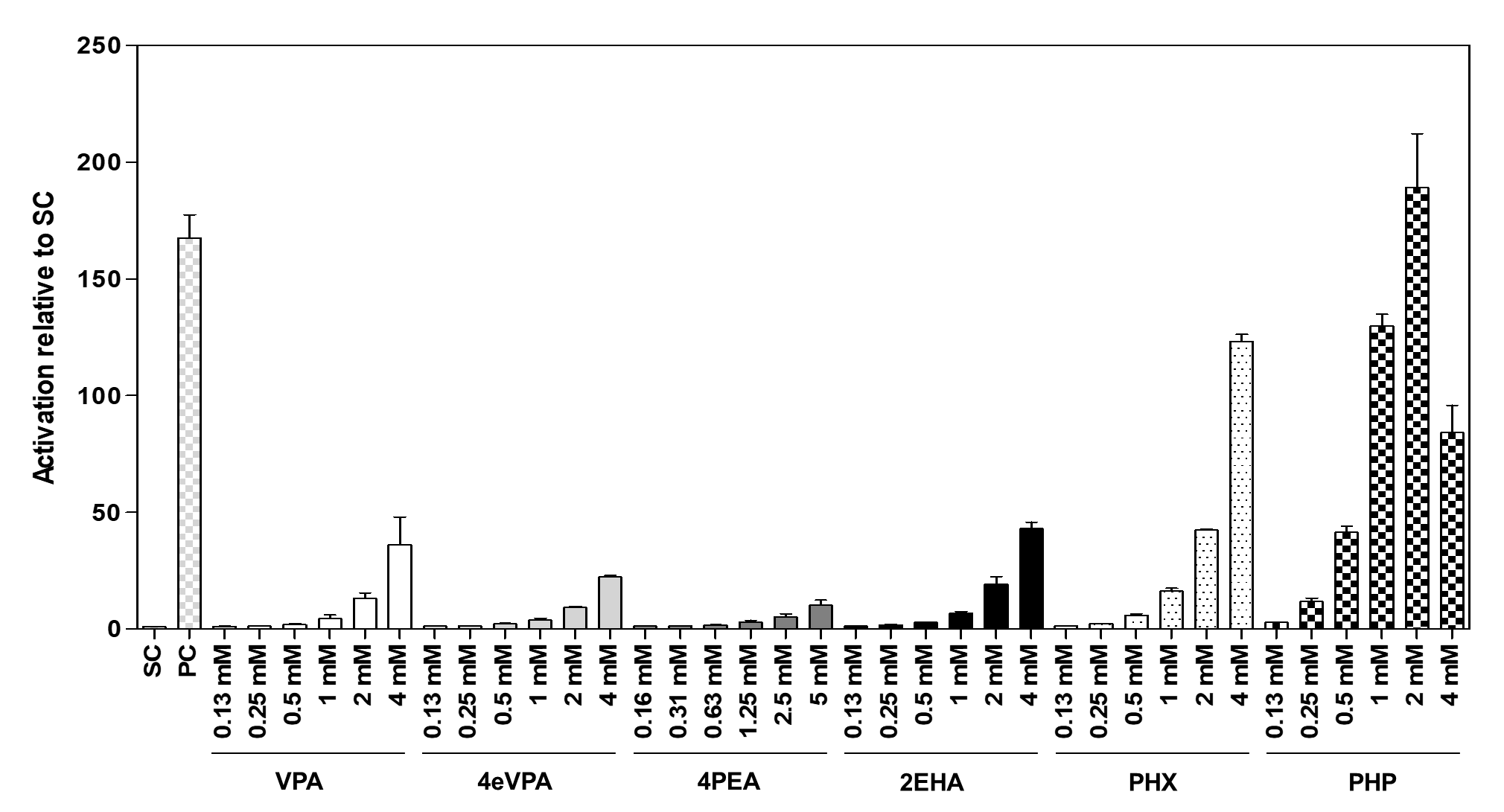
**

**Fig. S10** PPARγ activation by VPA and its analogues. HepG2 cells were co-transfected with the appropriated LBD-expressing plasmid, a GAL4-(UAS)5-TK-LUC reporter plasmid as well as with Renilla luciferase plasmid coding pcDNA3-Rluc for normalization and exposed to indicated concentrations of test compounds, solvent control (SC; 0.5% DMSO) or to the positive control (referred to Table S1). After 24 h cell lysate were assayed for firefly and Renilla luciferase activity. Firefly luciferase activity was normalized against Renilla luciferase activity and fold induction relative to solvent control was calculated. Data are presented as means ± SD (only one independent experiment was done, therefore no statistical analysis was performed)


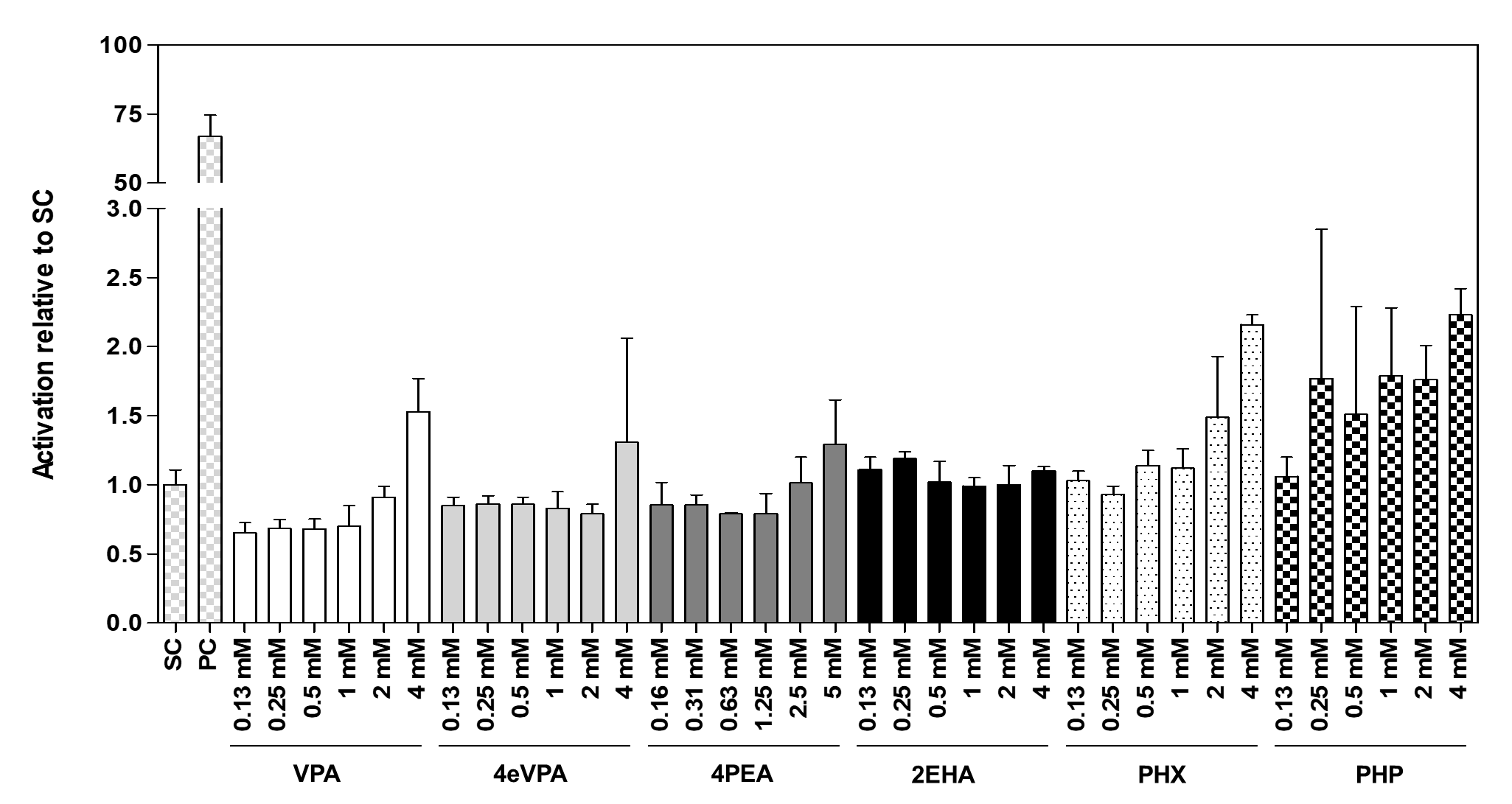


**Fig. S11** PPARδ activation by VPA and its analogues. HepG2 cells were co-transfected with the appropriated LBD-expressing plasmid, a GAL4-(UAS)5-TK-LUC reporter plasmid as well as with Renilla luciferase plasmid coding pcDNA3-Rluc for normalization and exposed to indicated concentrations of test compounds, solvent control (SC; 0.5% DMSO) or to the positive control (referred to Table S1). After 24 h cell lysate were assayed for firefly and Renilla luciferase activity. Firefly luciferase activity was normalized against Renilla luciferase activity and fold induction relative to solvent control was calculated. Data are presented as means ± SD (only one independent experiment was done, therefore no statistical analysis was performed)


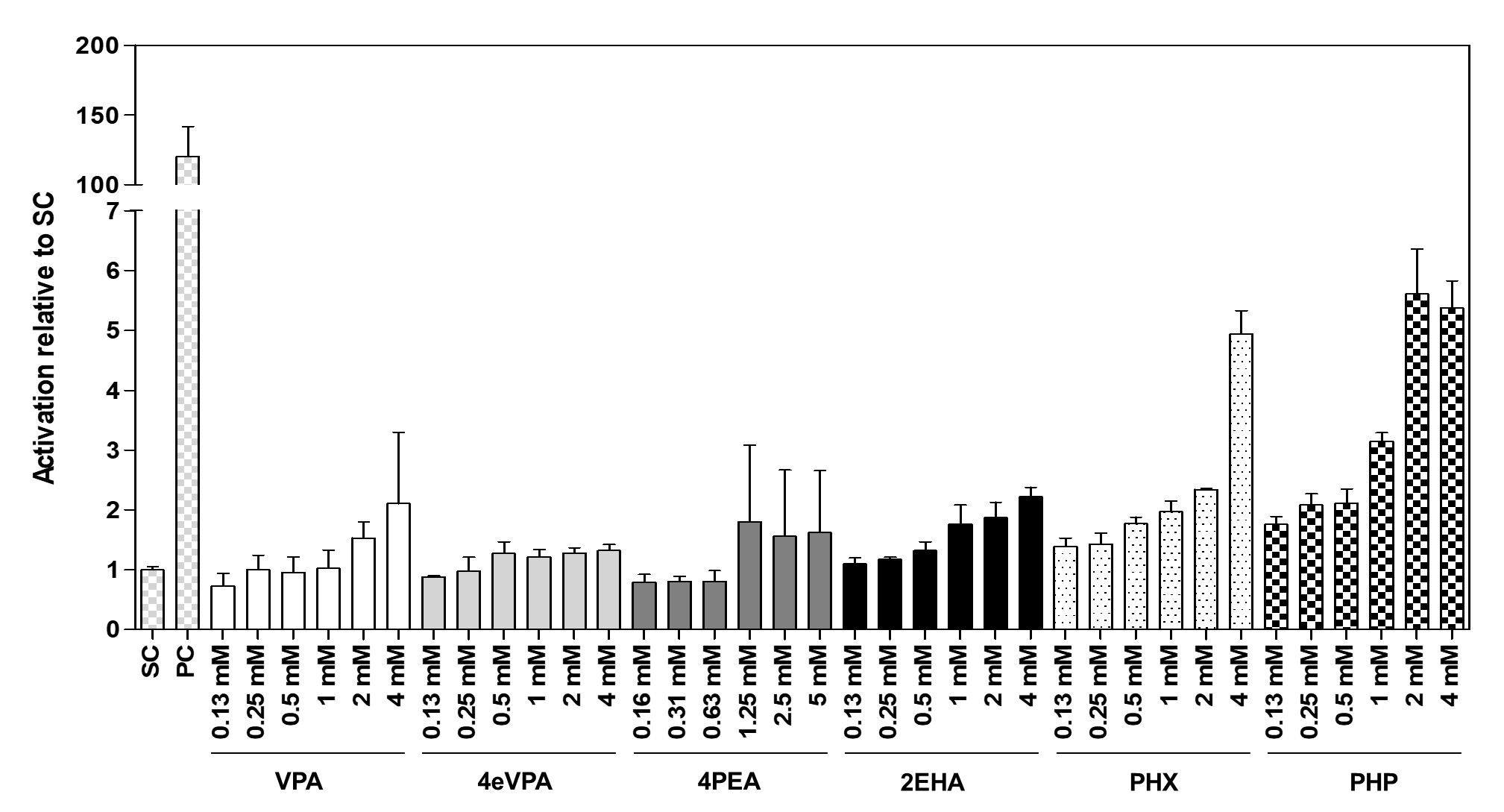


**Fig. S12** PXR activation by VPA and its analogues. HepG2 cells were co-transfected with the appropriated LBD-expressing plasmid, a GAL4-(UAS)5-TK-LUC reporter plasmid as well as with Renilla luciferase plasmid coding pcDNA3-Rluc for normalization and exposed to indicated concentrations of test compounds, solvent control (SC; 0.5% DMSO) or to the positive control (referred to Table S1). After 24 h cell lysate were assayed for firefly and Renilla luciferase activity. Firefly luciferase activity was normalized against Renilla luciferase activity and fold induction relative to solvent control was calculated. Data are presented as means ± SD (only one independent experiment was done, therefore no statistical analysis was performed)


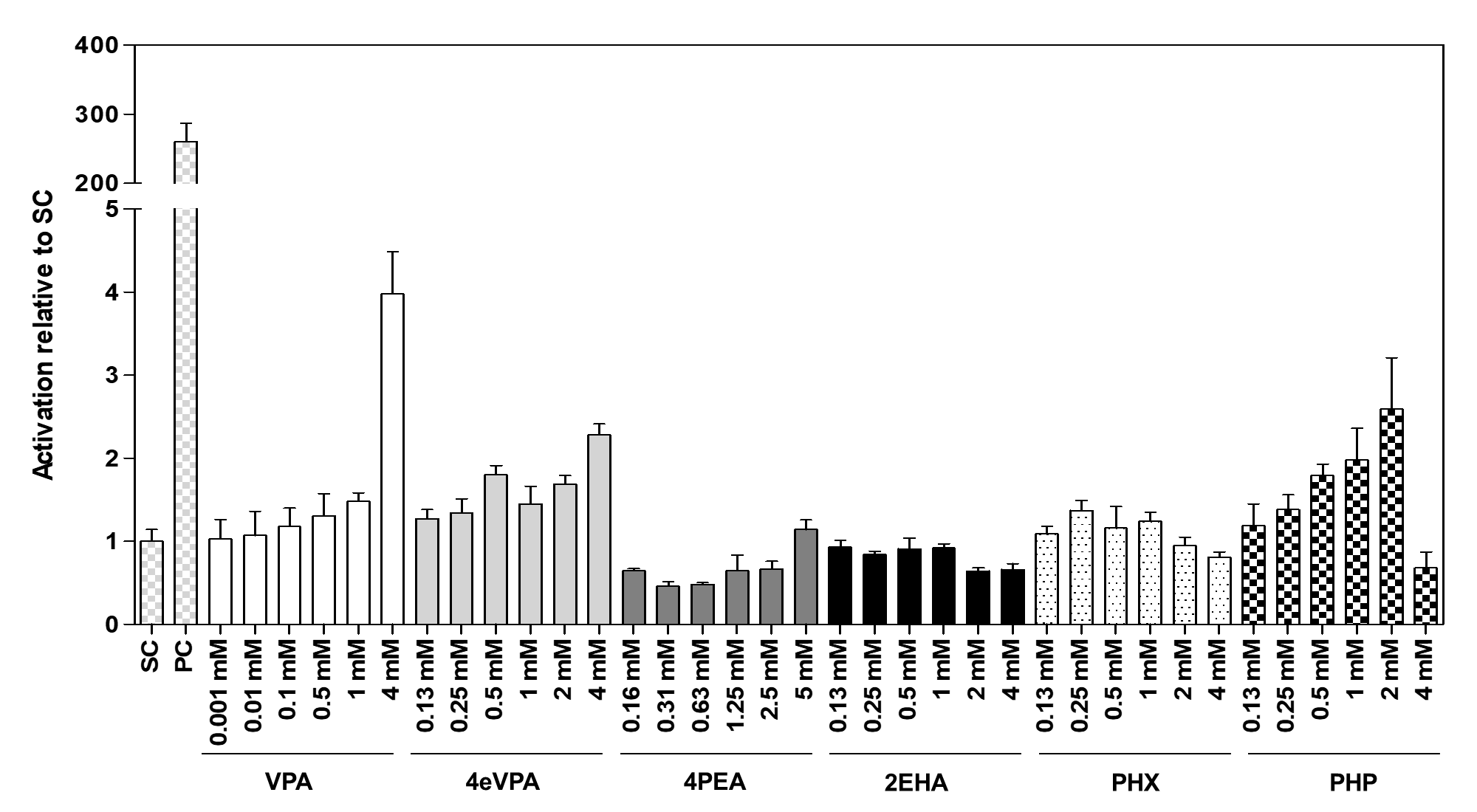


**Fig. S13** RARα activation by VPA and its analogues. HepG2 cells were co-transfected with the appropriated LBD-expressing plasmid, a GAL4-(UAS)5-TK-LUC reporter plasmid as well as with Renilla luciferase plasmid coding pcDNA3-Rluc for normalization and exposed to indicated concentrations of test compounds, solvent control (SC; 0.5% DMSO) or to the positive control (referred to Table S1). After 24 h cell lysate were assayed for firefly and Renilla luciferase activity. Firefly luciferase activity was normalized against Renilla luciferase activity and fold induction relative to solvent control was calculated. Data are presented as means ± SD (only one independent experiment was done, therefore no statistical analysis was performed)


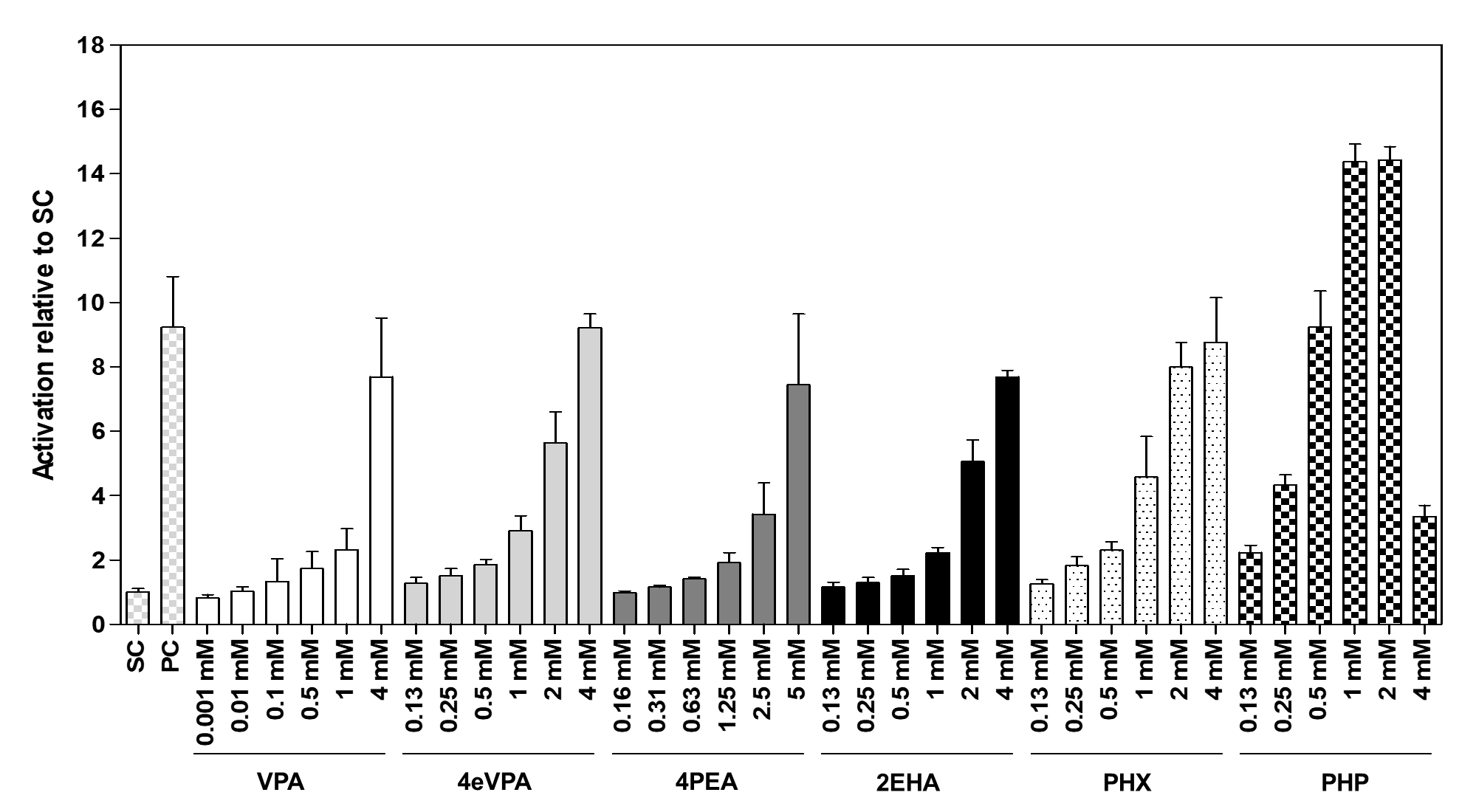


**Fig. S14** RXRα activation by VPA and its analogues. HepG2 cells were co-transfected with the appropriated LBD-expressing plasmid, a GAL4-(UAS)5-TK-LUC reporter plasmid as well as with Renilla luciferase plasmid coding pcDNA3-Rluc for normalization and exposed to indicated concentrations of test compounds, solvent control (SC; 0.5% DMSO) or to the positive control (referred to Table S1). After 24 h cell lysate were assayed for firefly and Renilla luciferase activity. Firefly luciferase activity was normalized against Renilla luciferase activity and fold induction relative to solvent control was calculated. Data are presented as means ± SD (only one independent experiment was done, therefore no statistical analysis was performed)


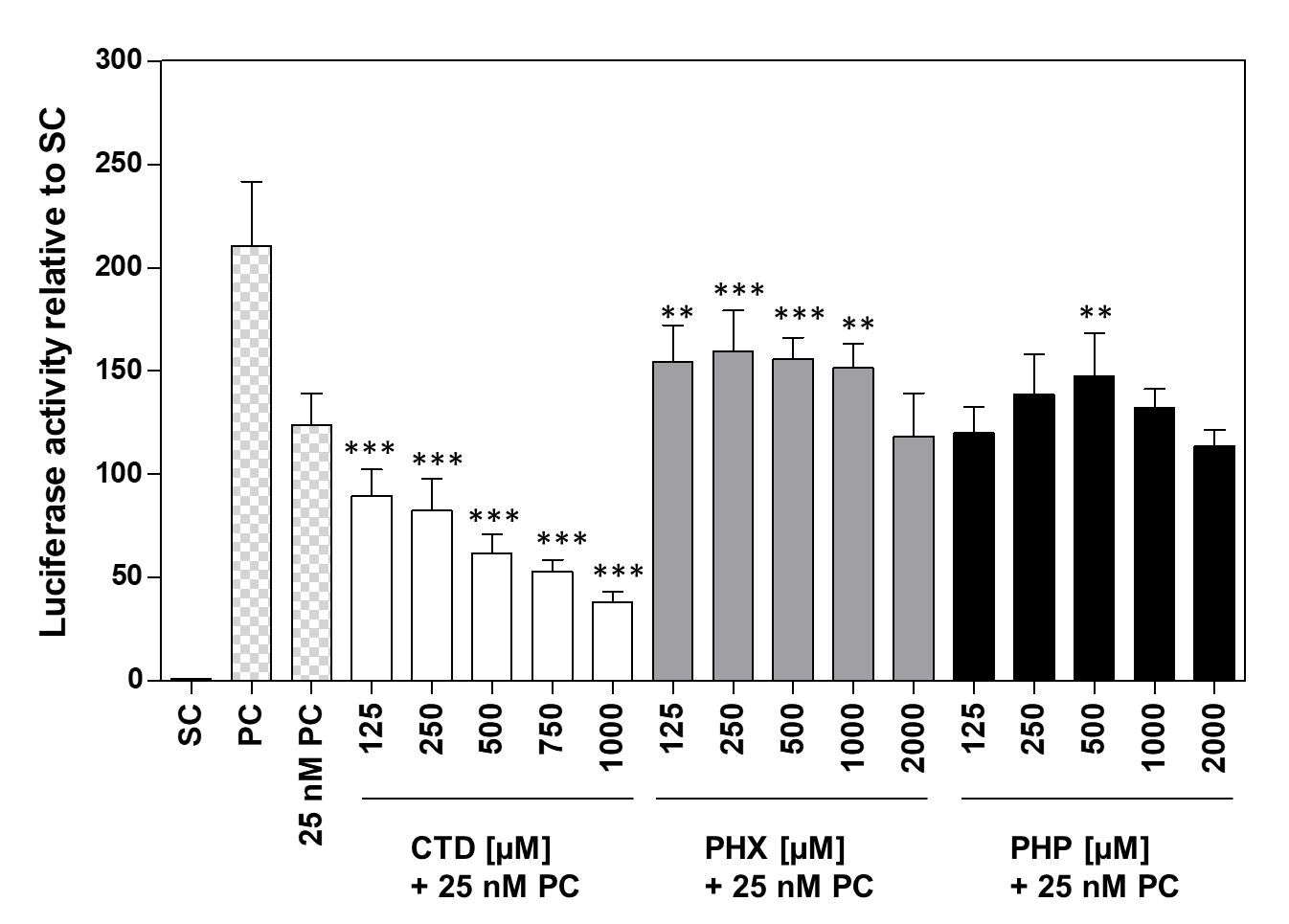


**Fig. S15** PPARα antagonism. A transactivation assay was used to analyze the interaction of test compounds with the ligand binding domain (LBD) of PPARα after PPARα agonist-induced induction. HepG2 cells were transfected with PPARα LBD expression plasmid (pGAL4-hPPARα-LBD) and co-transfected with a GAL4-(UAS)5-TK-LUC reporter plasmid as well as with *Renilla* luciferase plasmid coding pcDNA3-Rluc for normalization and exposed to indicated concentrations of pesticide + 25 nM PPARα agonist GW7647 (EC_50_ of GW7647), solvent control (SC; 0.5% DMSO) or to the positive control (PC; 1 µM GW7647). After 24 h cell lysate were assayed for firefly and *Renilla* luciferase activity. Firefly luciferase activity was normalized against *Renilla* luciferase activity and fold induction relative to solvent control (SC; 0.5% DMSO) was calculated. Data are presented as means ± SD (**p < 0.01, ***p < 0.001 one-way ANOVA against 25 nM PC)


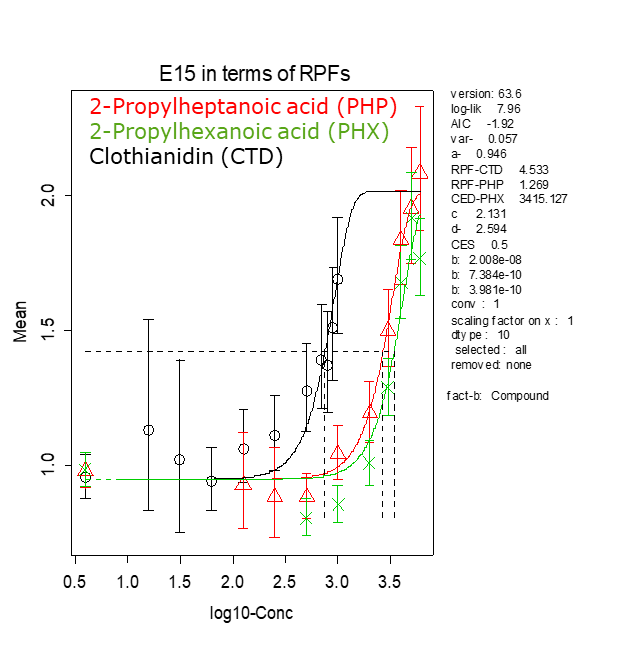


**Fig. S16** Concentration-response modelling for RPF determination via PROAST. Mean values of triglyceride level (Adipored assay) in HepaRG cells as a function of dose, related to CTD (black circles), PHP (red triangles) and PHX (green crosses).The calculated RPF is given in the right-hand-side PROAST annotation: RPF of PHX = 1 (reference compound); RPF of PHP = 1.3; RPF CTD = 4.5


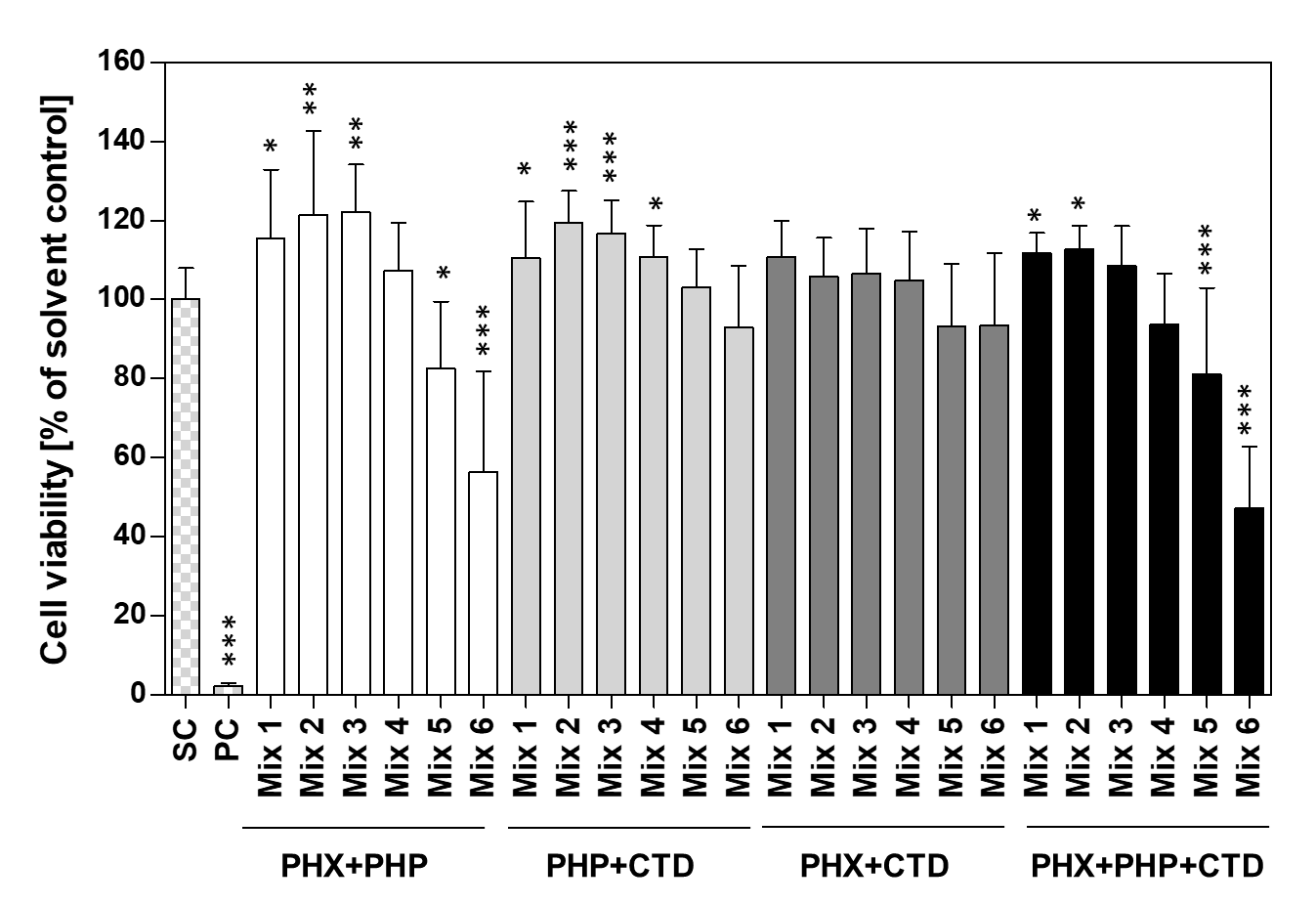


**Fig. S17** Cell viability of HepaRG cells exposed to mixtures of PHX, PHP, and CTD for 72 h. Differentiated HepaRG cells were exposed to different concentrations of test compounds or solvent control (0.5% DMSO) for 72 h. Cytotoxicity of test compounds was assessed using WST-1 assay. Data expressed in percent of solvent control (SC; 0.5% DMSO) and as means of three independent experiments performed with three replicates each ± SD (*p < 0.05, **p < 0.01, ***p < 0.001 one-way ANOVA against SC)


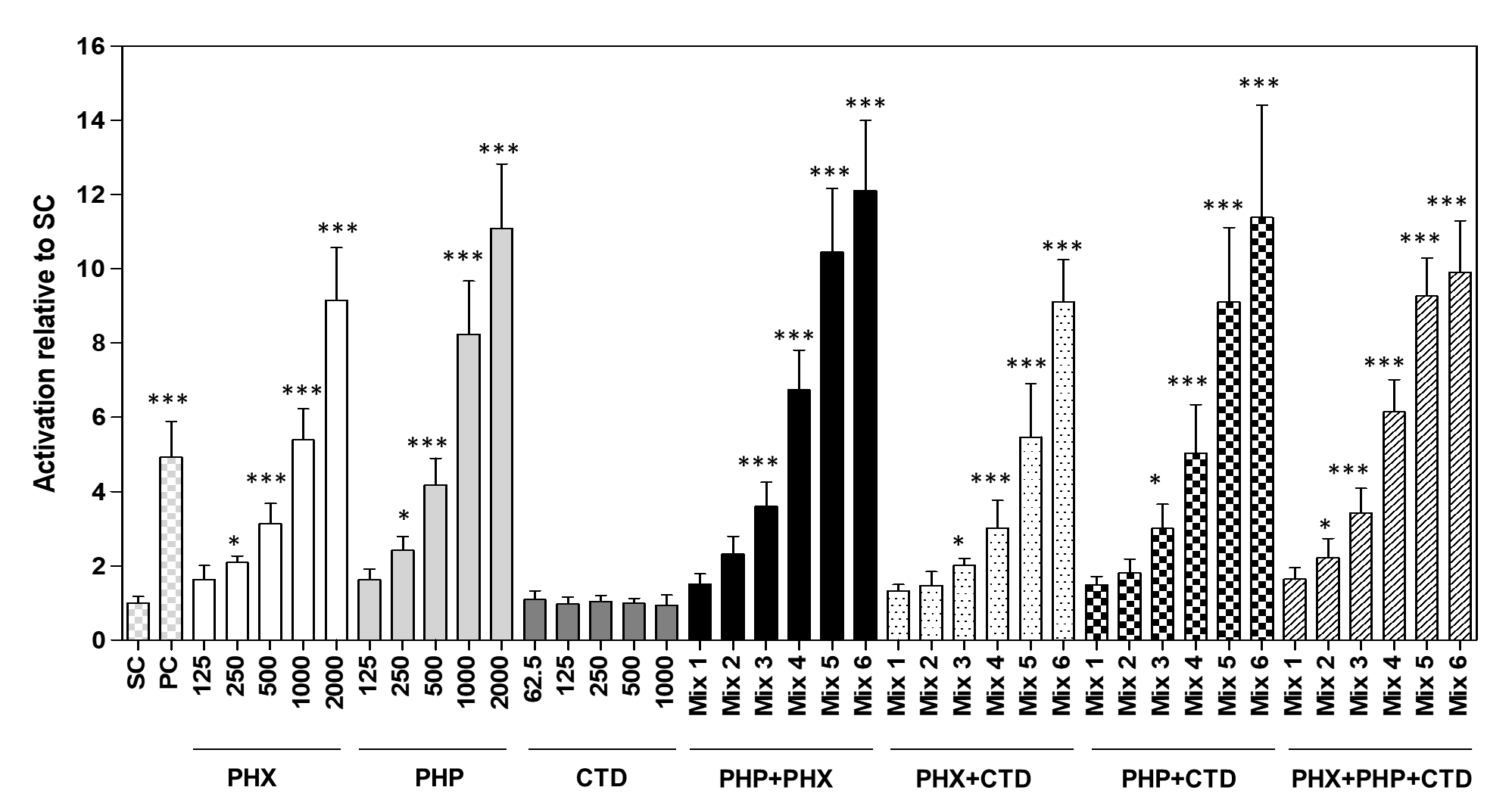


**Fig. S18** GR activation by PHX, PHP, CTD and their mixtures. HepG2 cells were co-transfected with the appropriated LBD-expressing plasmid, a GAL4-(UAS)5-TK-LUC reporter plasmid as well as with Renilla luciferase plasmid coding pcDNA3-Rluc for normalization and exposed to indicated concentrations of test compounds, solvent control (SC; 0.5% DMSO) or to the positive control (referred to Table S1). After 24 h cell lysate were assayed for firefly and Renilla luciferase activity. Firefly luciferase activity was normalized against Renilla luciferase activity and fold induction relative to solvent control was calculated. Data are presented as means ± SD (*p < 0.05, ***p < 0.001 one-way ANOVA against SC)


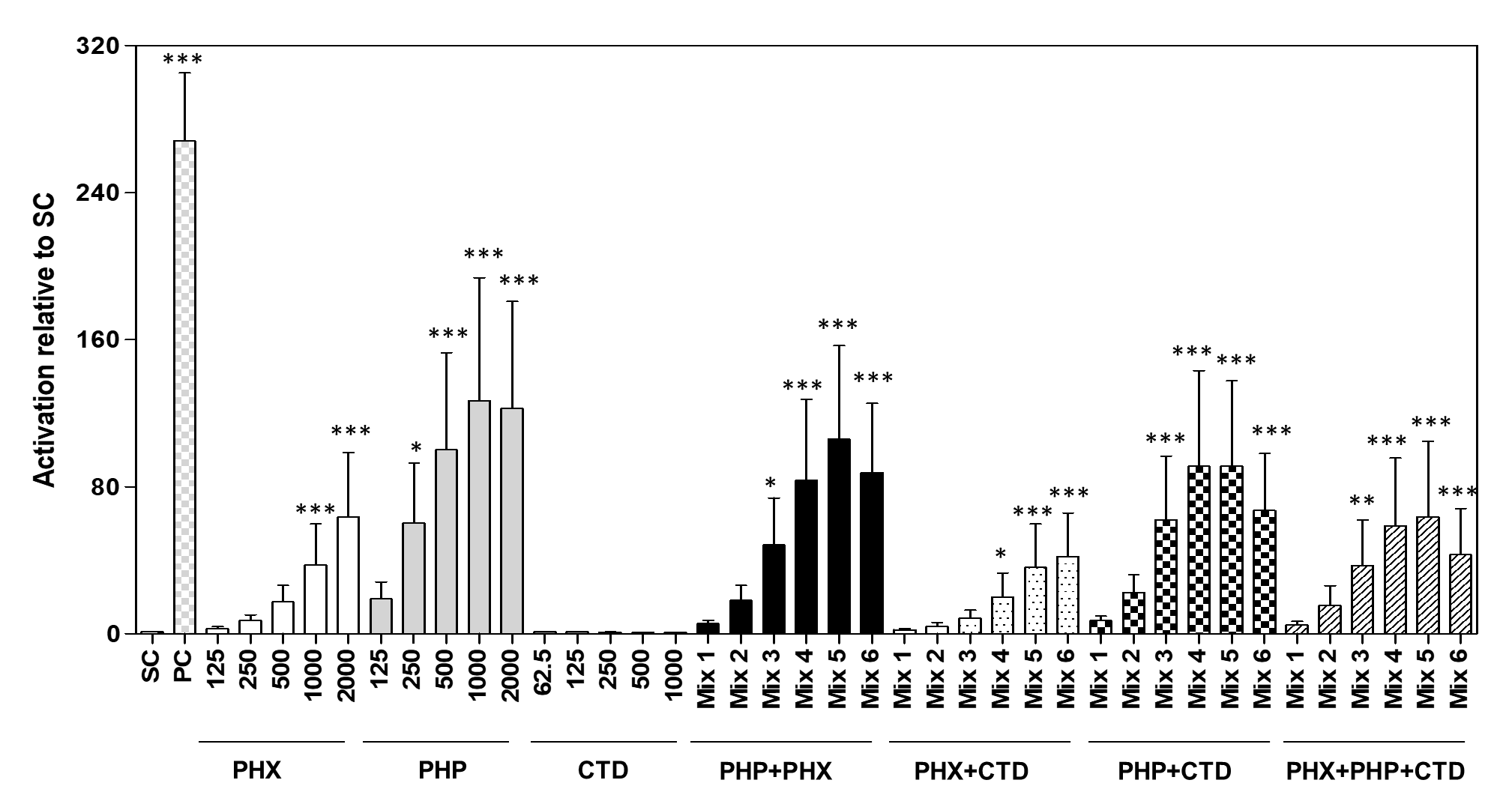


**Fig. S19** PPARα activation by PHX, PHP, CTD and their mixtures. HepG2 cells were co-transfected with the appropriated LBD-expressing plasmid, a GAL4-(UAS)5-TK-LUC reporter plasmid as well as with Renilla luciferase plasmid coding pcDNA3-Rluc for normalization and exposed to indicated concentrations of test compounds, solvent control (SC; 0.5% DMSO) or to the positive control (referred to Table S1). After 24 h cell lysate were assayed for firefly and Renilla luciferase activity. Firefly luciferase activity was normalized against Renilla luciferase activity and fold induction relative to solvent control was calculated. Data are presented as means ± SD (*p < 0.05, **p < 0.01, ***p < 0.001 one-way ANOVA against SC)


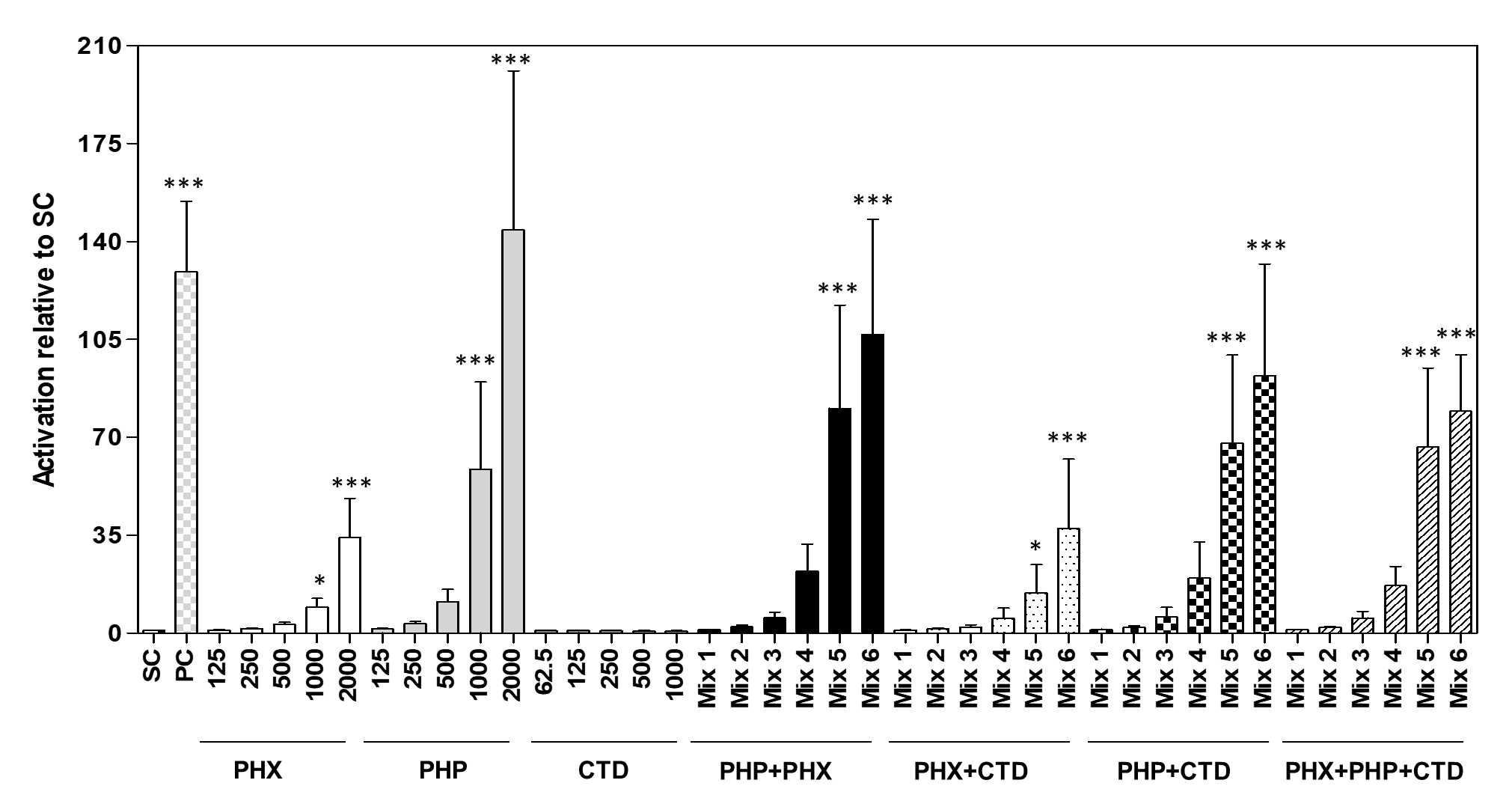


**Fig. S20** PPARγ activation by PHX, PHP, CTD and their mixtures. HepG2 cells were co-transfected with the appropriated LBD-expressing plasmid, a GAL4-(UAS)5-TK-LUC reporter plasmid as well as with Renilla luciferase plasmid coding pcDNA3-Rluc for normalization and exposed to indicated concentrations of test compounds, solvent control (SC; 0.5% DMSO) or to the positive control (referred to Table S1). After 24 h cell lysate were assayed for firefly and Renilla luciferase activity. Firefly luciferase activity was normalized against Renilla luciferase activity and fold induction relative to solvent control was calculated. Data are presented as means ± SD (*p < 0.05, ***p < 0.001 one-way ANOVA against SC)


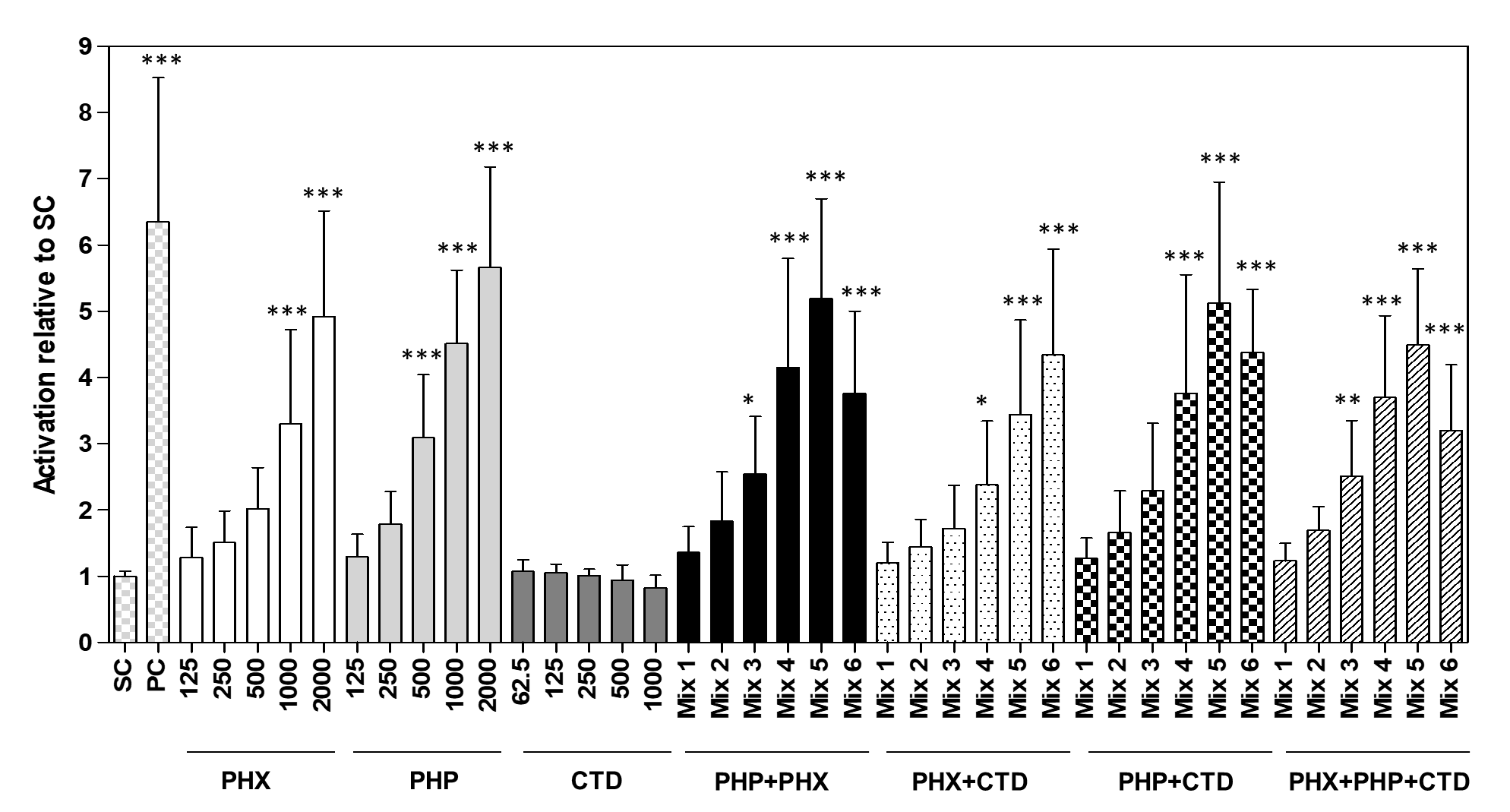


**Fig. S21** RXRα activation by PHX, PHP, CTD and their mixtures. HepG2 cells were co-transfected with the appropriated LBD-expressing plasmid, a GAL4-(UAS)5-TK-LUC reporter plasmid as well as with Renilla luciferase plasmid coding pcDNA3-Rluc for normalization and exposed to indicated concentrations of test compounds, solvent control (SC; 0.5% DMSO) or to the positive control (referred to Table S1). After 24 h cell lysate were assayed for firefly and Renilla luciferase activity. Firefly luciferase activity was normalized against Renilla luciferase activity and fold induction relative to solvent control was calculated. Data are presented as means ± SD (*p < 0.05, **p < 0.01, ***p < 0.001 one-way ANOVA against SC)


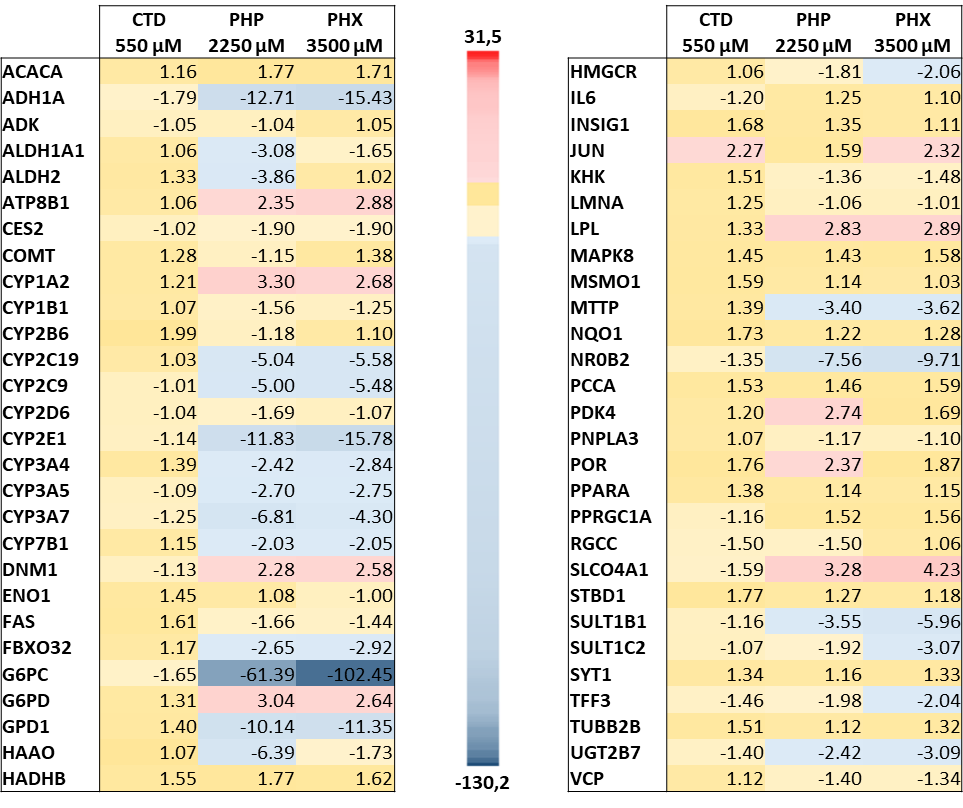


**Fig. S22** Gene expression analysis linked to xenobiotic metabolism, hepatotoxicity and NR activation of PHP, PHX or CTD. 56 genes were selected for PCR analysis in HepaRG cells treated with the EC50 dose of PHP, PHX or CTD for 24 h to screen for deregulated gene expression (screening process). The heat map presents mean fold changes of three biological replicates. Fold changes ≥ 2 and ≤ -2 are highlighted in red and blue, respectively.


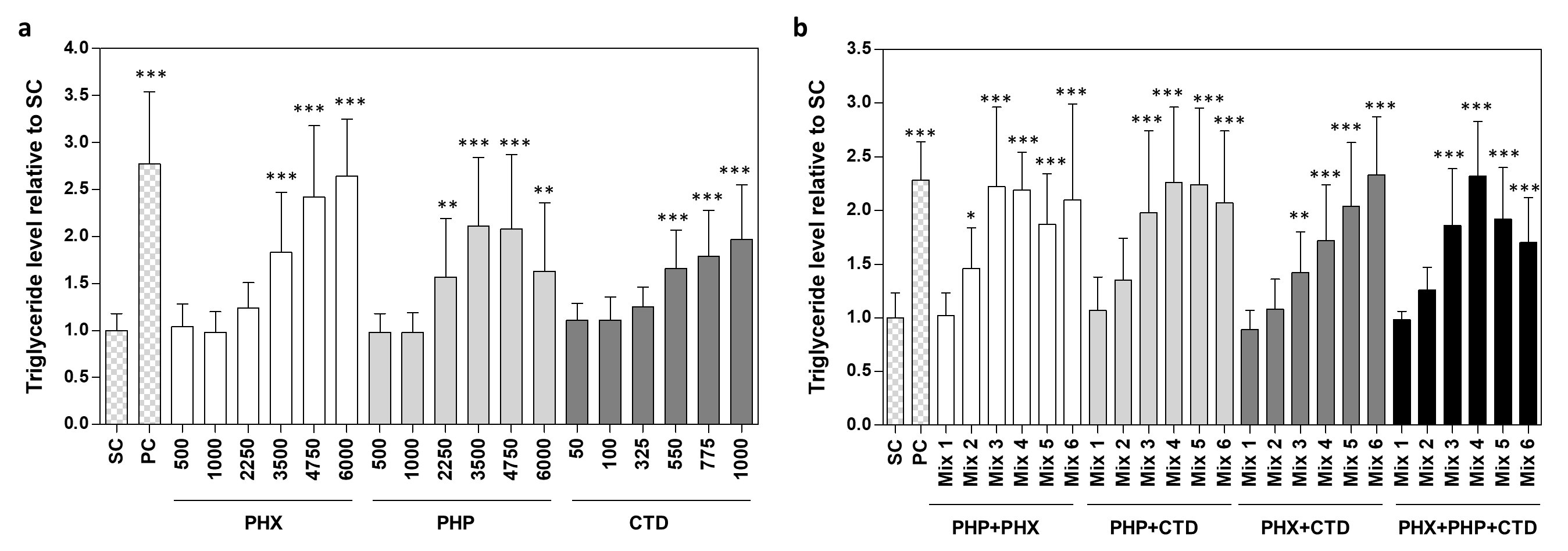


**Fig. S23** Triglyceride accumulation assayed via AdipoRed staining of HepaRG cells exposed to PHX, PHP and CTD (a) or their mixtures (b) for 72 h. Differentiated HepaRG cells were exposed to the different concentrations (referred to Table 1) of pesticides, solvent control (SC; 0.5% DMSO) or positive control (PC; 200 µM cyproconazol) and triglyceride accumulation was analyzed using AdipoRed assay. The triglyceride content was referred to the solvent control. Data are presented as means of three independent experiments performed with three replicates each ± SD (*p < 0.05, **p < 0.01, ***p < 0.001 one-way ANOVA against SC)


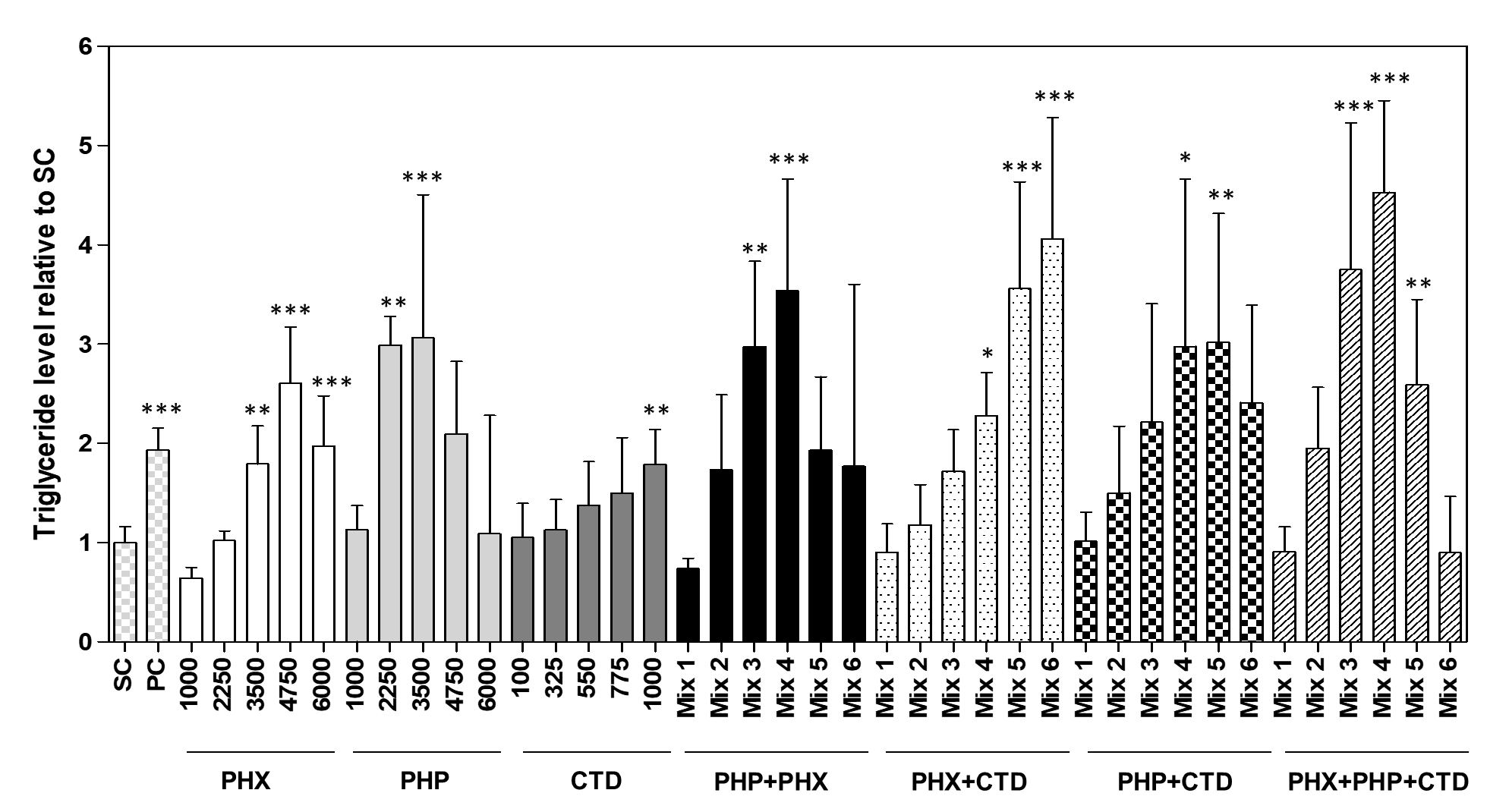


**Fig. S24** Intracellular triglyceride accumulation C44 determined via GC-FID for the single substance PHX. Differentiated HepaRG cells were exposed to different concentrations (referred to Table 1) of PHX, solvent control (SC; 0.5% DMSO) or positive control (PC; 200 µM cyproconazol) for 72 h. The triglyceride content was normalized against the solvent control. Data represents means ± SD (*p < 0.05, **p < 0.01, ***p < 0.001 one-way ANOVA against SC)


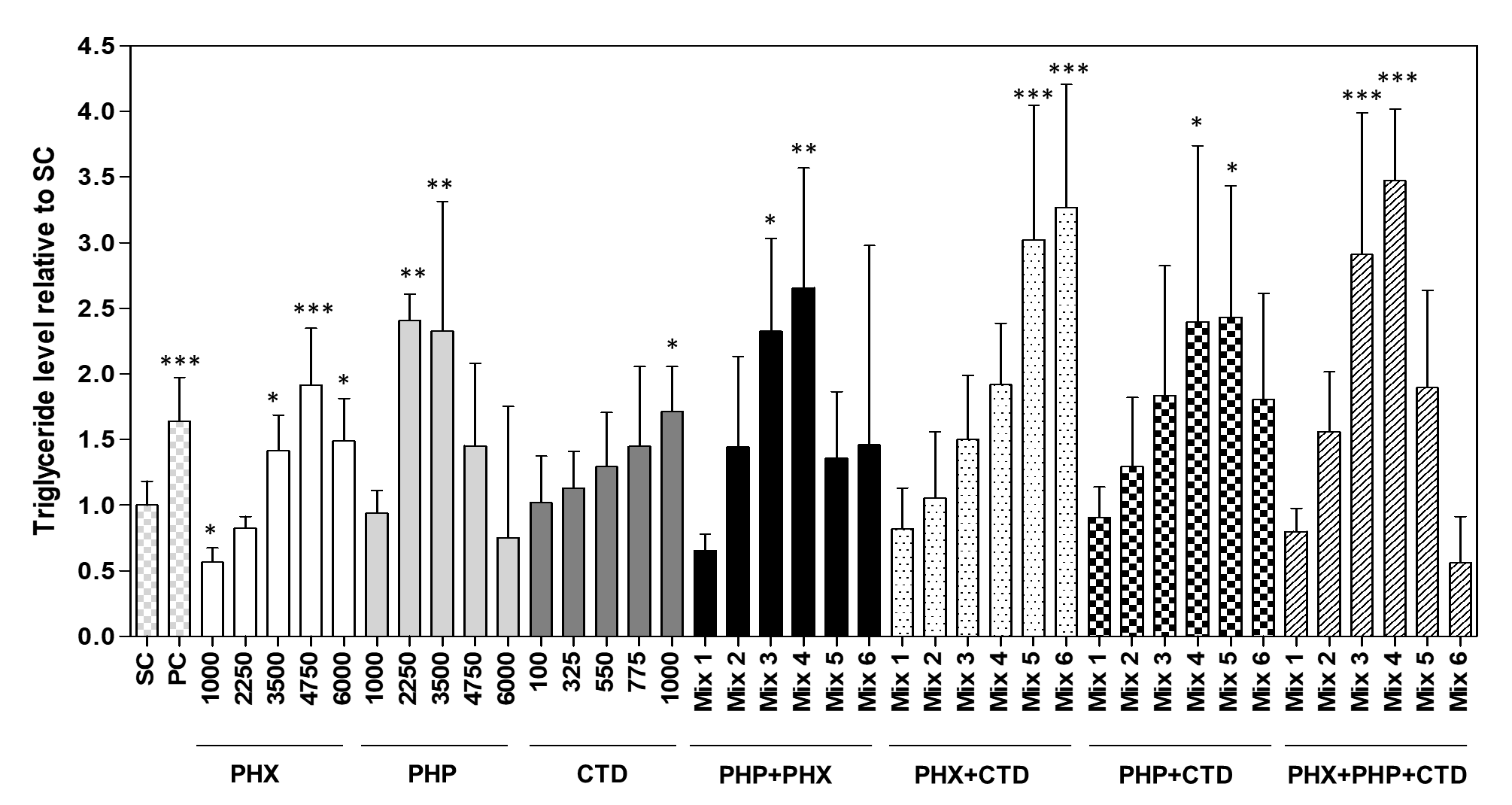


**Fig. S25** Intracellular triglyceride accumulation C46 determined via GC-FID for the single substance PHP. Differentiated HepaRG cells were exposed to different concentrations (referred to Table 1) of PHP, solvent control (SC; 0.5% DMSO) or positive control (PC; 200 µM cyproconazol) for 72 h. The triglyceride content was normalized against the solvent control. Data represents means ± SD (*p < 0.05, **p < 0.01, ***p < 0.001 one-way ANOVA against SC)


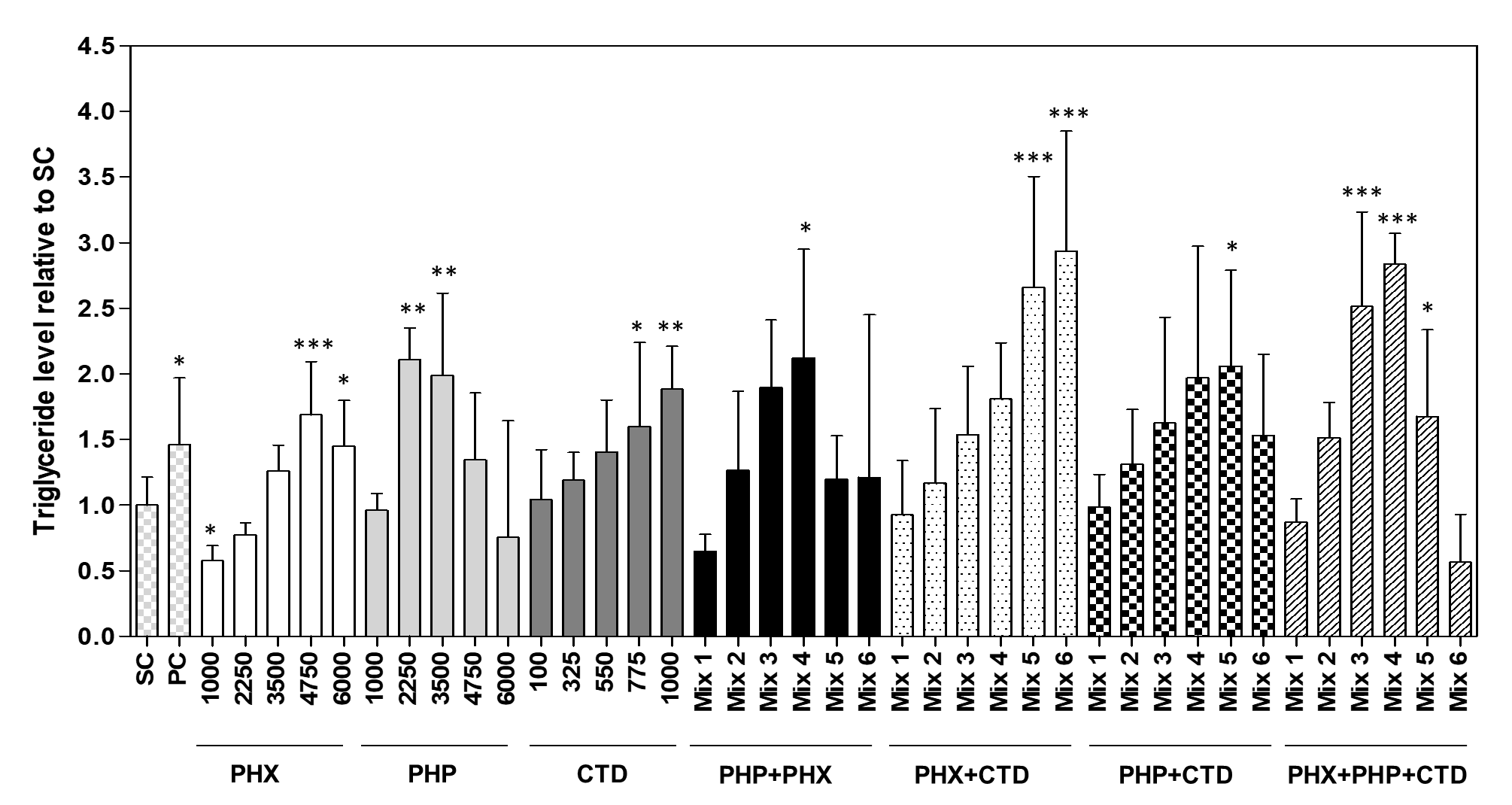


**Fig. S26** Intracellular triglyceride accumulation C48 determined via GC-FID for the single substance CTD. Differentiated HepaRG cells were exposed to different concentrations (referred to Table 1) of CTD, solvent control (SC; 0.5% DMSO) or positive control (PC; 200 µM cyproconazol) for 72 h. The triglyceride content was normalized against the solvent control. Data represents means ± SD (*p < 0.05, **p < 0.01, ***p < 0.001 one-way ANOVA against SC)


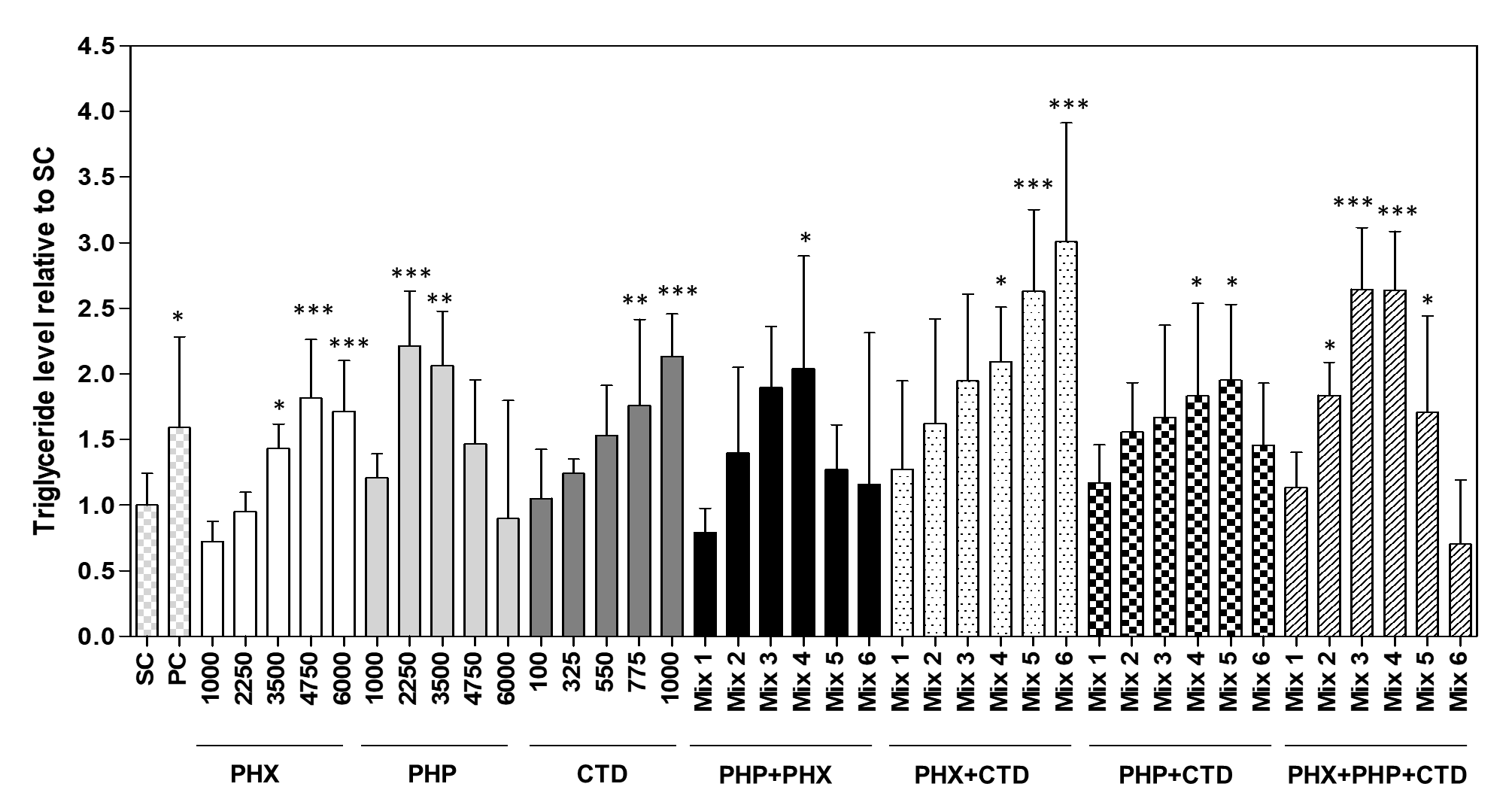


**Fig. S27** Intracellular triglyceride accumulation C50 determined via GC-FID for the binary mixture of PHP and PHX. Differentiated HepaRG cells were exposed to different concentrations (referred to Table 1) of test compounds, solvent control (SC; 0.5% DMSO) or positive control (PC; 200 µM cyproconazol) for 72 h. The triglyceride content was normalized against the solvent control. Data represents means ± SD (*p < 0.05, **p < 0.01, ***p < 0.001 one-way ANOVA against SC)


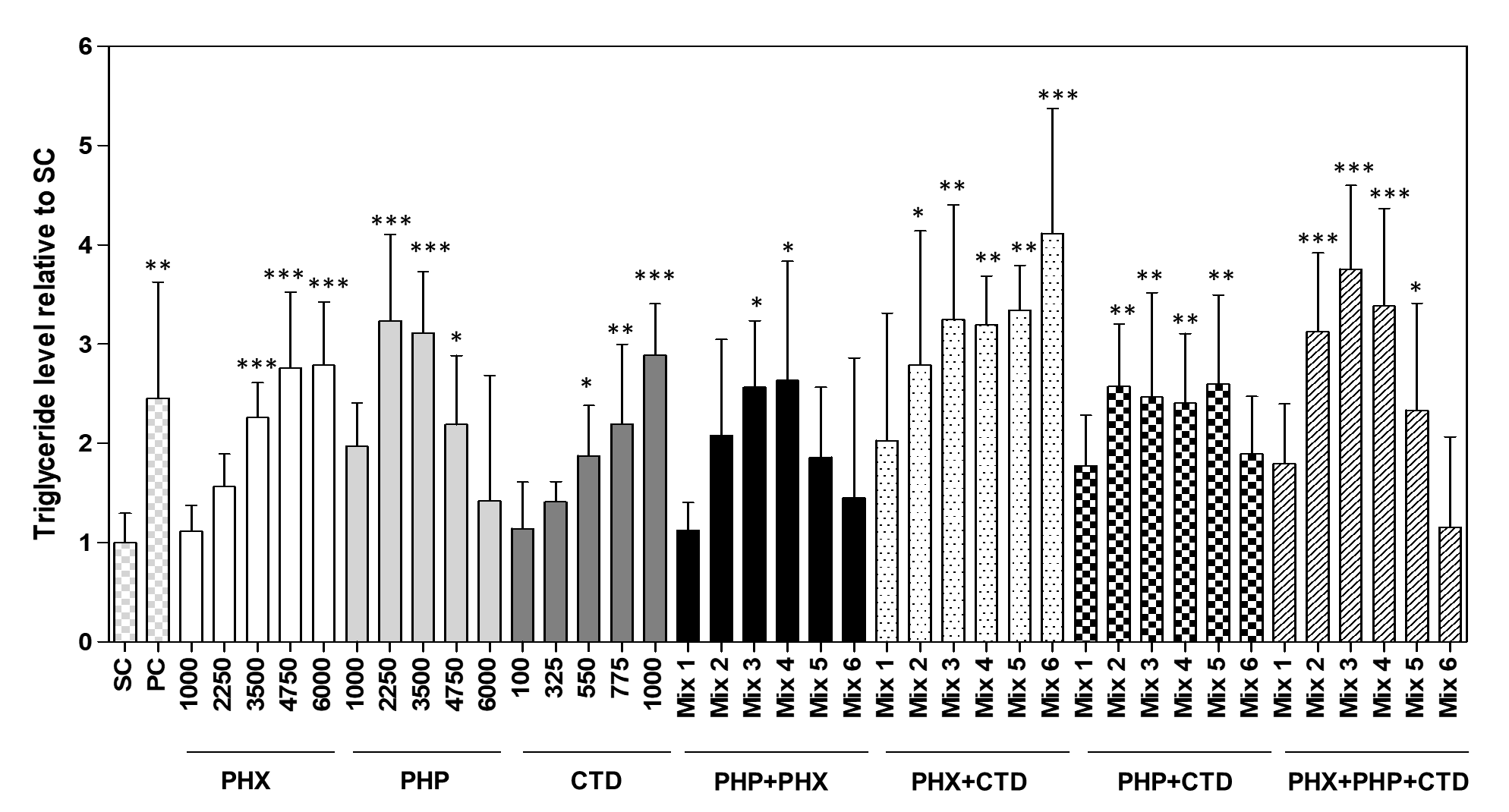


**Fig. S28** Intracellular triglyceride accumulation C52 determined via GC-FID for the binary mixture of PHX and CTD. Differentiated HepaRG cells were exposed to different concentrations (referred to Table 1) of test compounds, solvent control (SC; 0.5% DMSO) or positive control (PC; 200 µM cyproconazol) for 72 h. The triglyceride content was normalized against the solvent control. Data represents means ± SD (*p < 0.05, **p < 0.01, ***p < 0.001 one-way ANOVA against SC)


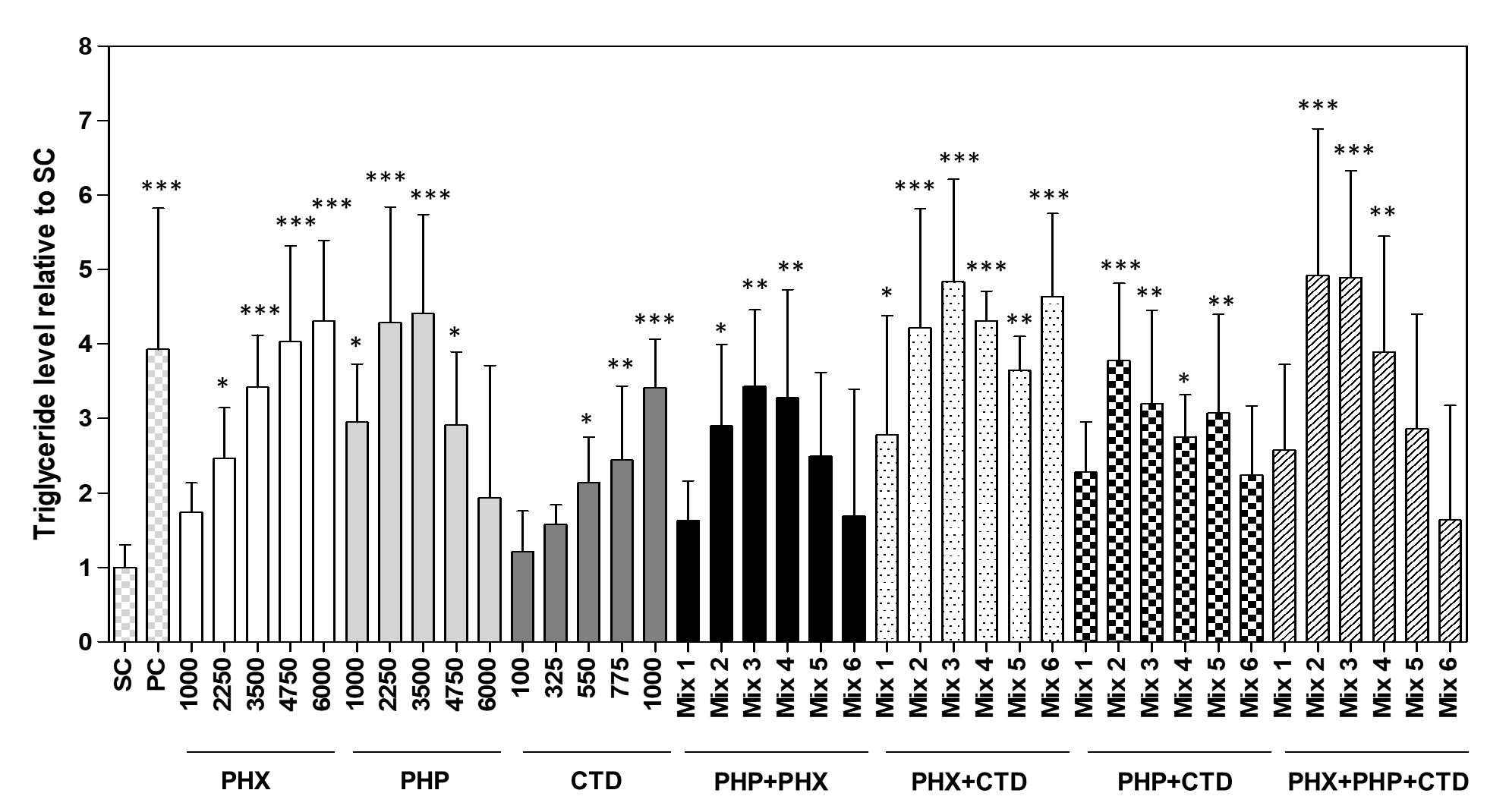


**Fig. S29** Intracellular triglyceride accumulation C54 determined via GC-FID for the binary mixture of PHP and CTD. Differentiated HepaRG cells were exposed to different concentrations (referred to Table 1) of test compounds, solvent control (SC; 0.5% DMSO) or positive control (PC; 200 µM cyproconazol) for 72 h. The triglyceride content was normalized against the solvent control. Data represents means ± SD (*p < 0.05, **p < 0.01, ***p < 0.001 one-way ANOVA against SC)

**
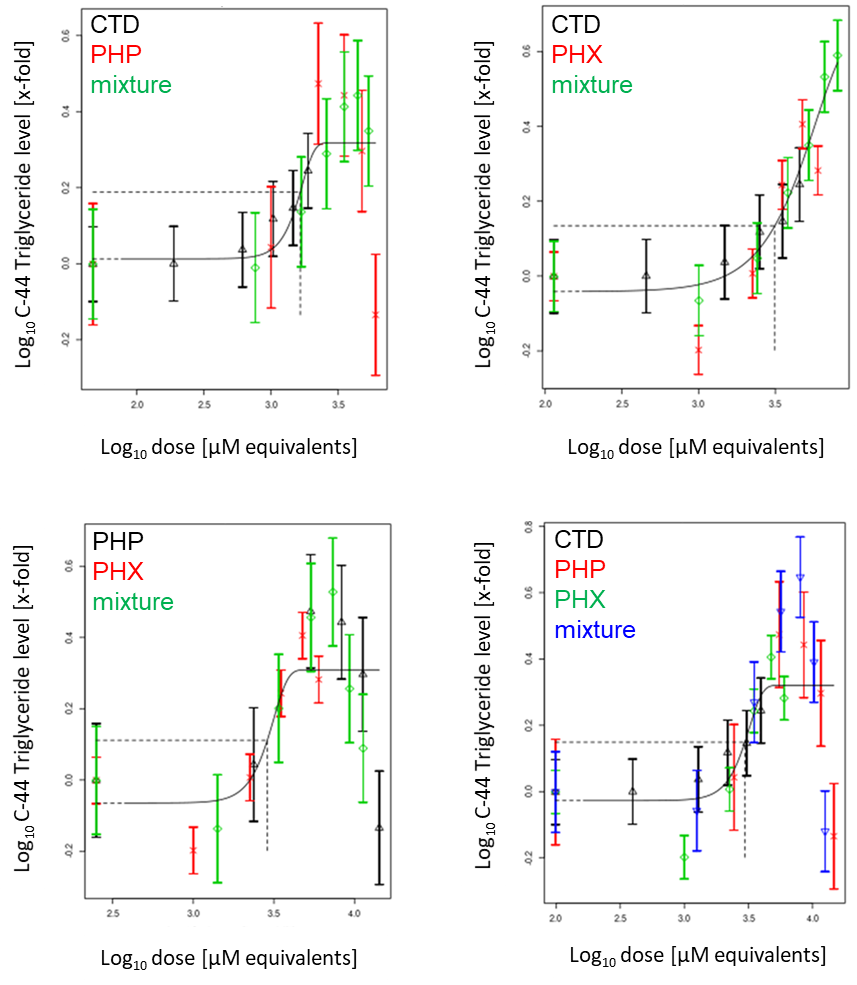
**

**Fig. S30** Representative concentration-response modeling of triglyceride accumulation (C44), as determined by GC-FID for all different mixtures based on the data shown in Fig. S24. The curves represent the four-parameter exponential model; see equation 1. The concentration-response of the mixture (green diamonds) indicate no deviation from the overall concentration-response fit. Thus, dose addition can be assumed.

**
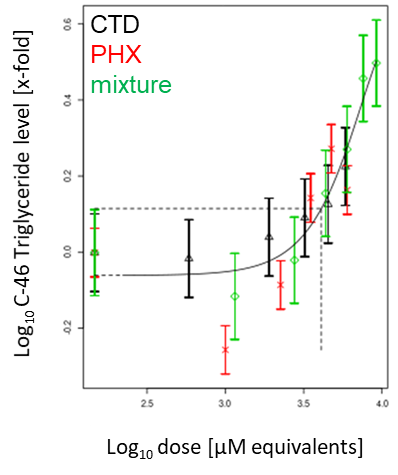
**

**Fig. S31** Representative concentration-response modeling of triglyceride accumulation (C46), as determined by GC-FID for CTD+PHX mixture based on the data shown in Fig. S25. The curves represent the four-parameter exponential model; see equation 1. The concentration-response of the mixture (green diamonds) indicate no deviation from the overall concentration-response fit. Thus, dose addition can be assumed.

**
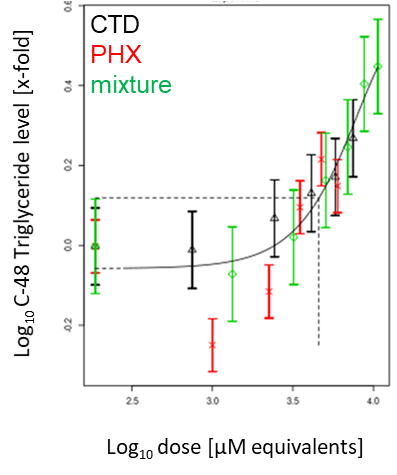
**

**Fig. S32** Representative concentration-response modeling of triglyceride accumulation (C48), as determined by GC-FID for CTD+PHX mixture based on the data shown in Fig. S26. The curves represent the four-parameter exponential model; see equation 1. The concentration-response of the mixture (green diamonds) indicate no deviation from the overall concentration-response fit. Thus, dose addition can be assumed.

**
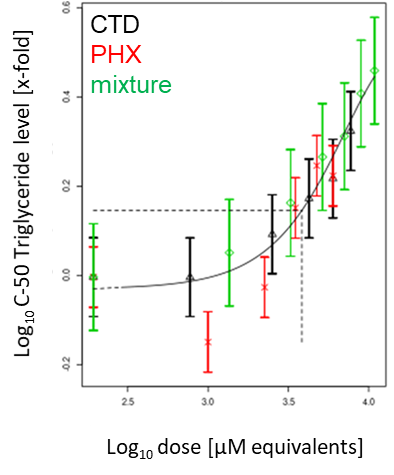
**

**Fig. S33** Representative concentration-response modeling of triglyceride accumulation (C50), as determined by GC-FID for CTD+PHX mixture based on the data shown in Fig. S27. The curves represent the four-parameter exponential model; see equation 1. The concentration-response of the mixture (green diamonds) indicate no deviation from the overall concentration-response fit. Thus, dose addition can be assumed

**
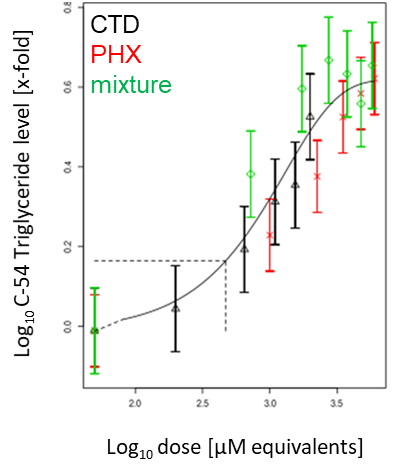
**

**Fig. S34** Representative concentration-response modeling of triglyceride accumulation (C54), as determined by GC-FID for CTD+PHX mixture based on the data shown in Fig. S29. The curves represent the four-parameter exponential model; see equation 1. The concentration-response of the mixture (green diamonds) indicate no deviation from the overall concentration-response fit. Thus, dose addition can be assumed

**a**

**
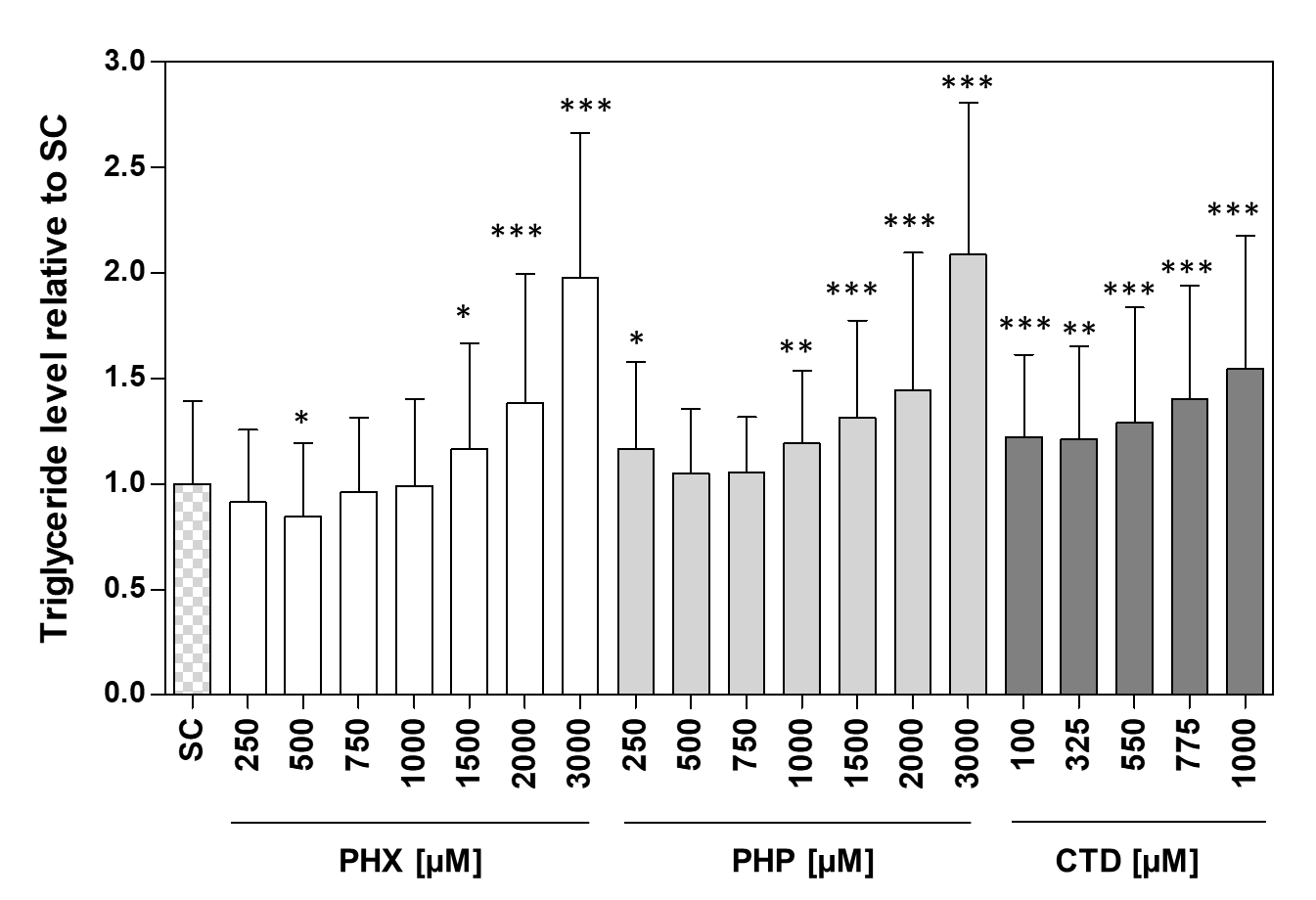
**

**b**

**
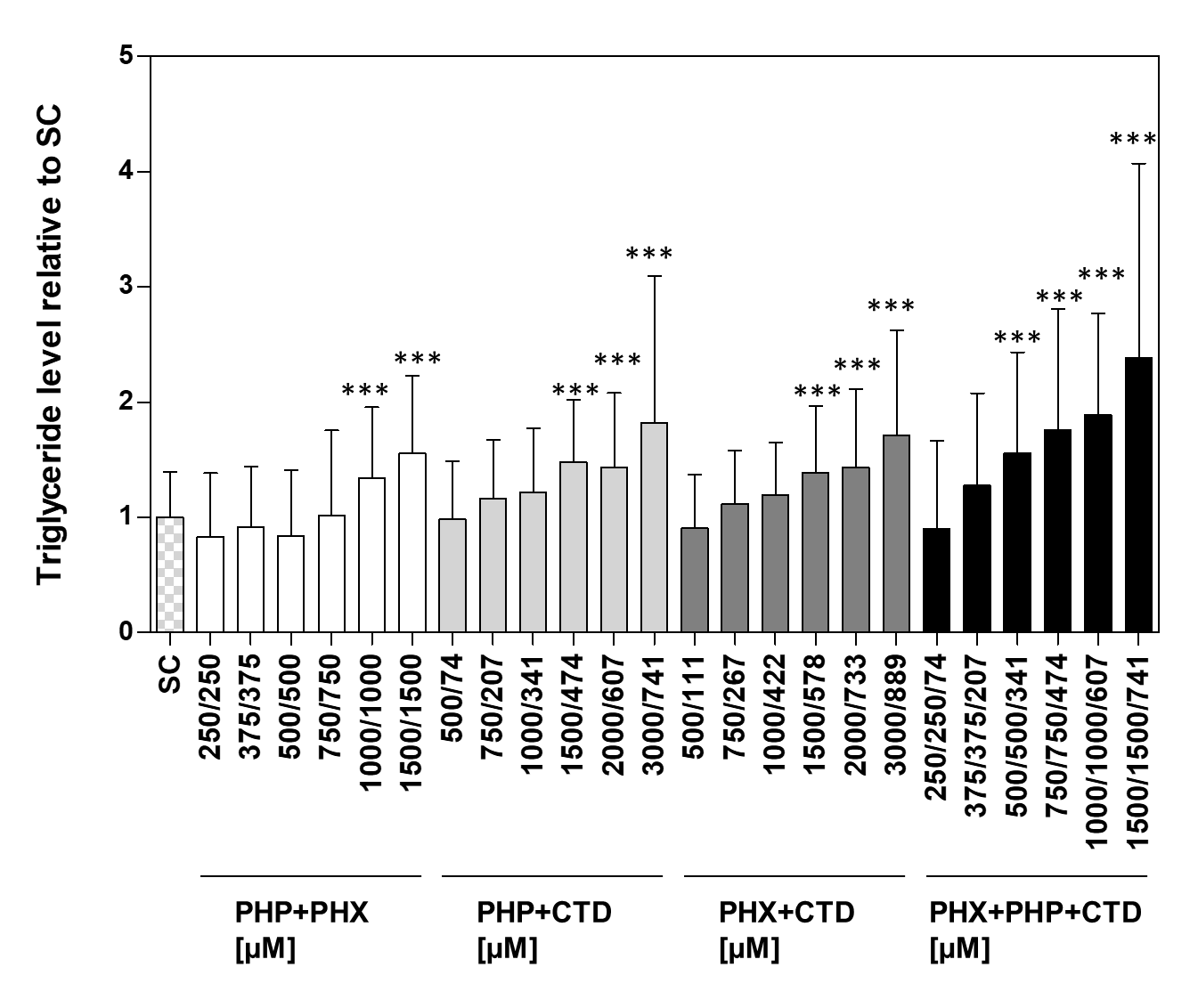
**

**Fig. S35** Intracellular triglyceride accumulation, determined via high-content cell imaging of neutral lipid droplets at the single cell level. Differentiated HepaRG cells were exposed to different concentrations of PHX, PHP and CTD (a), their mixtures (b), or the solvent control (SC: 0.5% DMSO) for 72 h. The triglyceride content was normalized against the solvent control. Data represents means ± SD (*p < 0.05, **p < 0.01, ***p < 0.001 one-way ANOVA against SC)
